# Synthesis of tetrasubstituted 1-silyloxy-3-aminobutadienes and chemistry beyond Diels–Alder reactions

**DOI:** 10.1038/ncomms7913

**Published:** 2015-04-21

**Authors:** Xijian Li, Siyu Peng, Li Li, Yong Huang

**Affiliations:** 1 Key Laboratory of Chemical Genomics, Peking University, Shenzhen Graduate School, Shenzhen 518055, China

## Abstract

Electron-rich dienes have revolutionized the synthesis of complex compounds since the discovery of the legendary Diels–Alder cycloaddition reaction. This highly efficient bond-forming process has served as a fundamental strategy to assemble many structurally formidable molecules. Amino silyloxy butadienes are arguably the most reactive diene species that are isolable and bottleable. Since the pioneering discovery by Rawal, 1-amino-3-silyloxybutadienes have been found to undergo cycloaddition reactions with unparalleled mildness, leading to significant advances in both asymmetric catalysis and total synthesis of biologically active natural products. In sharp contrast, this class of highly electron-rich conjugated olefins has not been studied in non-cycloaddition reactions. Here we report a simple synthesis of tetrasubstituted 1-silyloxy-3-aminobutadienes, a complementarily substituted Rawal's diene. This family of molecules is found to undergo a series of intriguing chemical transformations orthogonal to cycloaddition reactions. Structurally diverse polysubstituted ring architectures are established in one step from these dienes.

## Abstract (web summary)

Electron-rich dienes have been shown to have multiple uses in synthesis by means of cycloaddition reactions. Here, the authors report a series of non-cycloaddition-based reactions of these compounds, giving access to 4-, 5-, 6- and 7-membered ring systems.

## Graphical abstract

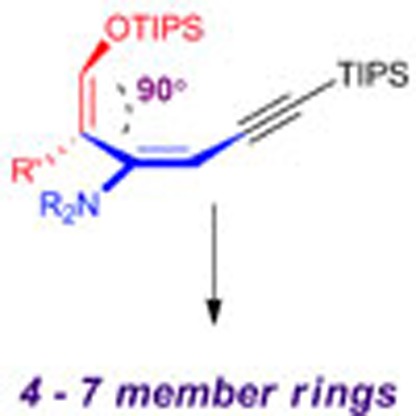

Amino silyloxy butadienes are a class of highly reactive compounds that have demonstrated tremendous utility in natural product synthesis^1^,^2^,^3^,^4^,^5^,^6^,^7^,^8^ and asymmetric catalysis^9^,^10^,^11^,^12^,^13^. In 1997, Rawal and colleagues^14^,^15^ reported the first general synthesis of 1-amino-3-silyloxybutadienes from vinylogous amides. This family of highly reactive species showed significantly enhanced reactivity compared with the other electron-rich dienes in a number of cycloaddition reactions^16^,^17^. The extraordinary reactivity of the Rawal's diene is a result of an exceptionally high and polarized electron density along the four carbon conjugation system. Besides the Rawal's diene, other amino silyloxydienes were also reported, most of which bear the 1-amino-3-silyloxy substitution pattern^18^,^19^,^20^. In comparison, the complementarily substituted 1-silyloxy-3-aminodienes, which have a similar electronic property as their 1-amino-3-silyloxy counterparts, have been much less studied^21^,^22^. Schlessinger and Tsuge reported that 1-silyloxy-3-aminobutadienes could be prepared by γ-silylation of vinylogous amides or carbamates, and they underwent facile Diels–Alder reactions. Among these amino silyloxy butadienes, tetrasubstituted analogues are very rare. In addition, amino silyloxy butadienes have received very little attention outside the cycloaddition paradigm. Considering the versatile functionalities embedded in these highly electron-rich molecules, developing efficient synthesis of 1-silyloxy-3-aminodienes and exploring transformations beyond cycloadditions would lead to significantly broadened synthetic utility of these versatile intermediates.

Herein, we report a synthesis of tetrasubstituted 1-silyloxy-3-aminobutadienes from readily available allenyl aldehydes and secondary amines. The reaction occurs under mild conditions in the absence of any catalyst and is highly atom economical. These dienes are discovered to undergo a number of captivating transformations other than cycloadditions that lead to a wide range of heavily substituted ring structures.

## Results

### Serendipitous synthesis of 1-silyloxy-3-aminobutadiene

**Figure 1:**
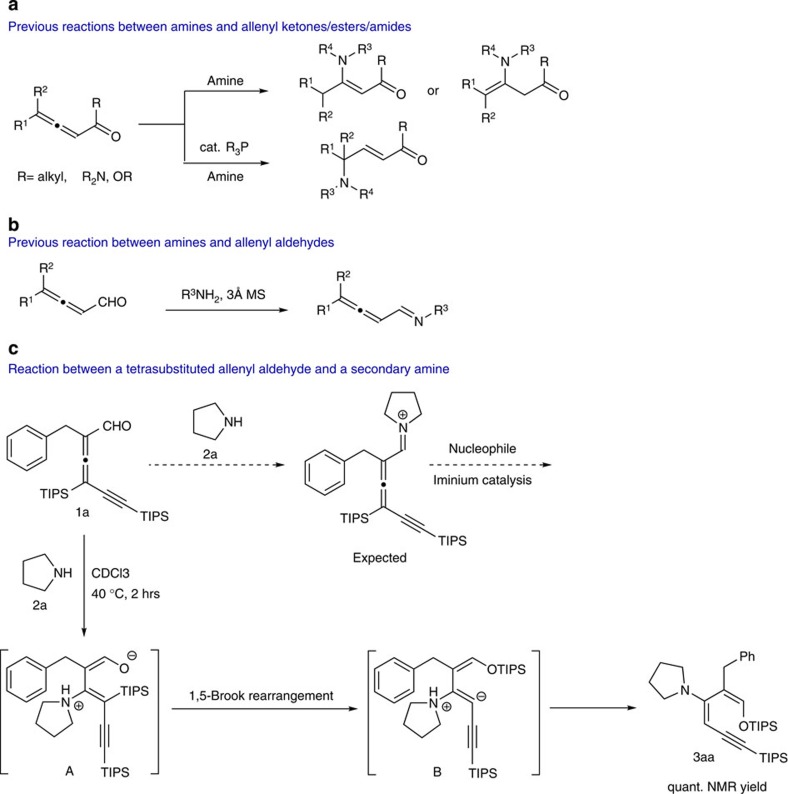
Chemistry of allenyl carbonyls and amines. (**a**) Conjugate addition of amines and phosphines to allenyl ketones, esters and amides are well established in the literature^25^,^26^. (**b**) Trisubstituted allenyl aldehydes react with primary amines to give the imine products. (**c**) Reaction between the tetrasubstituted allenyl aldehyde **1a** and pyrrolidine **2a** generated substituted 1-amino-3-silyloxybutadiene **3aa** in high yield via a conjugate addition, 1,5-Brook rearrangement and protonation sequence. TIPS, triisopropylsilyl.

We recently developed a one-step synthesis of tri- and tetrasubstituted allenyl aldehydes from simple aldehydes and an electrophilic alkynylation reagent using gold/amine synergistic catalysis^23^. We found that a trisubstituted allenyl aldehyde reacted readily with an amine to form an ynenamine intermediate, which could be intercepted by various electrophiles including molecular oxygen^24^. Subsequently, we decided to explore a reaction between a tetrasubstituted allenyl aldehyde and a secondary amine. We were hoping that they would form a highly reactive allenyl iminium species that might promote novel chemical transformations with nucleophiles. Surprisingly, although the conjugate addition of amines to the *sp* carbon of allenyl ketones, esters and amides were well known in the literature (Fig. 1a)^25^,^26^, reactions involving the much more labile allenyl aldehydes were much less explored. There is only one report in which allenyl aldehydes readily formed the corresponding imines (1,2-addition) when treated with primary amines (Fig. 1b)^27^. No information was found for reactions involving allenyl aldehydes and secondary amines.

Surprisingly, the desired iminium intermediate was not observed when allenyl aldehyde **1a** was treated with 1 equiv. pyrrolidine **2a** under various conditions. Instead, a diene product **3aa** was formed in quantitative yield (Fig. 1c). Nuclear Overhauser Effect studies showed that the silyl enol ether existed as the *Z*-isomer. On the basis of this information, we proposed that **3aa** was formed by the conjugate addition of pyrrolidine **2a** to allenyl aldehyde **1a**, followed by a facile 1,5-Brook rearrangement^28^. This reaction proceeded well in a number of aprotic solvents. Although prolonged exposure to silica gel led to decomposition, the zero-byproduct nature of this reaction made the work-up extremely easy. Quick filtration through a plug of neutral Al_2_O_3_ afforded the product in good purity. In some cases, simple removal of solvent gave essentially pure product.

### Reaction scope of allenyl aldehyde

The scope of allenyl aldehyde was examined next. Various substrates were prepared directly from commercially available aldehydes and were subjected to pyrrolidine in dichloromethane (DCM) at 40 °C (Table 1). β-Aryl substituents were well tolerated. Good to excellent yields were obtained uniformly. Allenyl aldehydes from simple alkyl aldehydes were also explored. Alkyl groups bearing various functional groups (ether, ester, imide, olefin and sulfamide and so on) were excellent substrates. The reaction became slow when a sterically hindered R group was introduced. For example, only a trace amount of the corresponding 1-silyloxy-3-aminobutadiene was observed at elevated temperature after overnight when a secondary alkyl group is attached to the α-carbon of the allenyl aldehyde.

### Reaction scope of amine

The scope of amine was also investigated (Table 2). A number of cyclic and acyclic secondary amines worked well for this reaction. Double bond, hydroxyl, carbamate and tertiary amine functionalities did not interfere with the diene formation. However, the reaction rate slowed down when hindered secondary amines were employed (Table 2, **3fa**, **3ha**, **3na**, **3oa**). Excess amine (2 equiv.) was used along with prolonged reaction time for those substrates. The reaction did not proceed for very bulky dialkyl amines such as diisopropylamine. Although no iminium formation was observed for secondary amines, the 1,2-addition was the major reaction pathway for primary amines.

### Structure of the 1-silyloxybutadiene and its implication

**Figure 2:**
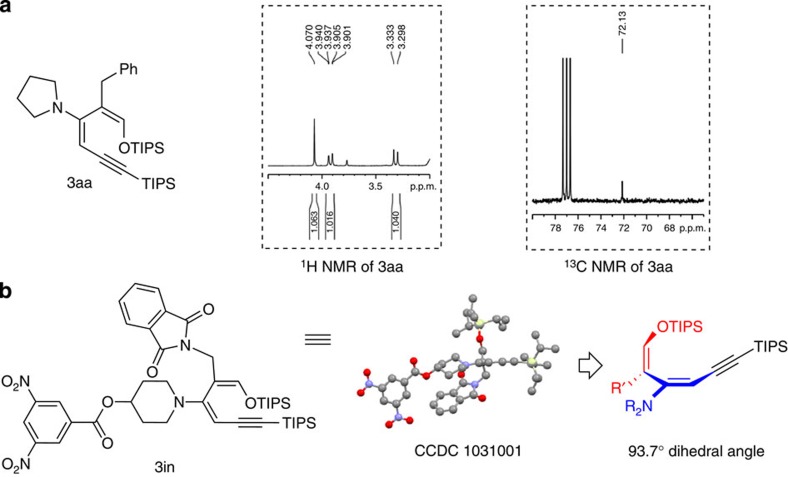
Structural information of the 1-silyloxy-3-aminobutadiene. (**a**) NMR indicated highly eletron-rich nature of the enamine moiety. (**b**) X-ray structure revealed an unusual twisting of the diene olefinic carbons.

1-Silyloxy-3-aminobutadiene **3aa** showed an HNMR singlet signal at 4.07 and a CNMR signal at 72.13, an indication of a highly electron-rich enamine olefinic carbon (Fig. 2a). The structure of the diene was subsequently determined by X-ray using a phthalyl-substituted analogue **3in**. Interestingly, the diene was severely distorted out of conjugation in this structure, with the two double bonds nearly perpendicular to each other (Fig. 2b). This unexpected structure of the diene suggested that these dienes might not be suitable for the Diels–Alder reactions due to twisted conjugation. Various dienophiles were examined and no cycloadduct was formed under either thermal or Lewis acid catalysis conditions. In view of the electron-rich silyl enol ether and enamine present in these molecules, we decided to explore opportunities to affect novel transformations of **3aa** using electrophiles. Several intriguing transformations were discovered and a series of highly substituted small ring structures were generated.

### Cyclization to form cyclobutenes

**Figure 3:**
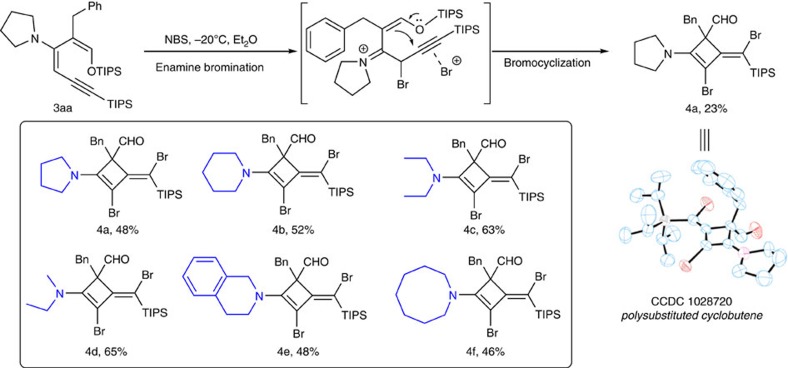
Formation of highly substituted cyclobutenes. The reaction is believed to proceed through an α-bromination/cyclization pathway. The structure of the product was assigned by X-ray. The products in the box were synthesized using 0.05 mmol dienes and 0.1 mmol *N*-bromosuccinimide (NBS) in 1 ml dichloromethane for 2 h; isolated yield.

When **3aa** was treated with *N*-bromosuccinimide (NBS) at low temperature in ether, a dibromo cyclobutenyl aldehyde **4aa** was formed in 23% yield (Fig. 3). Cyclobutenes are quite unstable due to high ring strain and preparation of highly substituted cyclobutenes structures have been very challenging. Presumably, bromination of the enamine generated an α-bromo iminium intermediate that would rotate the π*-orbital of the alkyne to overlap with π-electron pair of the silyl enol ether to engage in a facile electrophilic ring closure. The exocyclic olefin geometry of the product was fully controlled as the corresponding *E*-isomer.

This reaction was optimized using different solvents. Very little product was formed in tetrahydrofuran, toluene and ethyl acetate. Decent conversion was obtained when the reaction was carried out at −20 °C in dichloromethane. The desired product was isolated in 48% yield. This reaction was general for various amines and exo-olefinic cyclobutenes bearing different nitrogen substituents were prepared in moderate yields (Fig. 3). Bromocyclization reactions are well known for heteroatom nucleophiles, with five- and six-membered rings being the most common products^29^,^30^,^31^,^32^,^33^. Our reaction represents a rare example where a four-membered ring is formed using a carbon nucleophile. The intriguing structure of **4a** offers potential chemical viability to novel cyclobutane scaffolds.

### Imino-Nazarov cyclization to form cyclopentenone

**Figure 4:**
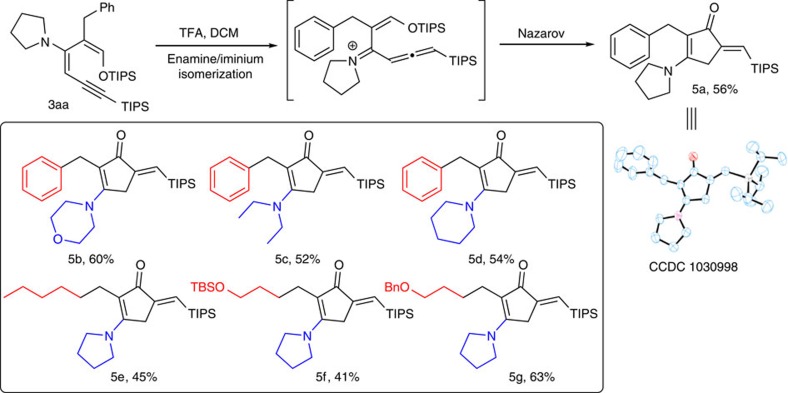
Formation of the exo-olefinic cyclopentenone product. (**a**) Reactions were conducted with 0.05 mmol dienes in 1 ml solvent; isolated yield. (**b**) It is interesting that the closing ends of the iminium intermediates are both electron deficient. (**c**) Condition screening: MeSO_3_H (1.1 equiv.), DCM, 30%; CF_3_SO_3_H (1.1 equiv.), DCM, 35%; trifluoroacetic acid (TFA, 1.1 equiv.), DCM, 56%; TFA (1.1 equiv.), Et_2_O, 49%; TFA (1.1 equiv.), tetrahydrofuran (THF), 33%; TFA (0.5 equiv.), DCM, 30%; TFA (2.0 equiv.), DCM, 28%; TFA (5.0 equiv.), DCM, trace. (**d**) The products in the box were synthesized using 0.05 mmol dienes and 0.055 mmol TFA in 1 ml dichloromethane; isolated yield.

On treatment with an acid, the ynenamine moiety of diene **3aa** was converted to allenyl iminium by γ-protonation^34^, which experienced a cationic 4-π eletrocyclization to give **5aa** as a single isomer (Fig. 4). This type of imino-Nazarov cyclization involving allene is very rare in the literature, and often occurs with poor selectivity and yield^35^. We subsequently found that the reaction was very sensitive to the acid used. Methanesulfonic acid and trifluoromethanesulfonic acid afforded poor yields. TFA was later found to be the best proton source. Solvent and acid stoichiometry were examined next. A catalytic amount of TFA led to an incomplete conversion and excess acid led to diene decomposition. Eventually, 1.1 equiv. TFA afforded the exo-olefinic cyclopentenone product in 56% isolated yield. The key exo-olefinic cyclopentenone moiety in product **5** is often found in methylenomycin and prostaglandin families of natural products and drugs^36^. The substrate scope was investigated in a broad manner (Fig. 4). Various 2-alkyl and 3-amino groups were well tolerated. The reaction condition was mild enough to retain acid labile functionalities, such as TBS ether. The TIPS group could be removed readily using TBAF. The existence of many orthogonal functional groups in these products offers opportunities for versatile structural manipulation.

### Carbonylation to form pyranone

**Figure 5:**
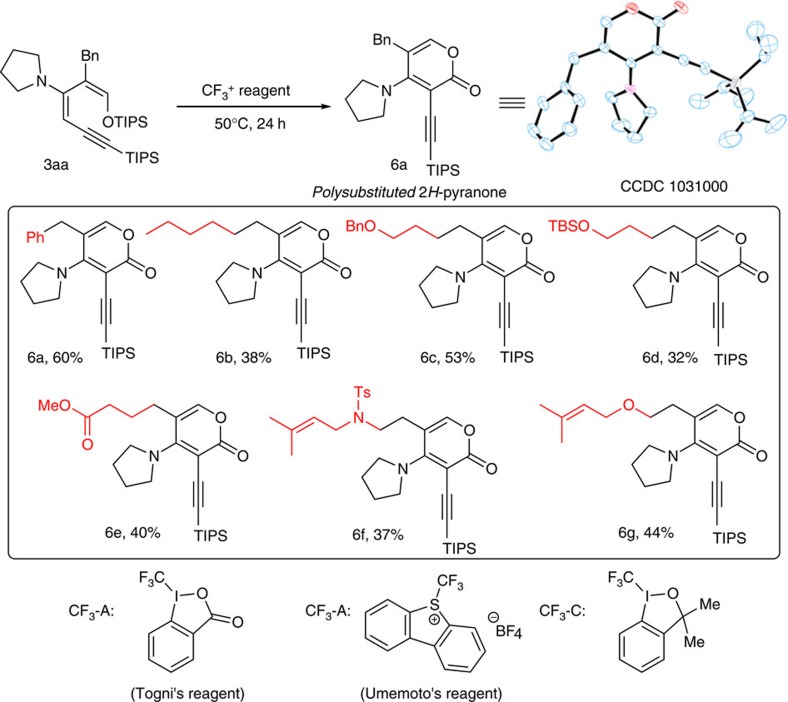
Formation of pyranone and condition screening. (**a**) Condition screening: CF_3_-A, Et_2_O, no product; CF_3_-A, tetrahydrofuran (THF), trace; CF_3_-A, Toluene; trace; CF_3_-A, DCE, trace; CF_3_-A, CHCl_3_, 22%; CF_3_-A, CH_2_Cl_2_, 60%; CF_3_-B, CH_2_Cl_2_, 17%; CF_3_-C, CH_2_Cl_2_, trace. (**b**) The products in the box were synthesized using 0.05 mmol diene and 0.05 mmol CF_3_-A in 1 ml CH_2_Cl_2_ at 50 °C for 24 h; isolated yield. (**c**) For product **6f**, the reaction temperature was 90 °C.

When diene **3aa** was treated with electrophilic trifluoromethylating reagent, we were very surprised to find that a six-membered pyranone product **6a** was formed (Fig. 5). This reaction was optimized immediately. We found that neutral hypervalent trifluoromethyl iodide (Togni's reagent) performed better than CF_3_ salts, such as the Umemoto reagent. The reaction exhibited a strong solvent effect. No or trace product was observed in ether, tetrahydrofuran, toluene and dichloroethane. Interestingly, there is a large discrepancy between chloroform and dichloromethane. The conversion was much higher in dichloromethane, with 60% isolated yield. With the optimized reaction condition in hand, we examined the generality of the pyranone formation using 1-silyloxy-3-aminobutadienes bearing various substituents. It was found that alkyl group at the second position of the dienes were particularly well tolerated. Common functionality groups, such as ether, ester, olefin, silicon and sulfonamide, were successfully incorporated in the pyranone product (Fig. 5). Further investigation of this transformation using simple substrates is currently underway to better understand the structural requirement for such a delicate transformation.

**Figure 6:**
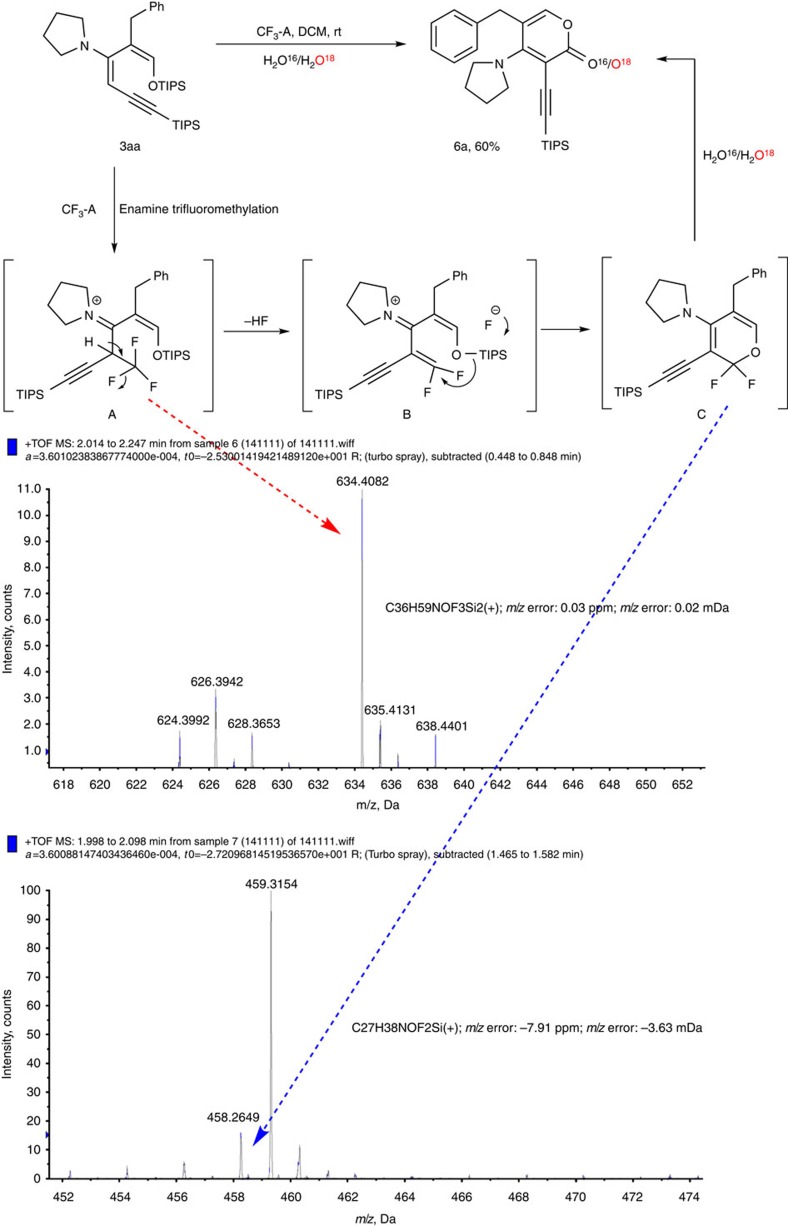
Proposed mechanism for the formation of six-membered ring. (**a**) For the isotope experiment, dichloromethane was shaken rigorously with H_2_^18^O and subsequently used for the reaction. (**b**) The enamine functionality is the primary reason for the facile conversion of the CF_3_ group to the carbonyl, as it pushes an electron pair towards CF_3_ and triggers the elimination of a fluoride anion. The alkyne moiety is also believed to be important for stabilizing the adjacent enamine.

The formation of pyranone **6** is very interesting and highly unexpected. Careful control experiments showed that the reaction was very sensitive to the amount of water in the system. In the absence of water, very little product was observed. With excess water, the starting material decomposed quickly. HRMS showed that when the solvent was saturated with H_2_^18^O, the product was formed with significant heavy isotope enrichment (Fig. 6). In addition, subjecting the reaction mixture to HRMS revealed the existence of a trifluoromethylated intermediate **A** and a difluoro cyclic ether species **C** (Fig. 6). On the basis of these data, we propose the following reaction mechanism: first, trifluoromethylation of the enamine generates α-CF_3_ iminium intermediate **A**. This species has a highly acidic carbon with three electron-withdrawing groups attached—iminium, alkyne and CF_3_, which likely results a rapid elimination of hydrogen fluoride to give difluoro olefin **B**. The release of HF would deprotect the TIPS group, followed by a concurrent cyclization of the enolate to the electron-deficient difluoroalkene to give cyclic difluoroether **C**. Although hydrolysis of CF_2_ to a carbonyl group requires harsh conditions^37^, we believe that the adjacent enamine greatly accelerates this conversion by promoting rapid elimination of fluorides. Notably, this represents the first example that the Togni's reagent is used as a carbonyl precursor.

### Formal [4+3] cycloaddition to form azepine

**Figure 7:**
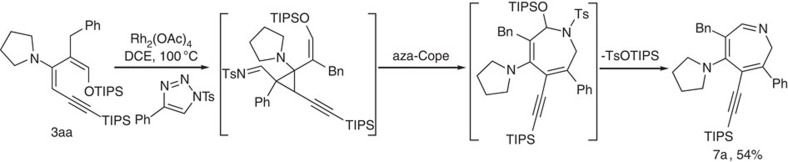
Formation of seven-membered ring. Condition: **3aa** (0.2 mmol), 4-phenyl-1-tosyl-1H-1,2,3-triazole (0.1 mmol), Rh_2_(OAc)_2_ (0.01 mmol), DCE (2 ml), 100 °C, 48 h; isolated yield.

Diene **3aa** was also tested for formal [4+3] cycloaddition reactions. Under rhodium(II) iminocarbene condition, tetrasubstituted azepine **7a**, was obtained in 54% yield (Fig. 7). The structure of this product differed from a recent report using simple dienes (Tang and colleagues^38^) by containing one extra double bond in the already strained seven-member ring as a result of *in situ* elimination of TsOTIPS. No dihydropyrrole product was found in our reaction, which was formed on prolonged heating in Tang's report. The reaction was sensitive to the catalyst and reaction temperature. When changed to Rh(II) complexes other than Rh_2_(OAc)_4_, much lower yields were observed. Although both enamine and silyl enol ether are electron rich, the first cyclopropanation occurred exclusively at the enamine double bond, leading to a single isomer. The subsequent aza-Cope rearrangement was companied by simultaneous loss of TsOTIPS. The versatile functionalities contained in this tetrasubstituted azepine product offered great opportunities for structural diversification.

### Selective functionalization of enamine

**Figure 8:**
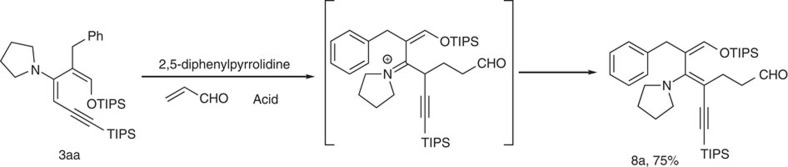
Differentiation of enamine and silyl enol ether. Condition: **3aa** (0.1 mmol), acrolein (0.15 mmol), *rac*-*trans*-2,5-diphenylpyrrolidine (0.02 mmol), 4-Nitrobenzoic acid (0.02 mmol), dichloromethane (2 ml), room temperature, 2 h; isolated yield.

Finally, the nucleophilic reactivity of the silyl enol ether and the enamine could be differentiated using amine catalysed conjugate addition reaction. When diene **3aa** was treated with acrolein in the presence of a hindered secondary amine, the Michael addition product **8aa** was obtained in 75% yield (Fig. 8)^39^. Although 4-nitrobenzoic acid was used, both the enamine and the silyl enol ether moieties were not hydrolysed, suggesting the intrinsic stability of this pentasubsituted diene.

In summary, we developed a general synthesis of highly substituted 1-silyloxy-3-aminobutadienes using a very mild condition from readily available allenyl aldehydes. This unusual diene formation proceeded through an unprecedented conjugate addition of a secondary amine to an allenyl aldehyde, followed by a 1,5-Brook rearrangement. In sharp contrast to other electron-rich dienes, these 1-silyloxy-3-aminobutadienes exhibit extraordinary synthetic versatility for non-cycloaddition reactions. Several unexpected transformations were discovered and studied in detail. Structurally sophisticated four-, five-, six- and seven-membered ring systems bearing multiple functionalities were assembled in one step from these dienes. We expect that this chemistry will stimulate the design of novel synthetic strategies towards complex structures using amino silyloxy butadienes.

## Methods

### General methods and materials

Solvents for reactions were distilled according to general practice before use. All reagents were purchased and used without further purification unless specified otherwise. Allenyl aldehyde substrates were prepared according to the literature-reported procedure^23^. Solvents for chromatography were technical grade and distilled before use. Flash chromatography was performed using 200–300 mesh silica gel with the indicated solvent system according to standard techniques. Analytical thin-layer chromatography was performed using Huanghai silica gel plates with HSGF 254. Qingdao Haiyang Chemical HG/T2354-92 silica gel was used for silica gel flash chromatography. Visualization of the developed chromatogram was performed by ultraviolet absorbance (254 nm) or appropriate stains. ^1^HNMR data were recorded on Bruker nuclear resonance spectrometers (300, 400 or 500 MHz) unless specified otherwise. Chemical shifts (*δ*) in p.p.m. are reported as quoted relative to the residual signals of chloroform (^1^H 7.26 p.p.m. or ^13^C 77.16 p.p.m.). Multiplicities are described as: s (singlet), bs (broad singlet), d (doublet), t (triplet), q (quartet), m (multiplet); and coupling constants (*J*) are reported in Hertz (Hz). ^13^C NMR spectra were recorded on Bruker spectrometers (75, 101 or 126 MHz) with total proton decoupling. HRMS (electrospray ionization) analysis was performed by The Analytical Instrumentation Center at Peking University, Shenzhen Graduate School and (HRMS) data were reported with ion mass/charge (*m/z*) ratios as values in atomic mass units. ^1^H NMR, ^13^C NMR and HRMS are provided for all compounds; see Supplementary Figs 1–102. For ORTEP structures of **3in**, **4a**, **5a**, **6a**, see Supplementary Figs 103–106. See Supplementary Methods for the characterization data for all compounds. See Supplementary Data sets 1, 2, 3, 4 for X-ray CIF files of compounds **3in**, **4a**, **5a**, **6a** (CCDC 1028720, 1030998, 1031000, 1031001). See Supplementary Data set 5 for a list of structurally novel compounds.

*General procedure for the synthesis of **3***. Aldehyde **1** (0.1 mmol, 1.0 equiv.) was dissolved in DCM (1.0 ml, 0.1 M) in an oven-dried 8-ml vial equipped with a magnetic stir bar and a rubber septum. Secondary amine **2** (0.1 mmol, 1.0 equiv.) was added and the reaction mixture was stirred at 40 °C for 2 h. Solvent was removed under *vacuo* and the residue was purified by flash neutral Al_2_O_3_ column chromatography (eluent: petroleum ether/ethyl ether=100: 1) to afford the desired 1-silyloxy-3-aminobutadiene **3**.

*General procedure for the synthesis of **4***. Diene **3** (0.05 mmol, 1.0 equiv.) was dissolved in DCM (1 ml). The solution was stirred at −20 °C for 5 min. *N*-bromosuccinimide (0.2 mmol, 2.0 equiv.) was added and the reaction mixture was stirred at −20 °C for 2 h. Solvent was evaporated and the residue was purified by silica gel column chromatography (Et_2_O: petroleum ether=1: 10) to afford the desired product **4**.

*General procedure for the synthesis of **5***. Diene **3** (0.05 mmol, 1.0 equiv.) was dissolved in DCM (1 ml). The solution was stirred at −20 °C for 5 min. A solution of trifluoroacetic acid (0.11 mmol, 1.1 equiv.) in DCM (0.2 ml) was added dropwise and the reaction mixture was stirred at −20 °C overnight. The reaction was then warmed to room temperature and stirred for another 1 h. Solvent was evaporated and the residue was purified by silica gel column chromatography (ethyl acetate: petroleum ether=1: 4) to afford the desired product **5**.

*General procedure for the synthesis of **6***. To a solution of diene **3** (0.05 mmol, 1.0 equiv.) in DCM (1 ml) was added the Togni's reagent (0.05 mmol, 1.0 equiv.). The reaction mixture was stirred at 50 °C for 24 h. Solvent was evaporated and the residue was purified by silica gel column chromatography (ethyl acetate: petroleum ether=1: 4) to afford the desired product **6**.

*Procedure for the synthesis of **7a***. To a solution of **3aa** (113 mg, 0.2 mmol) in DCE (2 ml) was added 4-phenyl-1-tosyl-1*H*-1,2,3-triazole (30 mg, 0.1 mmol) and Rh_2_(OAc)_4_ (4.4 mg, 0.01 mmol). The reaction mixture was stirred at 100 °C for 48 h. Solvent was evaporated and the residue was purified by silica gel column chromatography (ethyl acetate: petroleum ether=1: 8) to afford the desired product **7a** as a yellow oil.

*Procedure for the synthesis of **8a***. To the solution of diene **3aa** (56 mg, 0.1 mmol), *rac*-*trans*-2,5-diphenylpyrrolidine (4.5 mg, 0.02 mmol) and 4-nitrobenzoic acid (3.3 mg, 0.02 mmol) in DCM (2 ml) was added acrolein (10 μl, 0.15 mmol). The reaction mixture was stirred at room temperature for 2 h. Solvent was evaporated and the residue was purified by silica gel column chromatography (ethyl acetate: petroleum ether=1: 25) to afford the desired product **8a** as a light yellow oil.

## Author contributions

Y.H. conceived and directed the project. X.L. and S.P. performed the experiments. L.L. solved the X-ray structures. Y.H. and X.L. analysed the results. Y.H. wrote the manuscript with the assistance of X.L.

## Additional information

**Accession codes**: The X-ray crystallographic coordinates for structures reported in this study have been deposited at the Cambridge Crystallographic Data Centre (CCDC), under deposition numbers CCDC 1028720, 1030998, 1031000, 1031001. These data can be obtained free of charge from The Cambridge Crystallographic Data Centre via www.ccdc.cam.ac.uk/data_request/cif.

**How to cite this article:** Li, X. *et al*. Synthesis of tetrasubstituted 1-silyloxy-3-aminobutadienes and chemistry beyond Diels–Alder reactions. *Nat. Commun*. 6:6913 doi: 10.1038/ncomms7913 (2015).

## Supplementary Material

Supplementary Figures and Supplementary Methods Supplementary Figures 1-106 and Supplementary Methods

Supplementary Data 1 X-ray CIF files of compounds **3in**. CCDC 1028720

Supplementary Data 2 X-ray CIF files of compounds **4a**. CCDC 1030998

Supplementary Data 3 X-ray CIF files of compounds **5a**. CCDC 1031000

Supplementary Data 4 X-ray CIF files of compounds **6a**. CCDC 1031001

Supplementary Data 5 List of structurally novel compounds

## Figures and Tables

**Table 1:** Substrate scope of allenyl aldehyde.

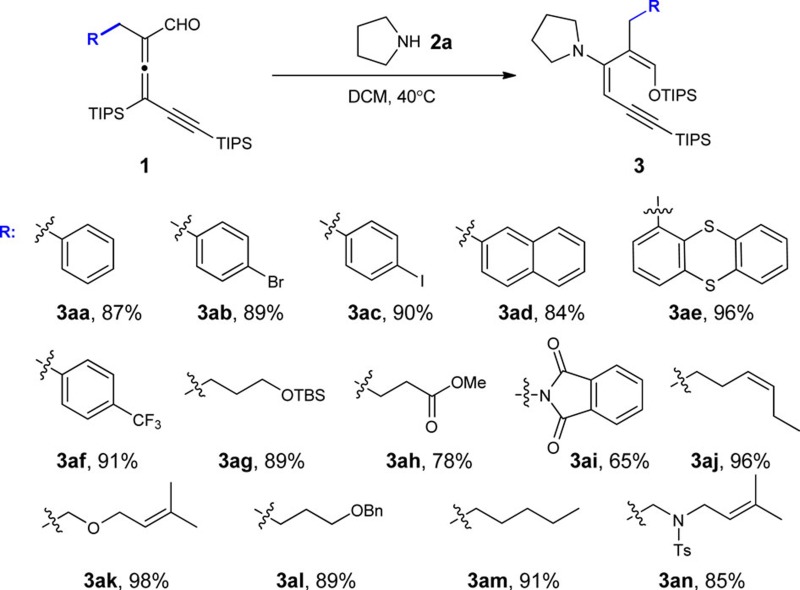

(a) Reactions were conducted using 0.1 mmol allenyl aldehyde and 0.1 mmol pyrrolidine in 1 ml dichloromethane for 2 h. Isolated yield. (b) Ts: tosyl.

**Table 2:** Substrate scope of amine.

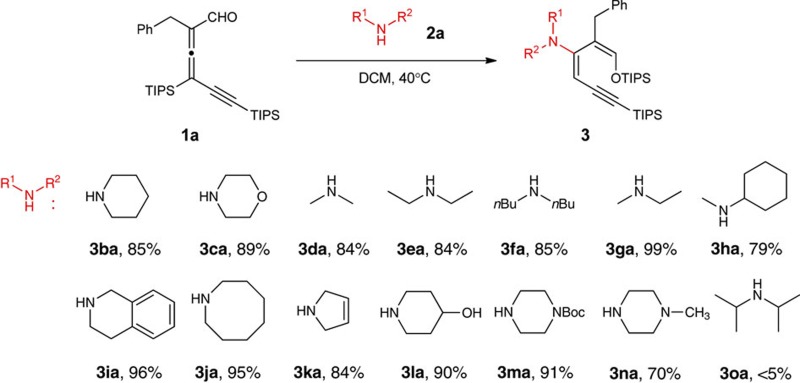

(a) Reactions were conducted with 0.1 mmol allenyl aldehydes and 0.1 mmol pyrrolidine in 1 ml solvent for 2 h. Isolated yield. (b) For products **3fa**, **3ha** and **3na**, 2.0 equiv. amine was used and the reaction time was 20 h. (c) Boc: *t*-Butyloxycarbonyl.
