# Supplementary material for: Synthesis of tetrasubstituted 1-silyloxy-3-aminobutadienes and chemistry beyond Diels–Alder reactions
Source: Nat Commun. 2015 Apr 21;6:6913. doi: 10.1038/ncomms7913 (PMC4411301; doi:10.1038/ncomms7913)
Supplement: Supplementary Figures and Supplementary Methods — Supplementary Figures 1-106 and Supplementary Methods [file ncomms7913-s1.pdf]

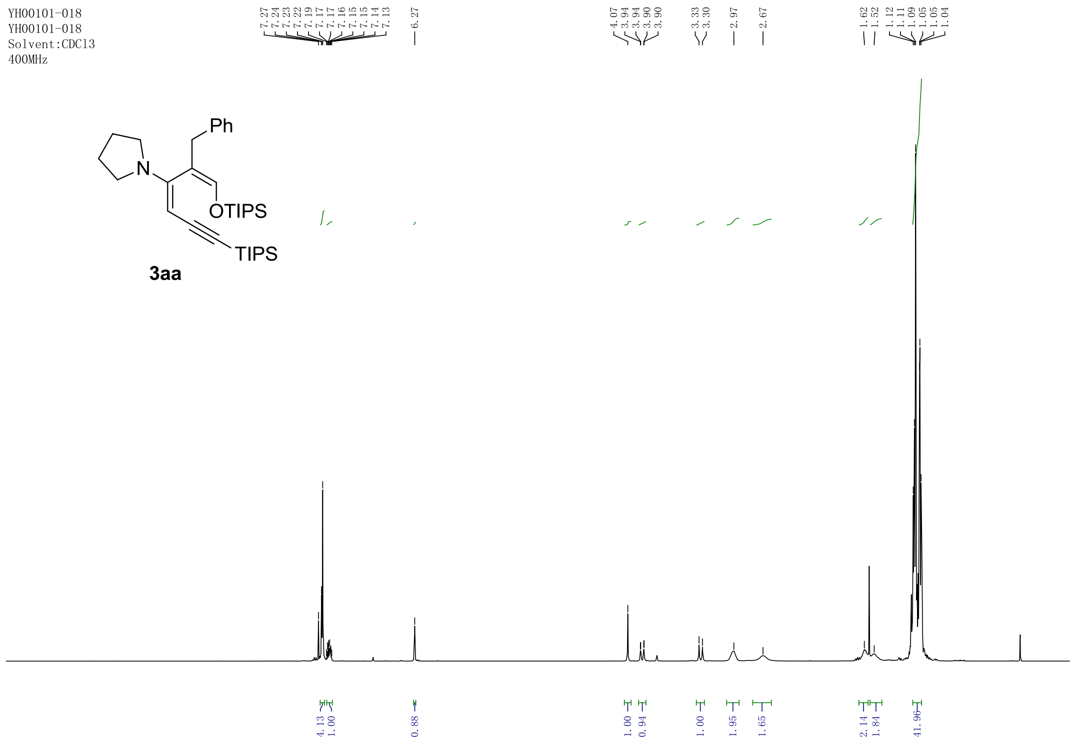

Supplementary Figure 1. ^{1}H NMR (400MHz, $CDCl_3$) spectrum for 3aa

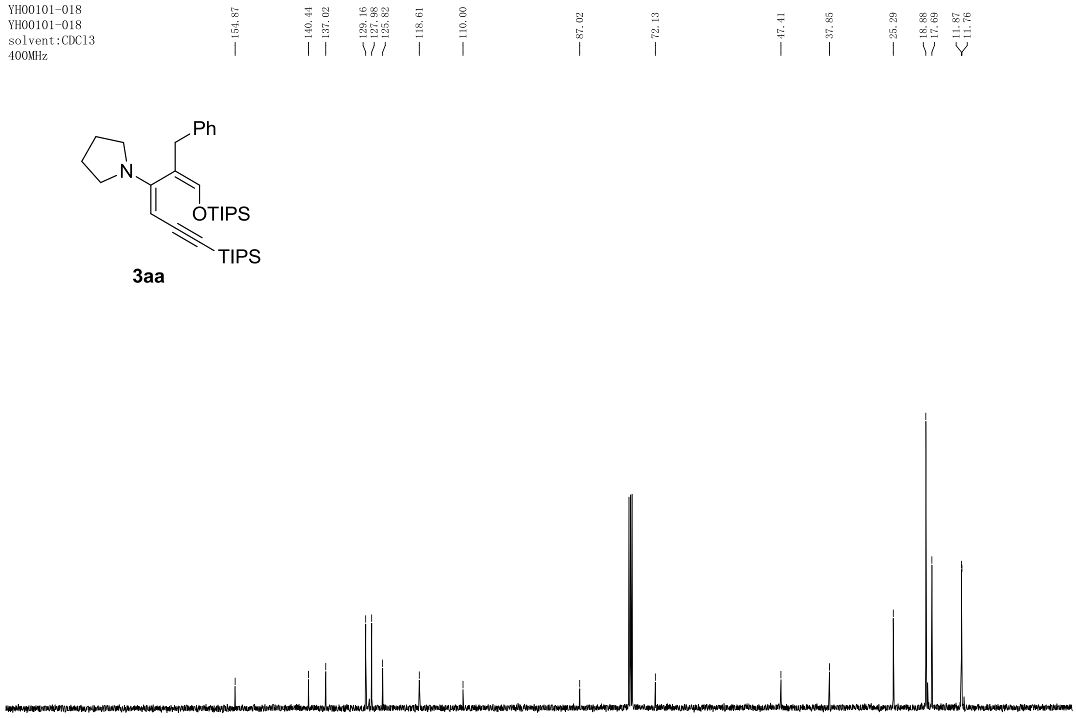

Supplementary Figure 2. ^{13}C NMR (101MHz, $CDCl_3$) spectrum for 3aa

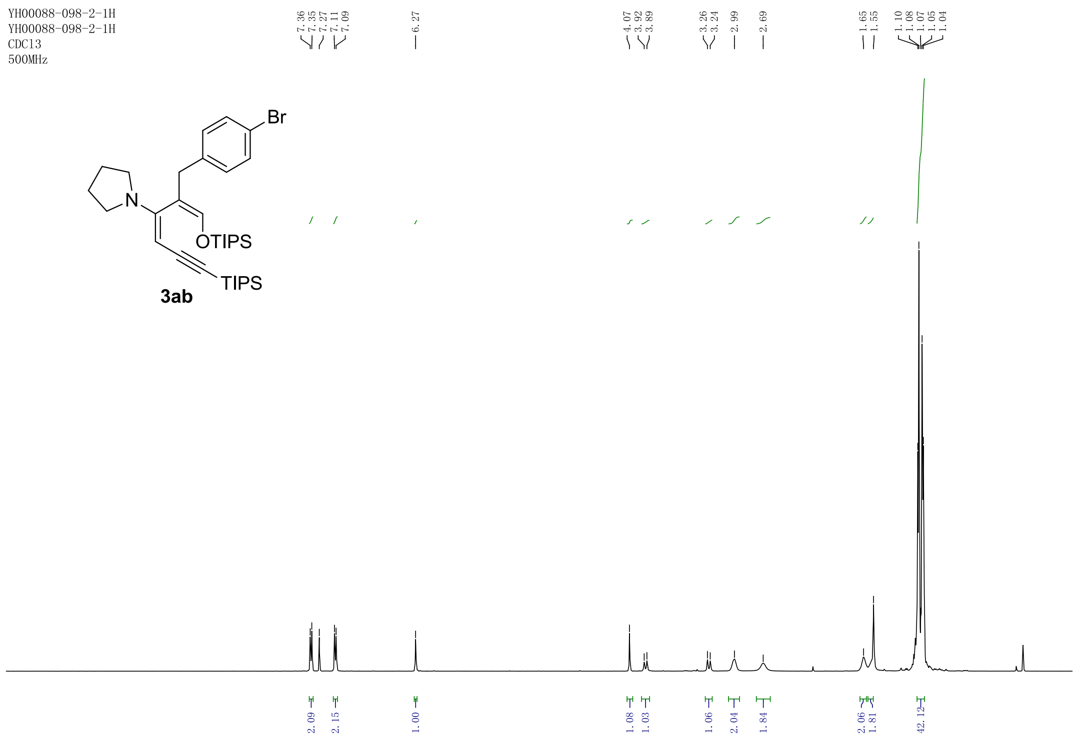

Supplementary Figure 3. ^{1}H NMR (500MHz, CDCl₃) spectrum for 3ab

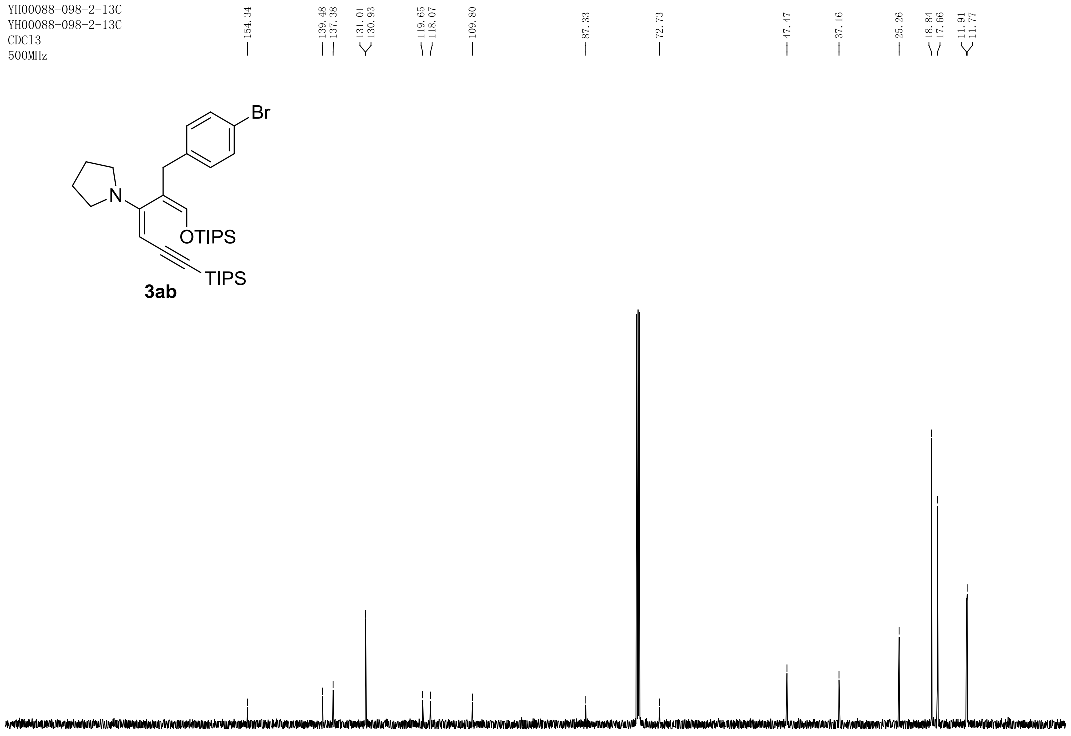

Supplementary Figure 4. ^{13}C NMR (126MHz, CDCl₃) spectrum for 3ab

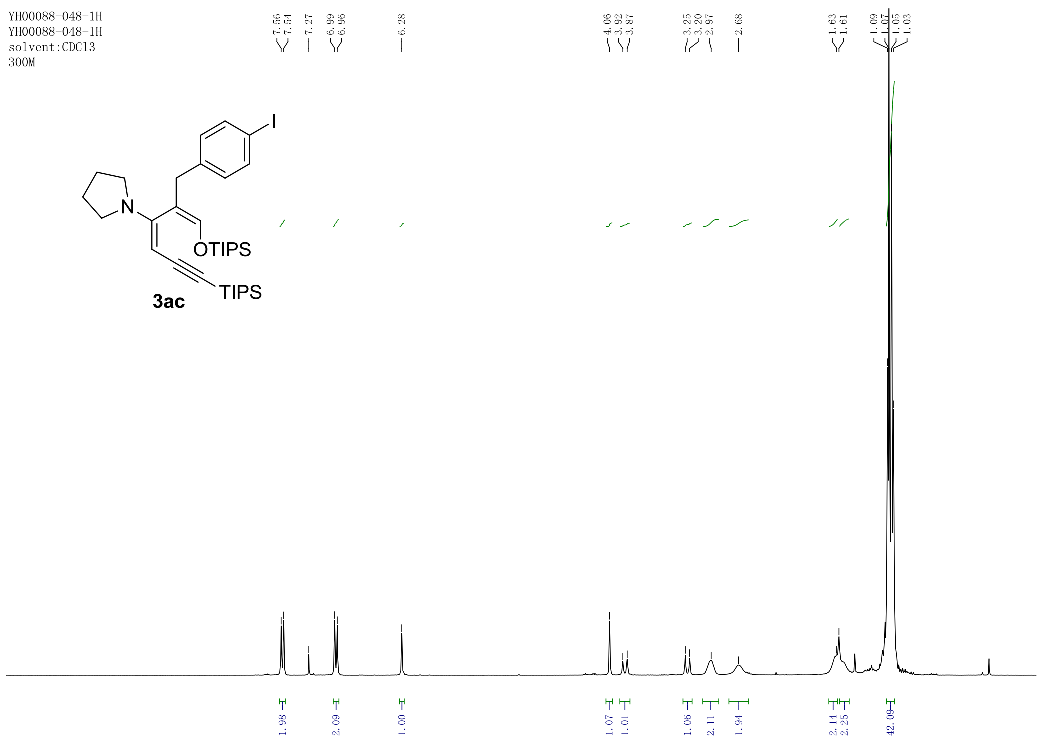

Supplementary Figure 5. ^{1}H NMR (300MHz, $CDCl_3$) spectrum for 3ac

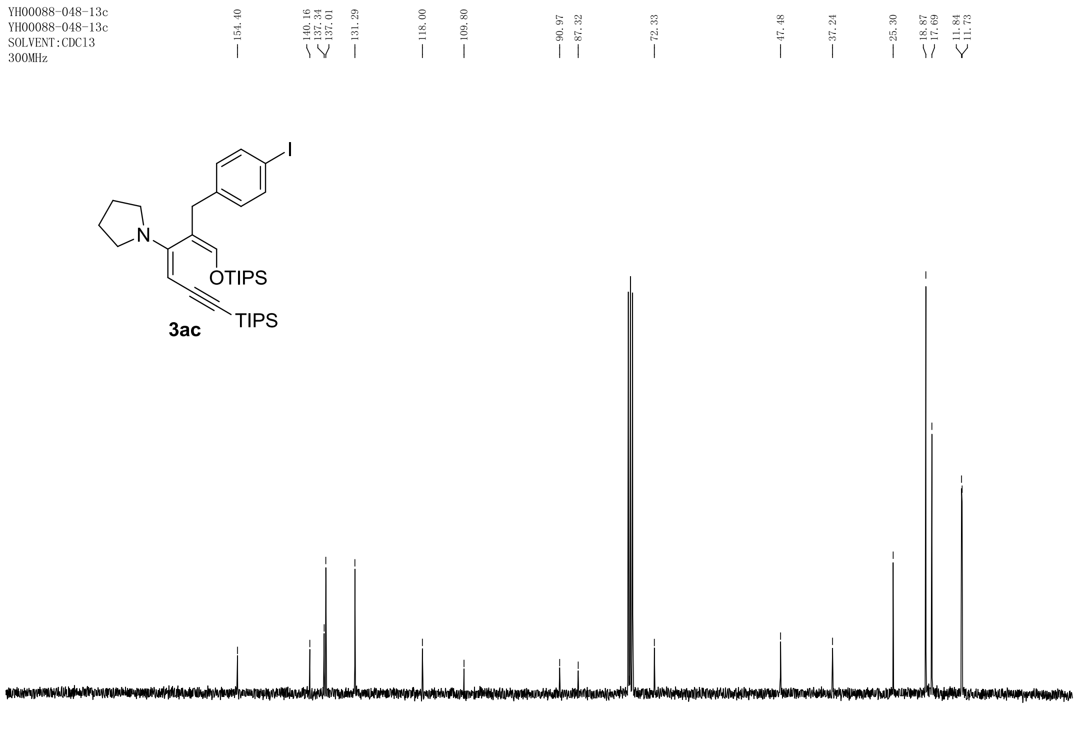

Supplementary Figure 6. ^{13}C NMR (75MHz, $CDCl_3$) spectrum for 3ac

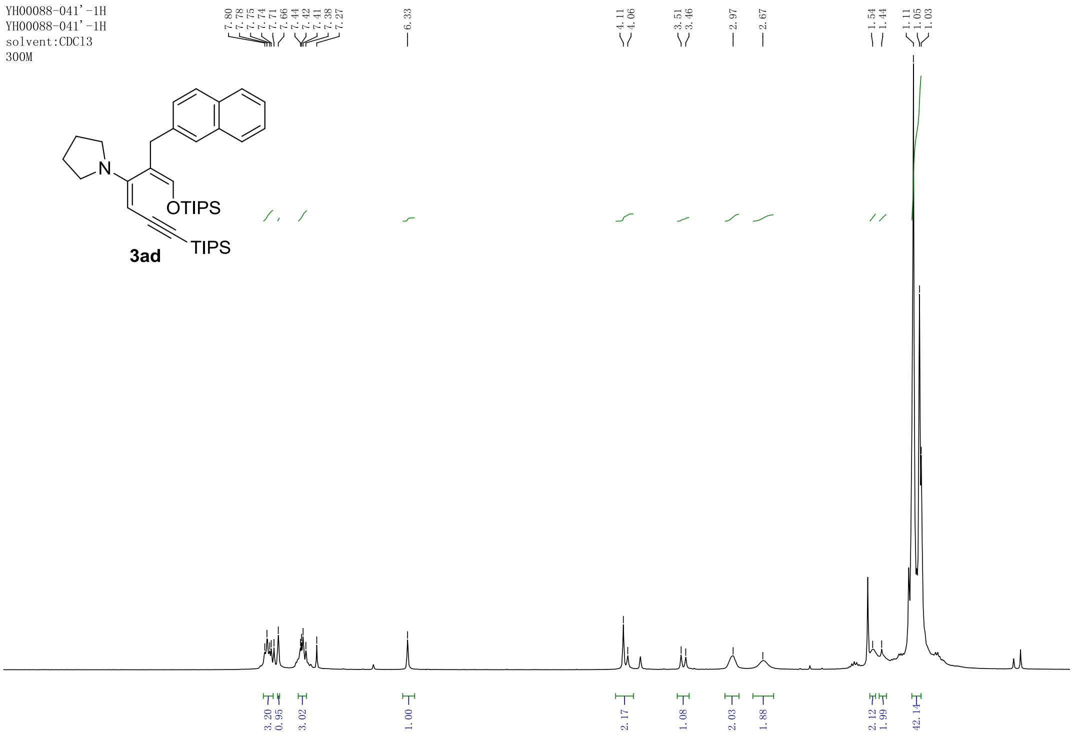

Supplementary Figure 7. ^{1}H NMR (300MHz, $CDCl_3$) spectrum for 3ad

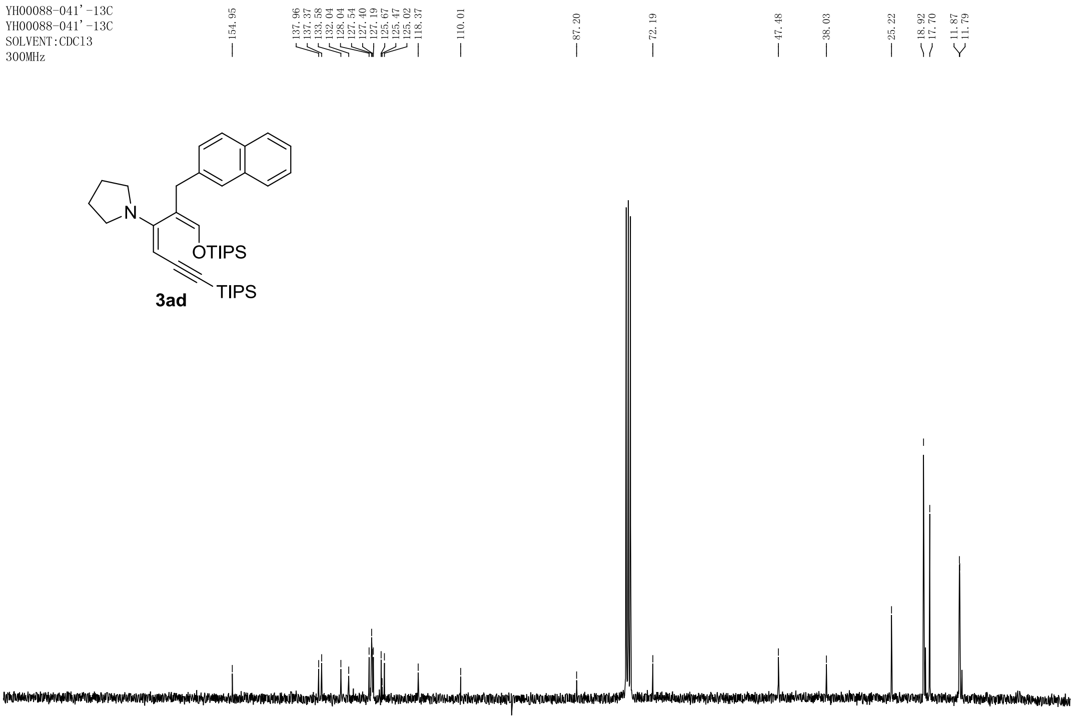

Supplementary Figure 8. ^{13}C NMR (75MHz, $CDCl_3$) spectrum for 3ad

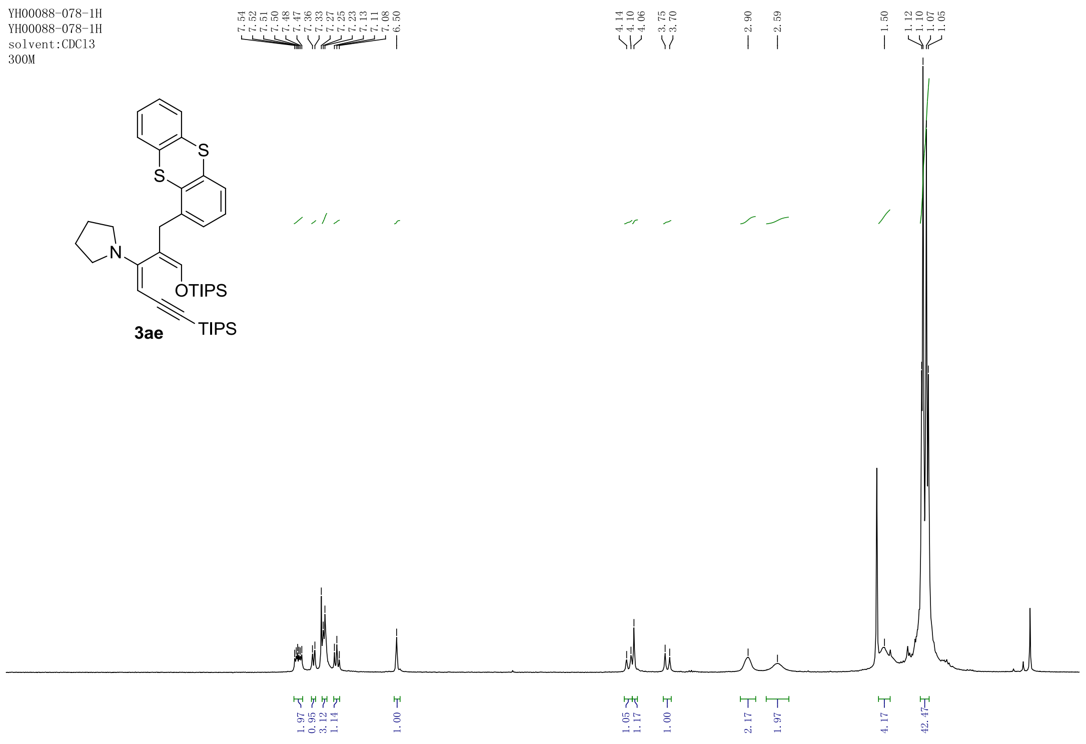

Supplementary Figure 9. ^{1}H NMR (300MHz, $CDCl_3$) spectrum for 3ae

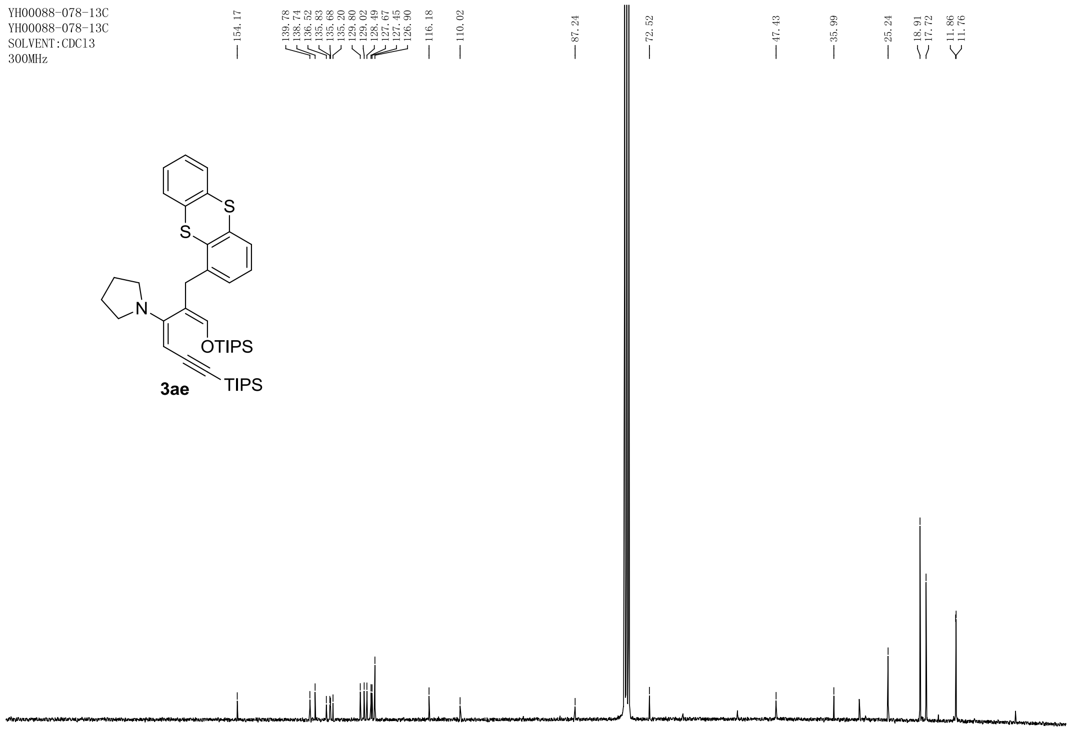

Supplementary Figure 10. ^{13}C NMR (75MHz, $CDCl_3$) spectrum for 3ae

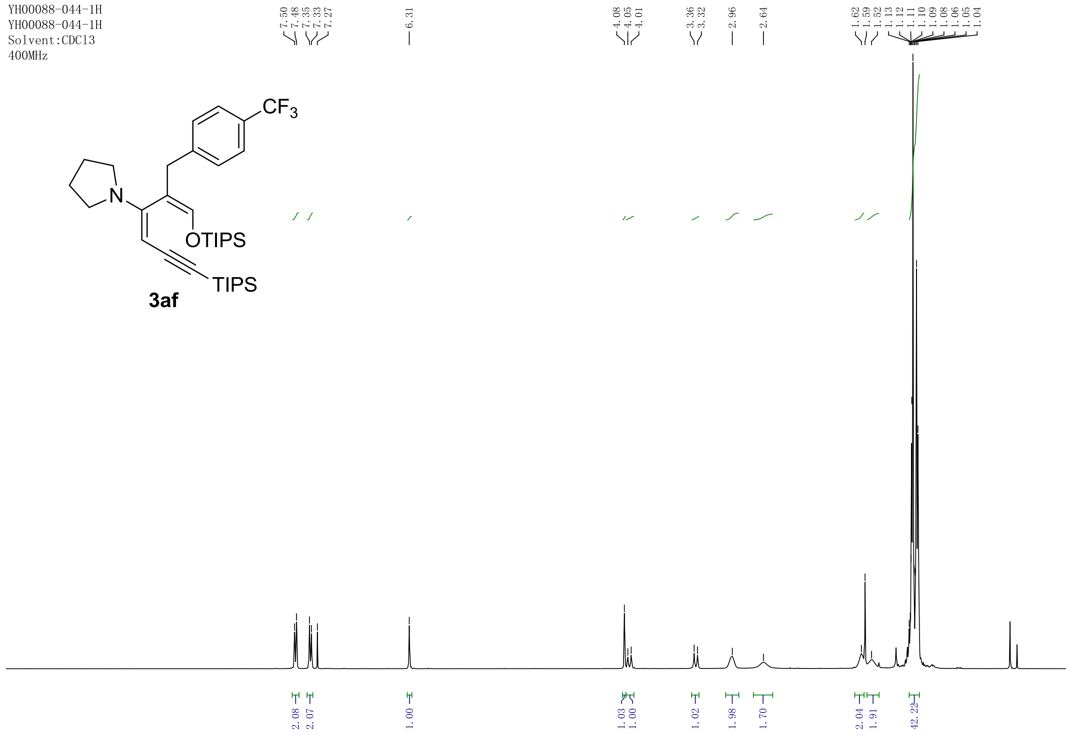

Supplementary Figure 11. ^{1}H NMR (400MHz, CDCl$_3$) spectrum for 3af

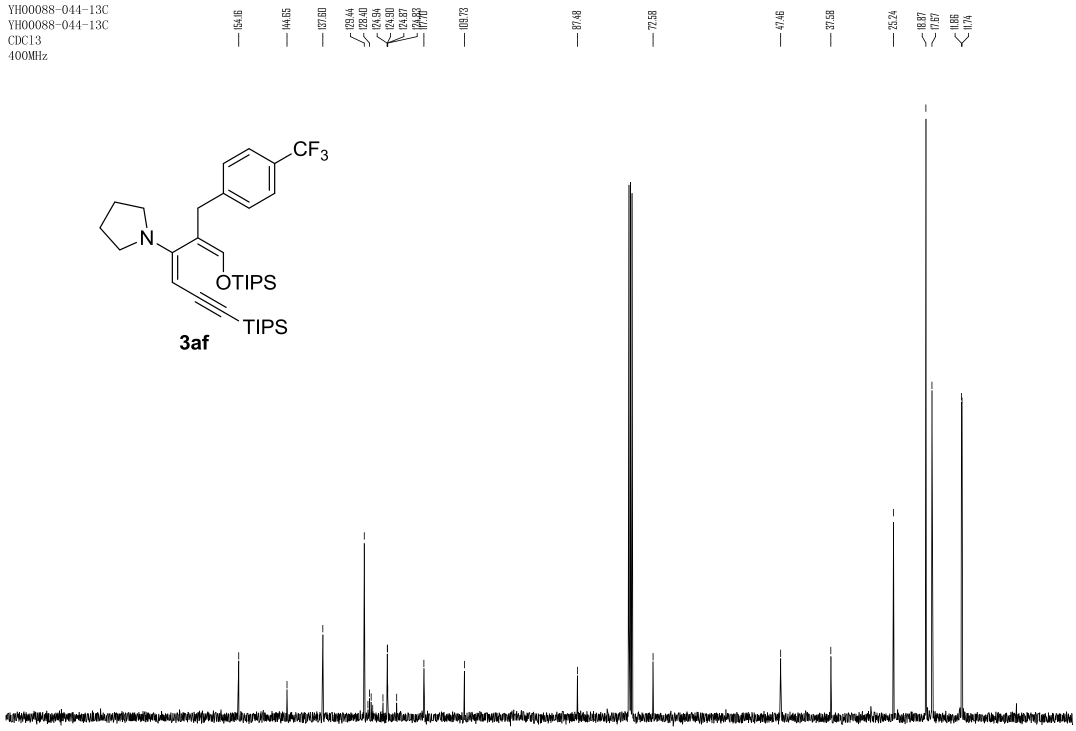

Supplementary Figure 12. ^{13}C NMR (101MHz, CDCl$_3$) spectrum for 3af

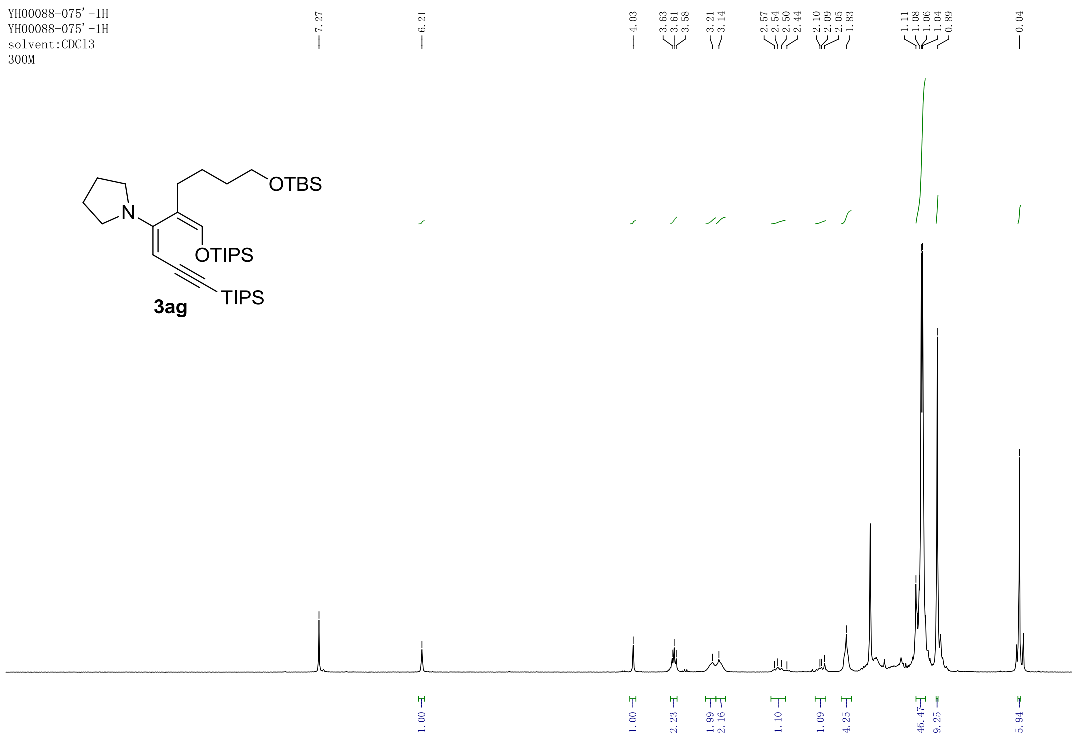

Supplementary Figure 13. ^{1}H NMR (300MHz, $CDCl_3$) spectrum for 3ag

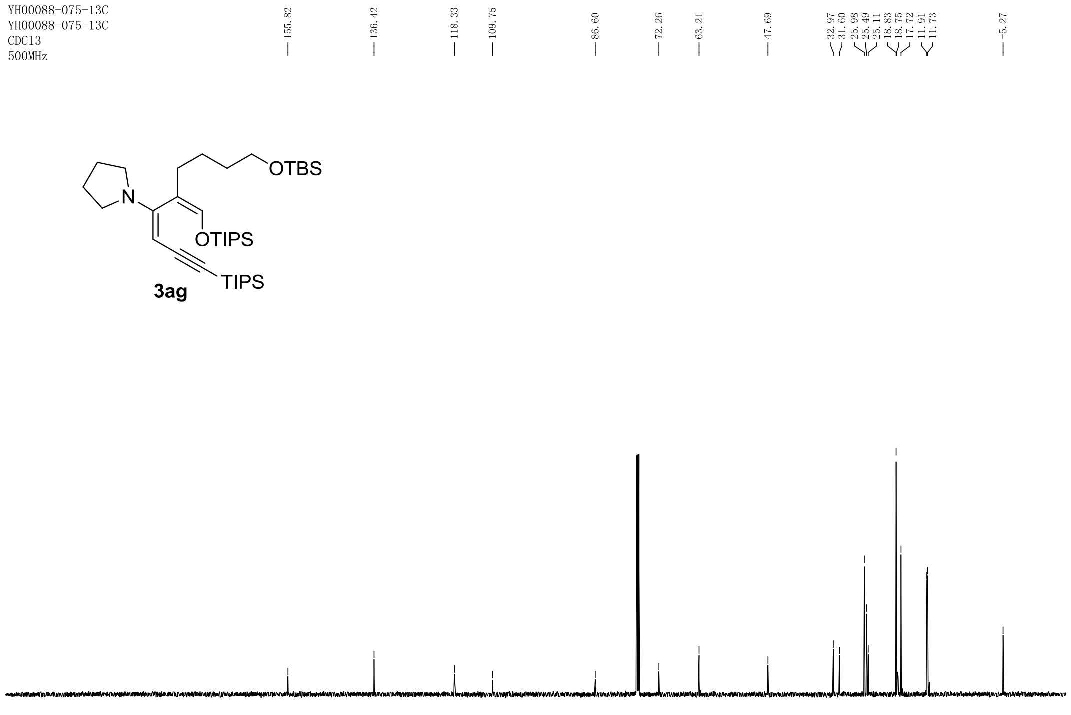

Supplementary Figure 14. ^{13}C NMR (126MHz, $CDCl_3$) spectrum for 3ag

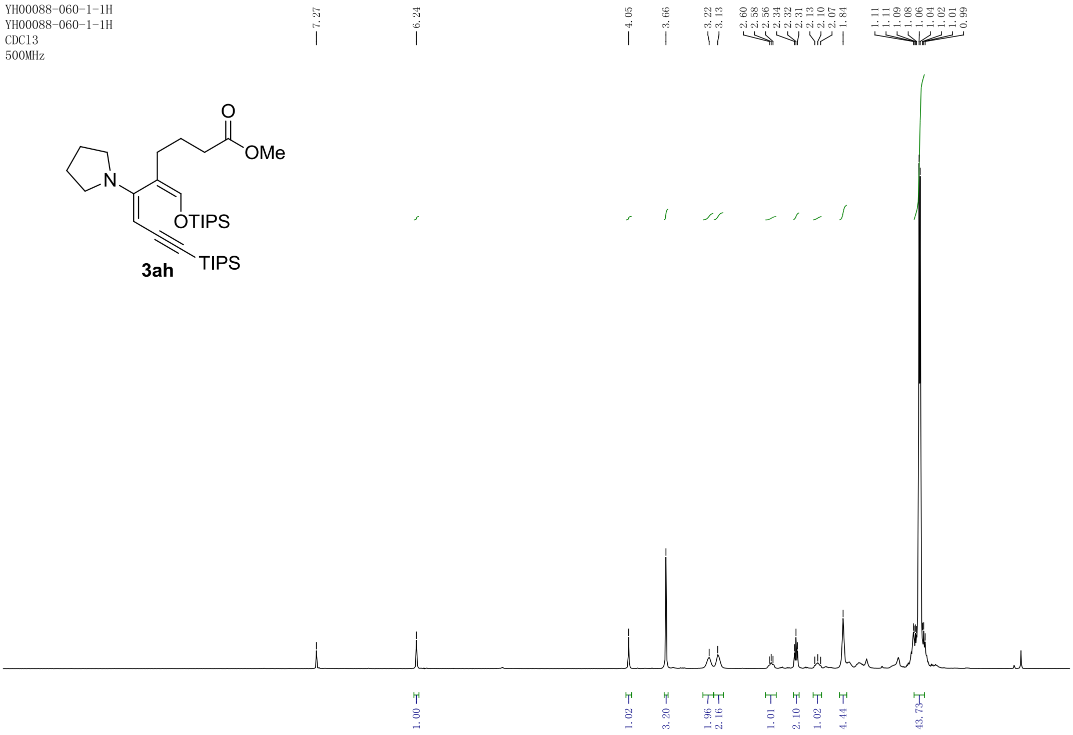

Supplementary Figure 15. ^{1}H **NMR (500MHz,** $CDCl_3$**) spectrum for 3ah**

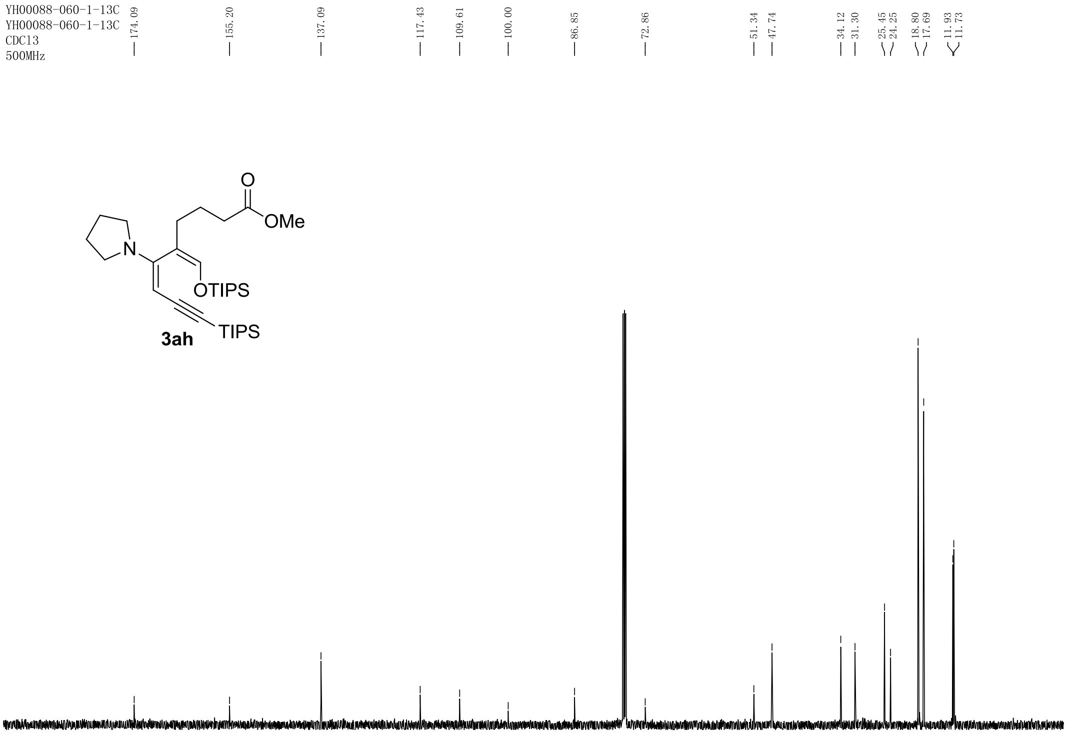

Supplementary Figure 16. ^{13}C **NMR (126MHz,** $CDCl_3$**) spectrum for 3ah**

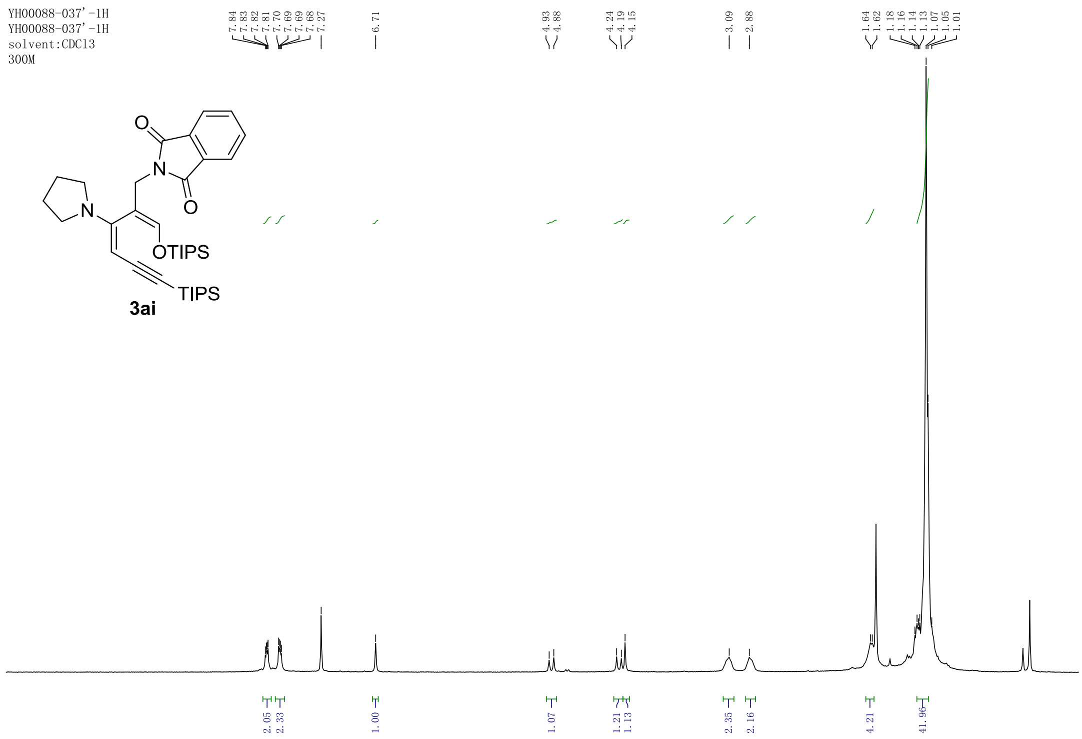

Supplementary Figure 17. ^{1}H NMR (300MHz, $CDCl_3$) spectrum for 3ai

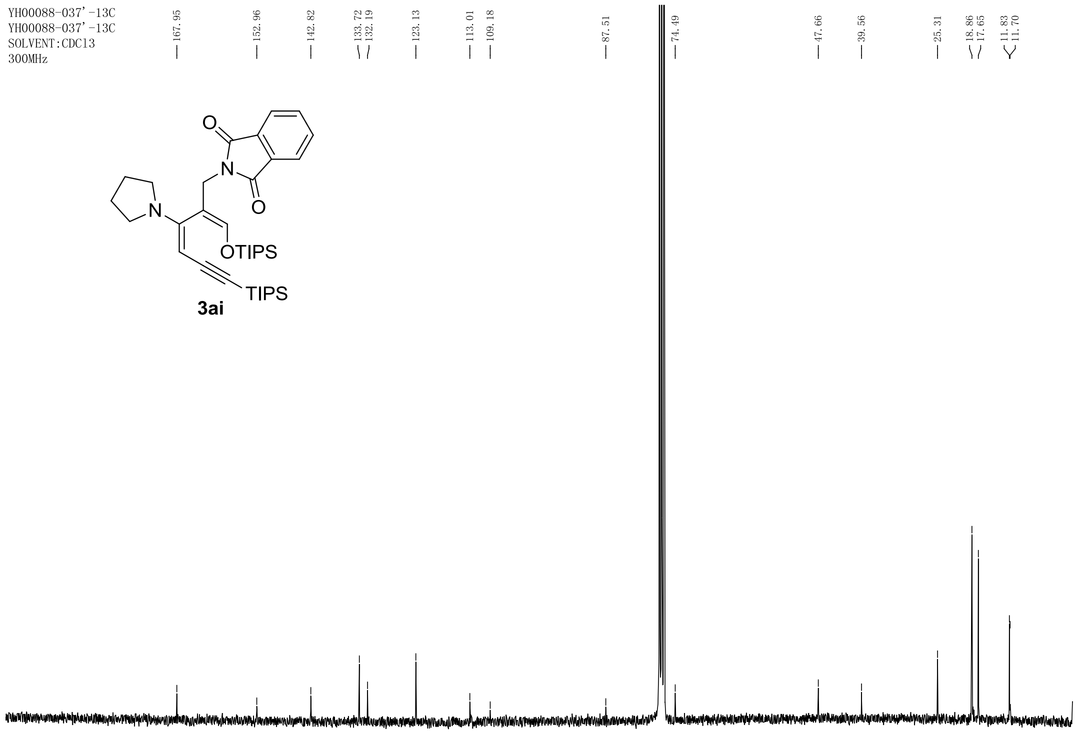

Supplementary Figure 18. ^{13}C NMR (75MHz, $CDCl_3$) spectrum for 3ai

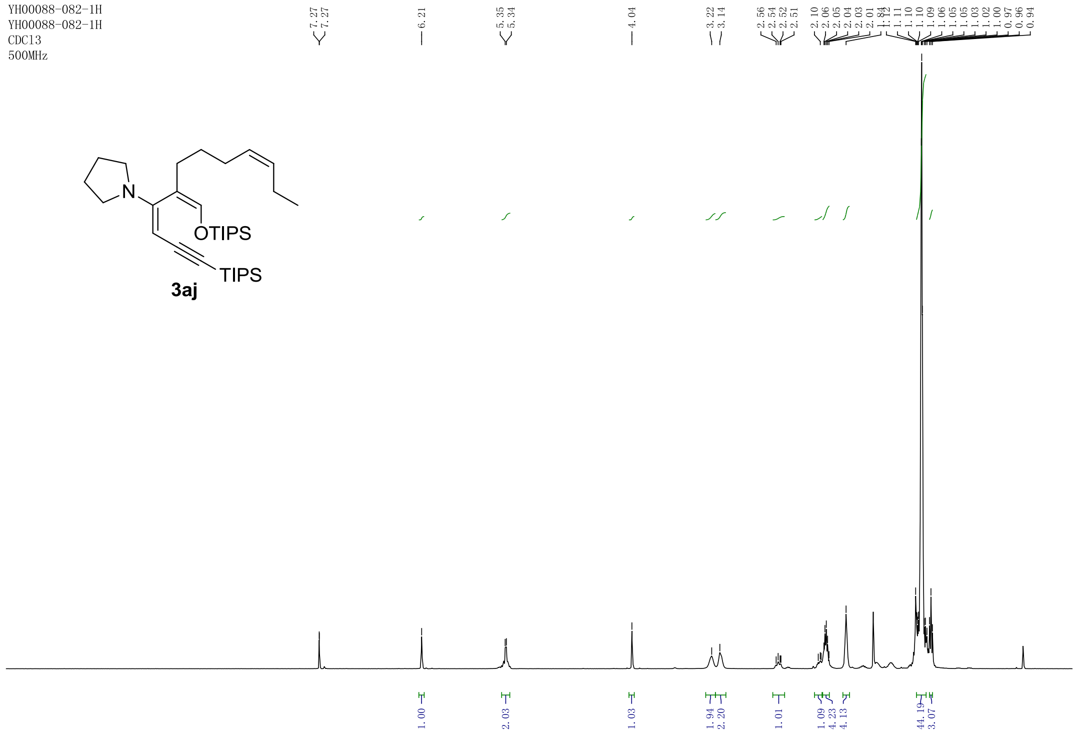

Supplementary Figure 19. ^{1}H NMR (500MHz, $CDCl_3$) spectrum for 3aj

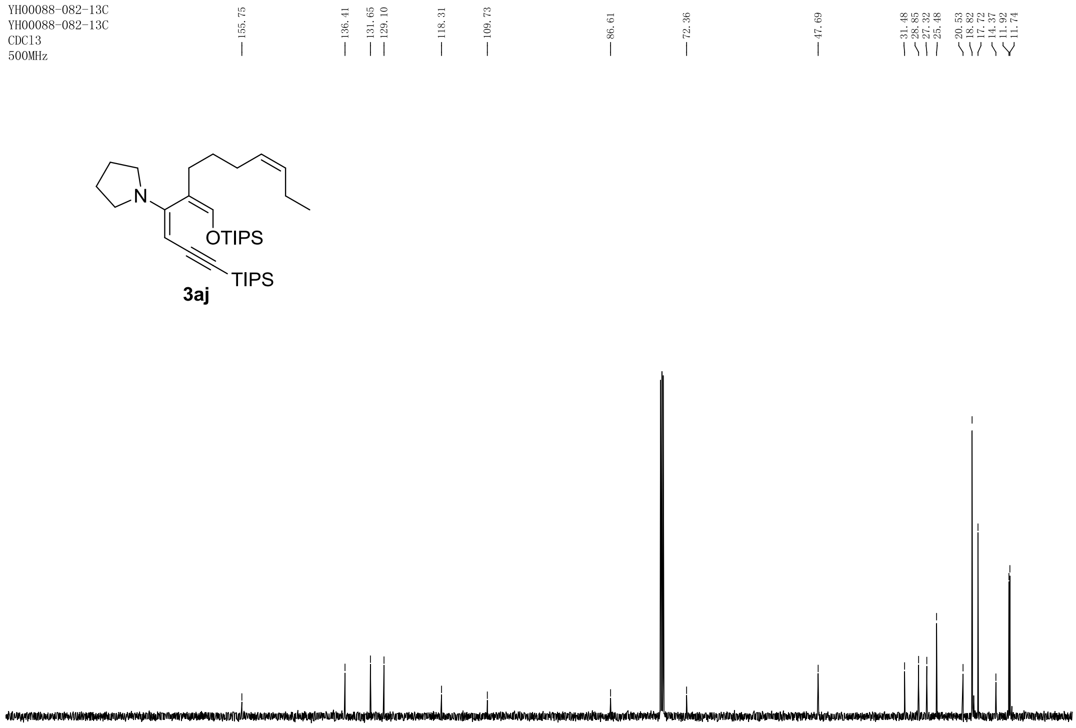

Supplementary Figure 20. ^{13}C NMR (126MHz, $CDCl_3$) spectrum for 3aj

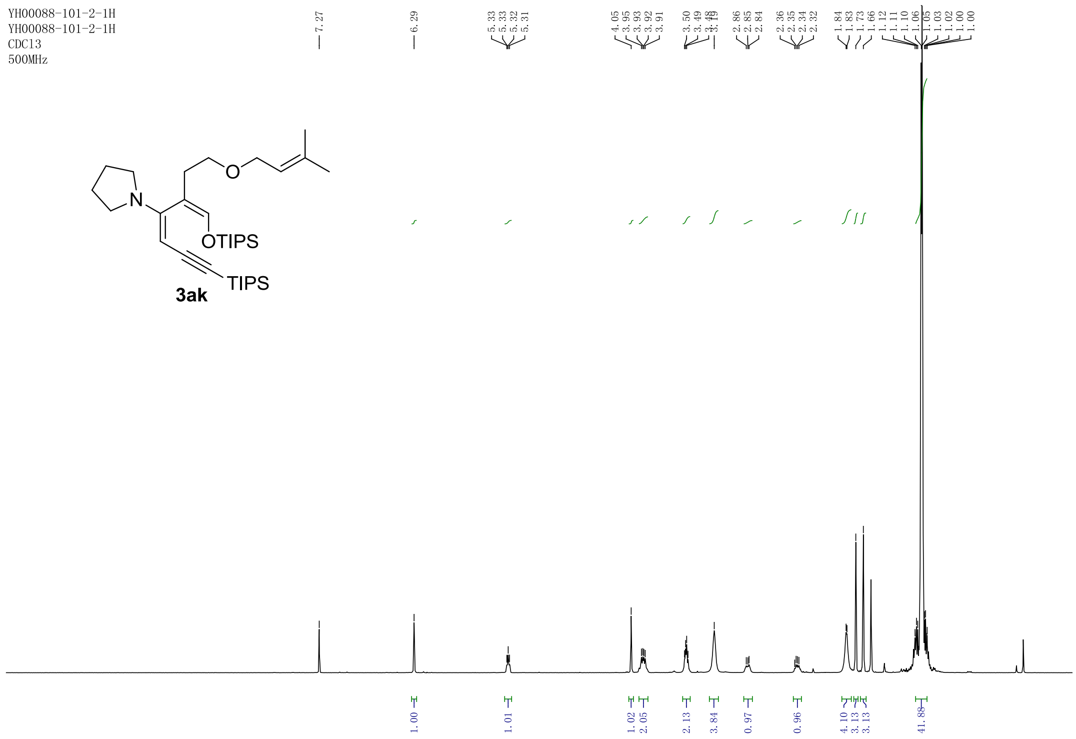

Supplementary Figure 21. ^{1}H NMR (500MHz, CDCl$_3$) spectrum for 3ak

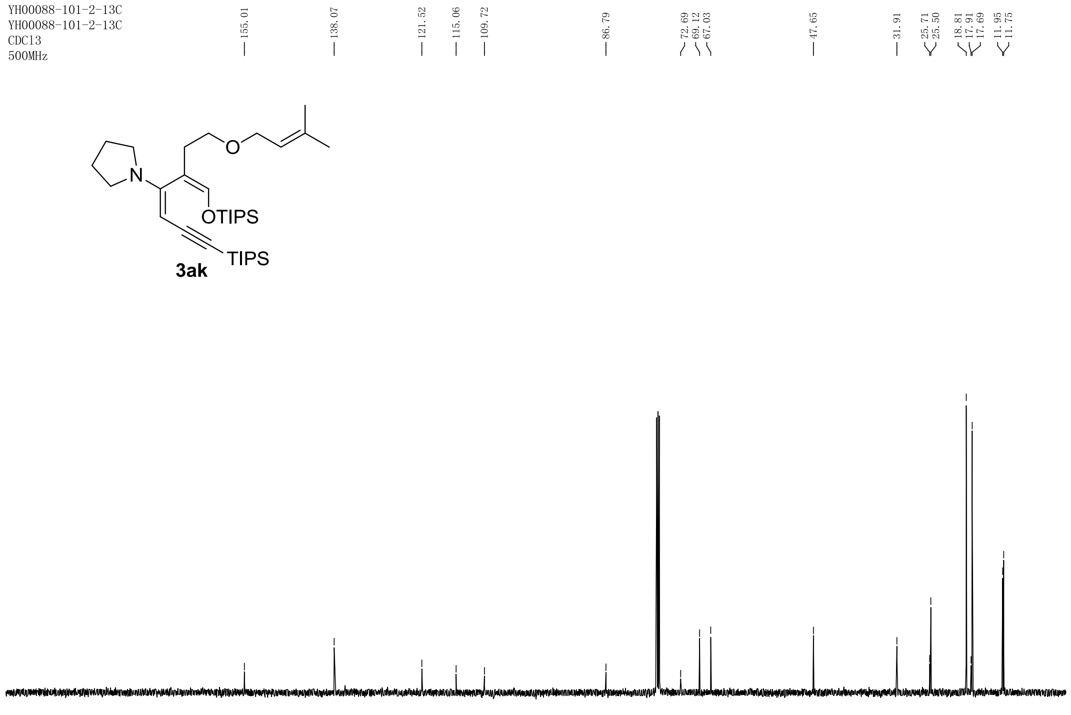

Supplementary Figure 22. ^{13}C NMR (126MHz, CDCl$_3$) spectrum for 3ak

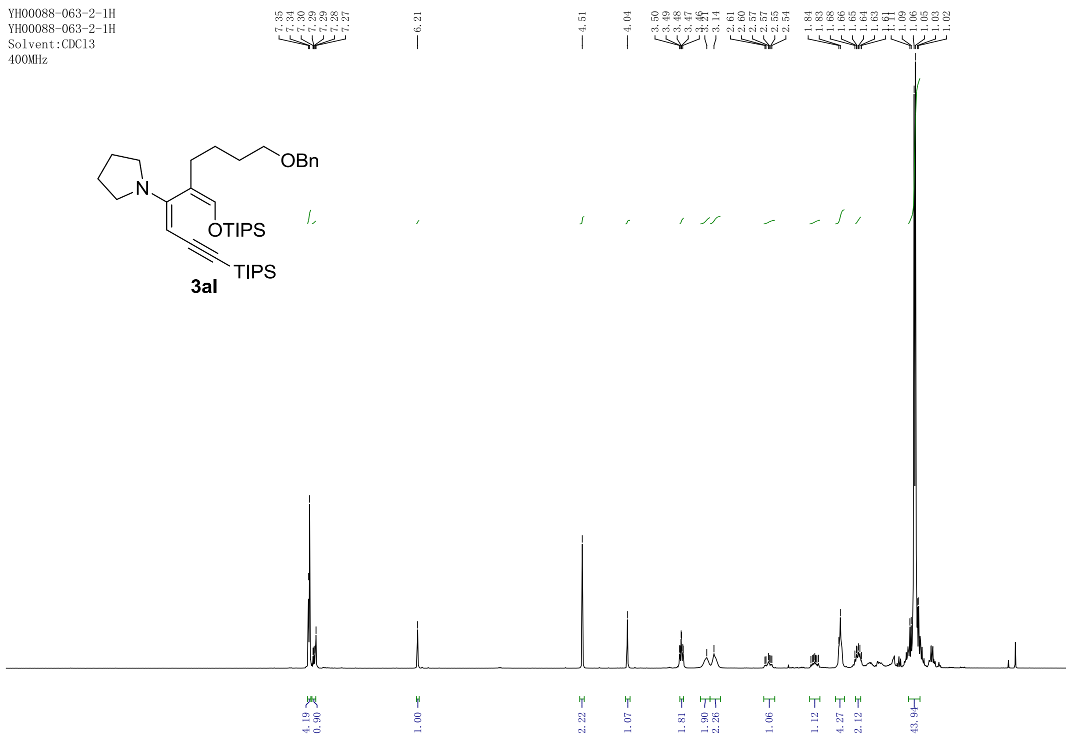

Supplementary Figure 23. ^{1}H NMR (400MHz, $CDCl_3$) spectrum for 3al

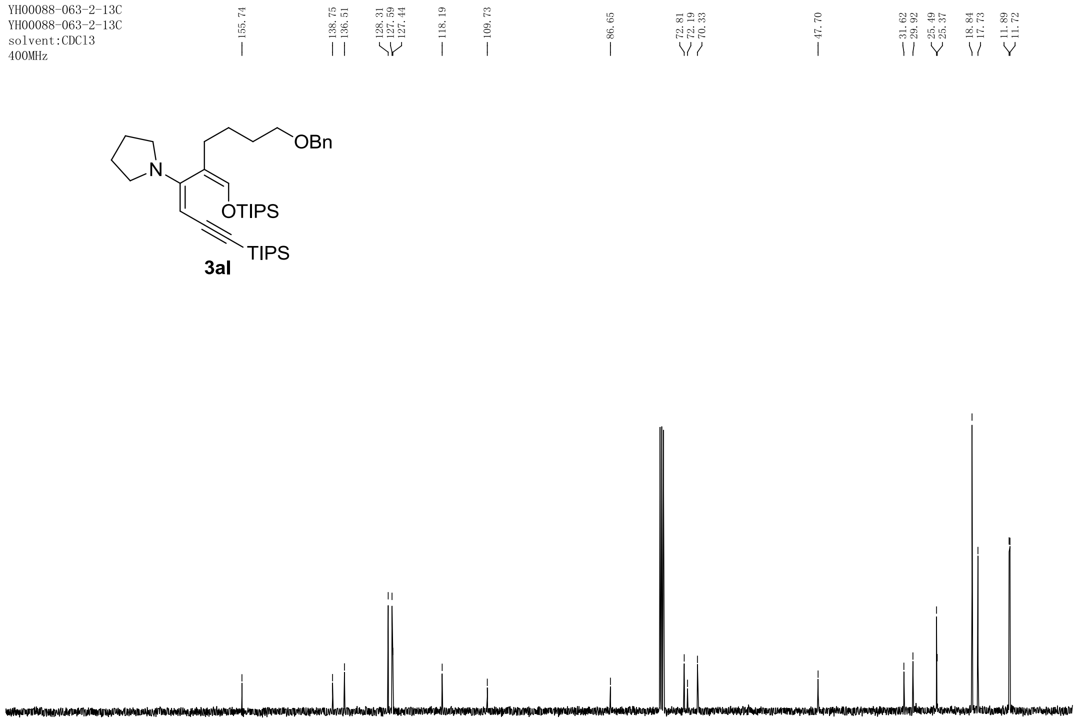

Supplementary Figure 24. ^{13}C NMR (101MHz, $CDCl_3$) spectrum for 3al

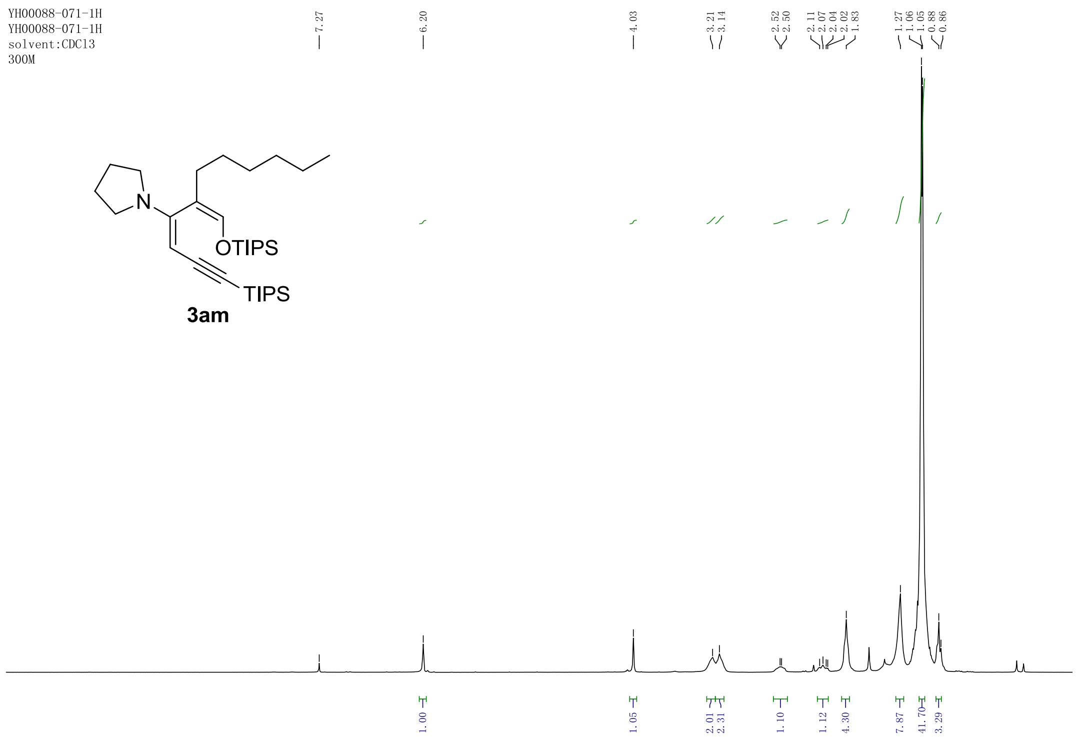

Supplementary Figure 25. ^{1}H NMR (300MHz, $CDCl_3$) spectrum for 3am

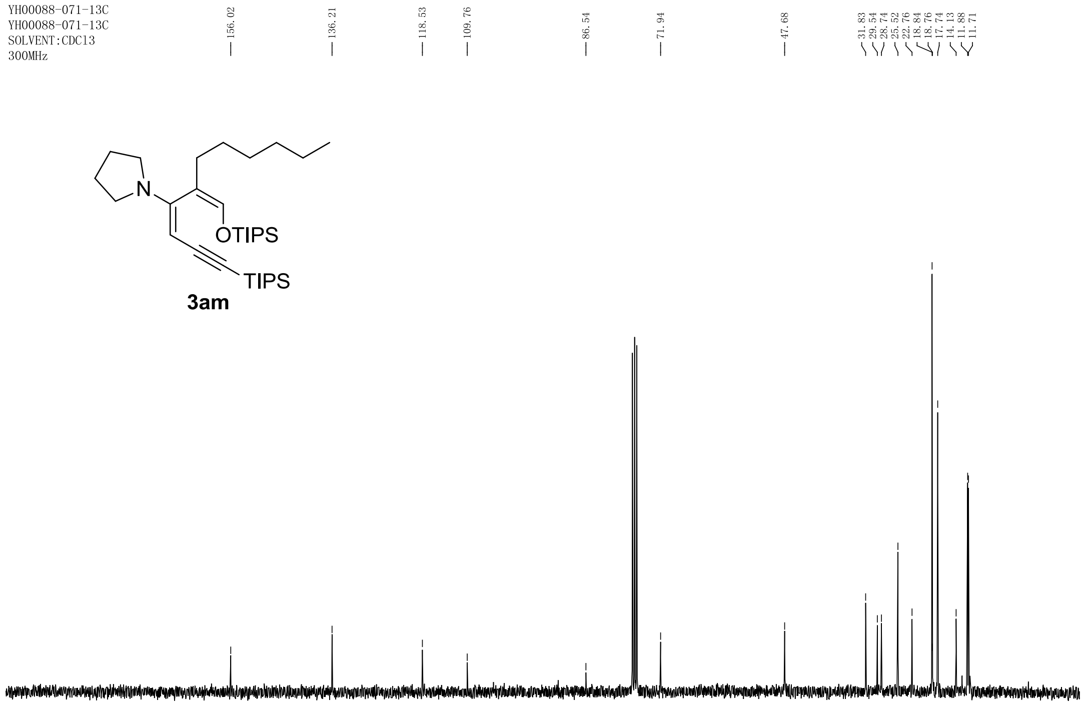

Supplementary Figure 26. ^{13}C NMR (75MHz, $CDCl_3$) spectrum for 3am

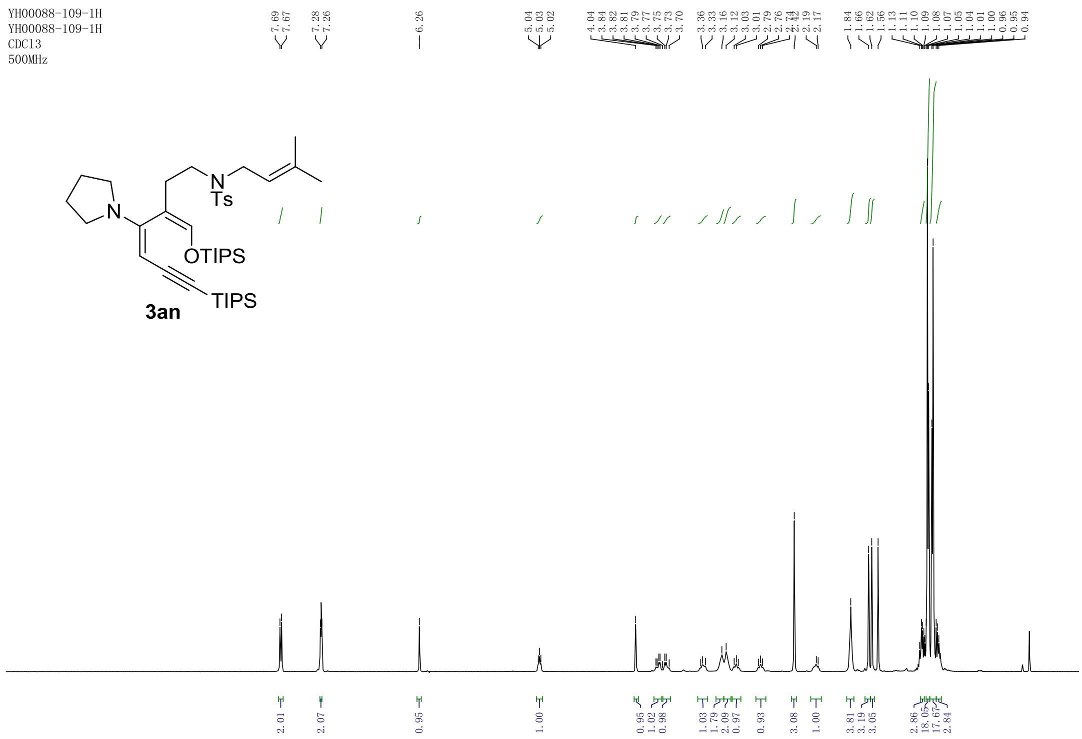

Supplementary Figure 27. ^{1}H NMR (500MHz, CDCl$_3$) spectrum for 3an

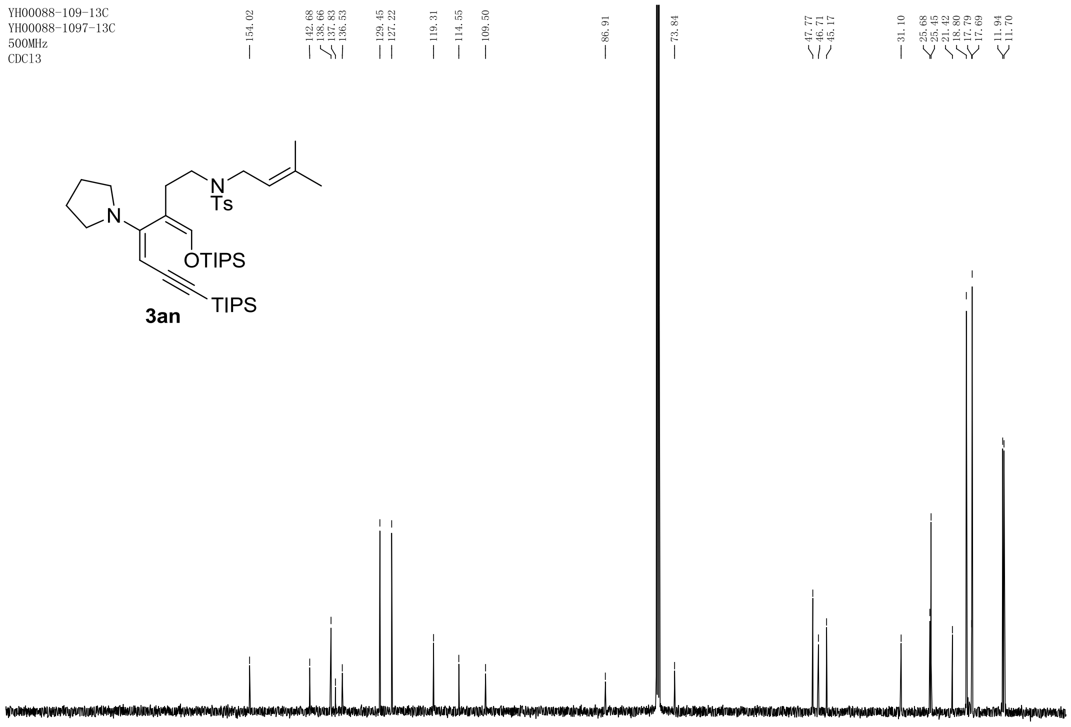

Supplementary Figure 28. ^{13}C NMR (126MHz, CDCl$_3$) spectrum for 3an

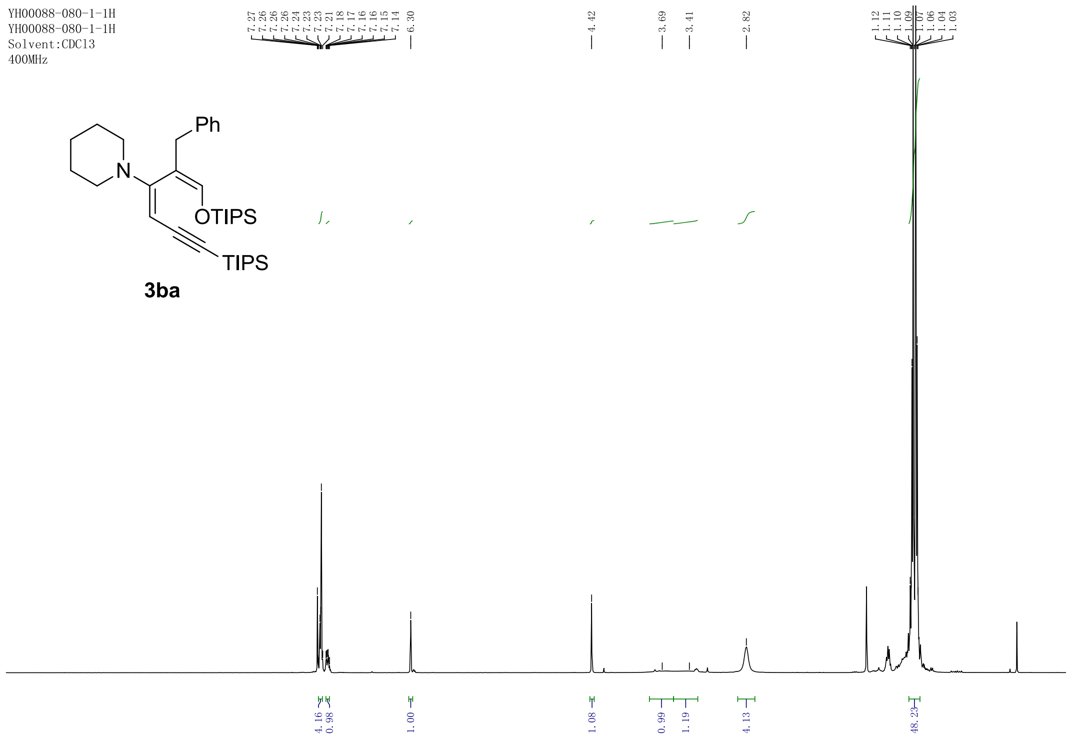

Supplementary Figure 29. ^{1}H NMR (400MHz, $CDCl_3$) spectrum for 3ba

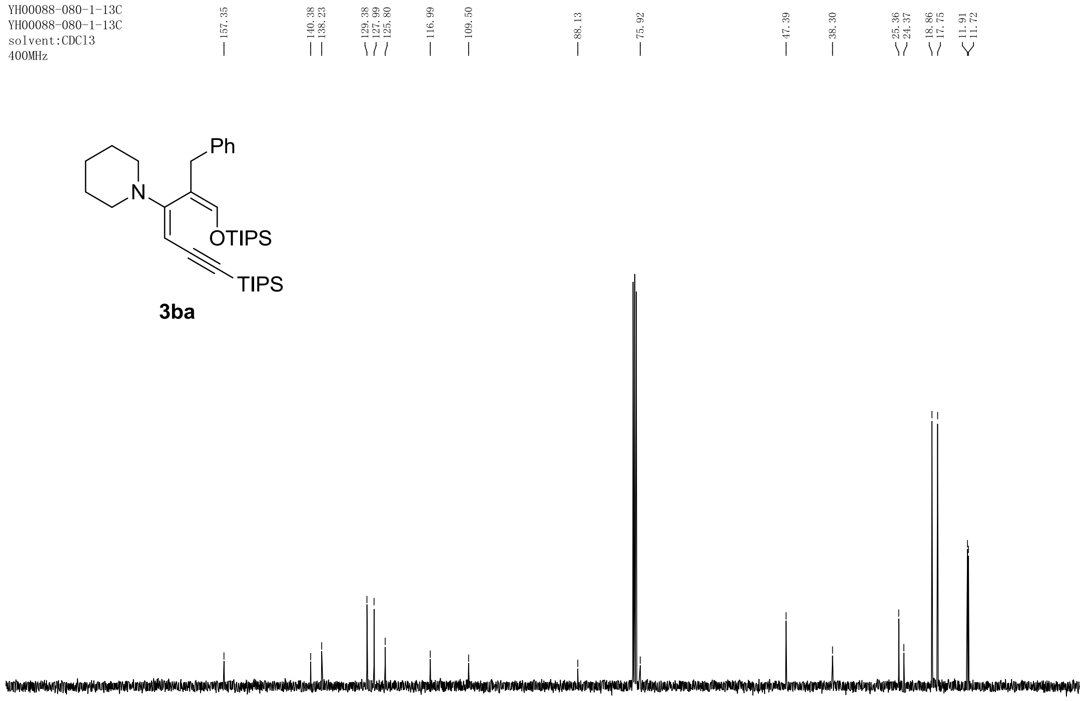

Supplementary Figure 30. ^{13}C NMR (101MHz, $CDCl_3$) spectrum for 3ba

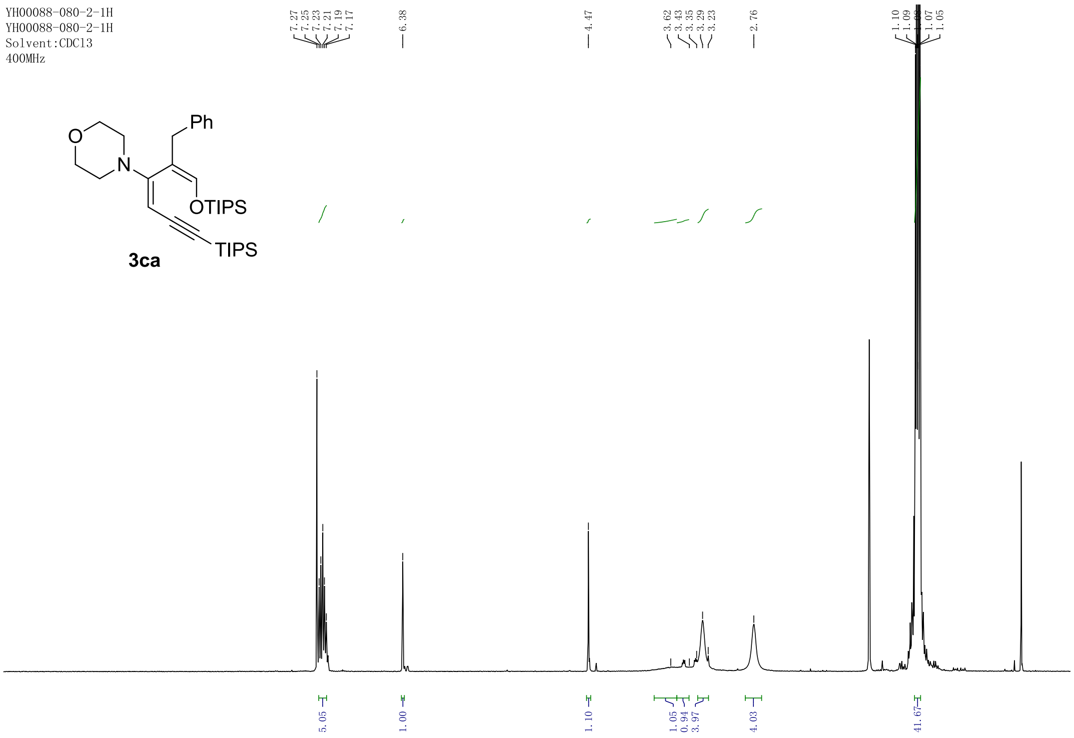

Supplementary Figure 31. ^{1}**H NMR (400MHz, CDCl**$_3$**) spectrum for 3ca**

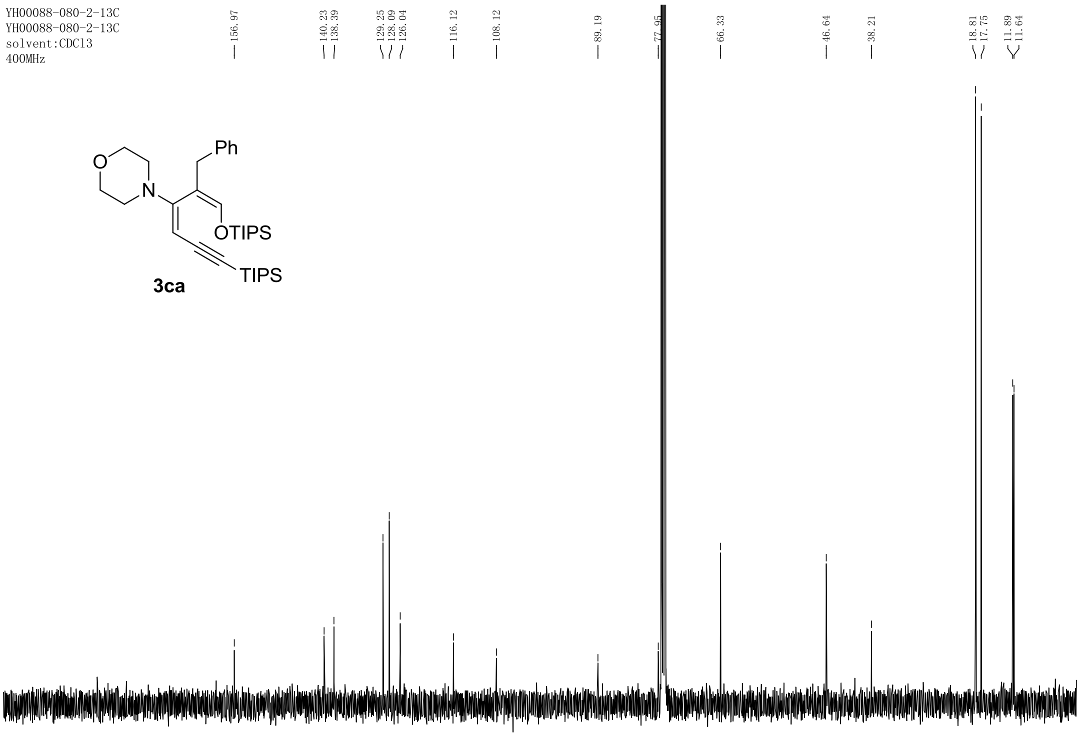

Supplementary Figure 32. ^{13}**C NMR (101MHz, CDCl**$_3$**) spectrum for 3ca**

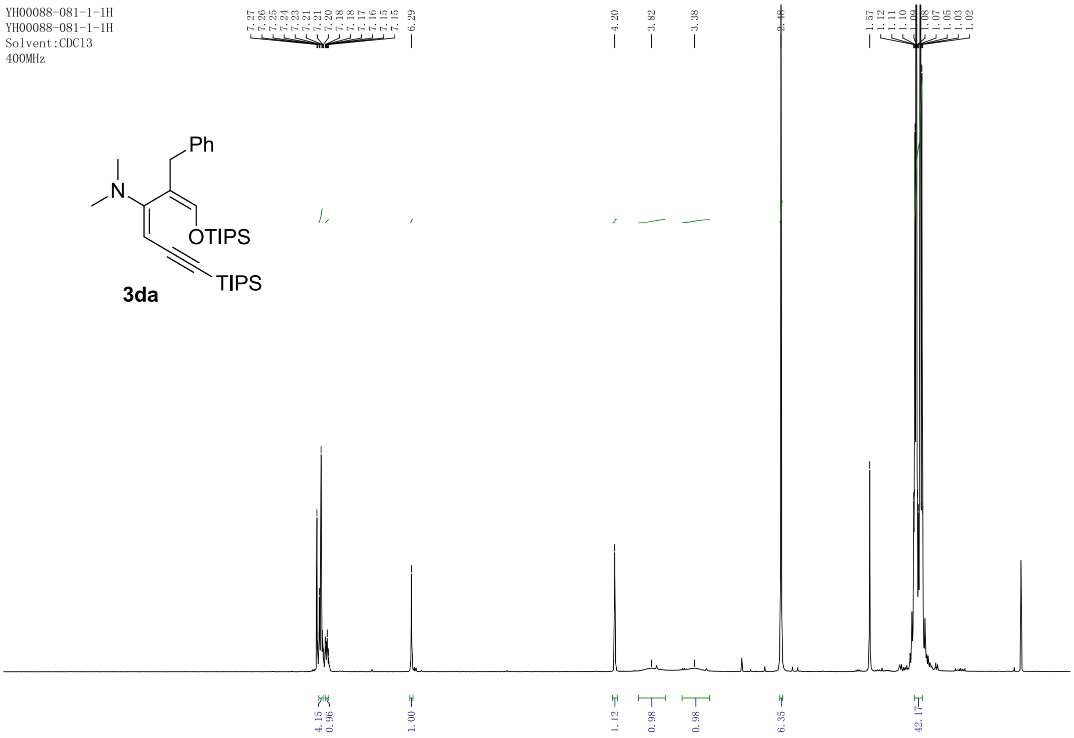

Supplementary Figure 33. ^{1}H NMR (400MHz, $CDCl_3$) spectrum for 3da

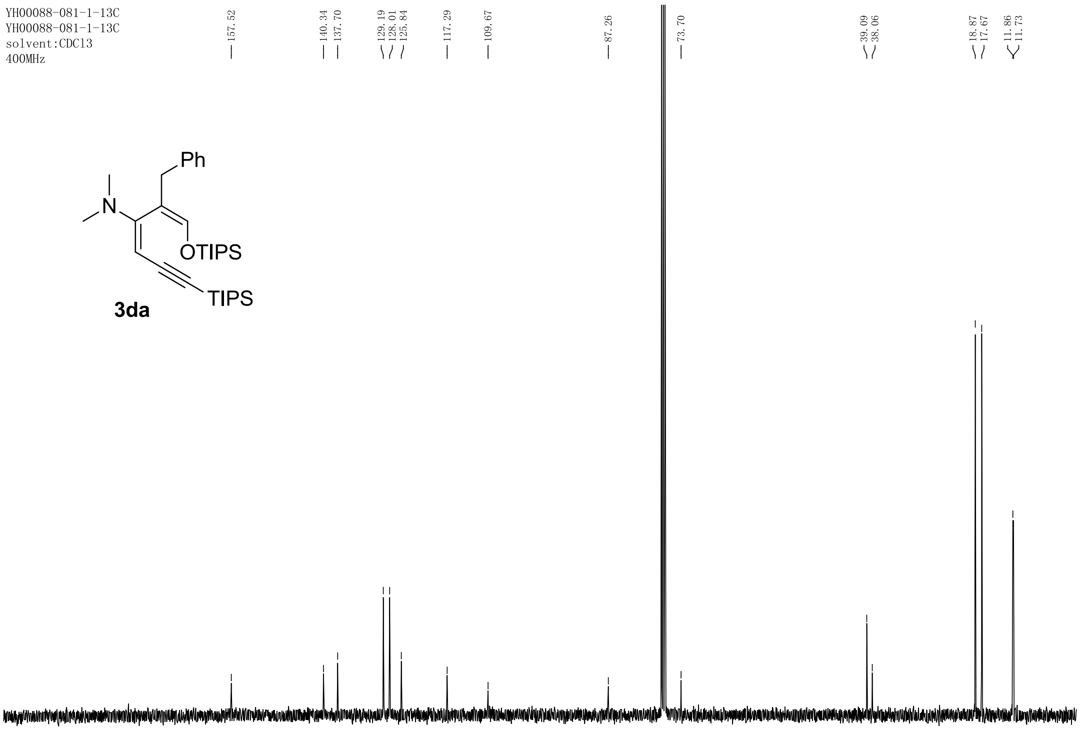

Supplementary Figure 34. ^{13}C NMR (101MHz, $CDCl_3$) spectrum for 3da

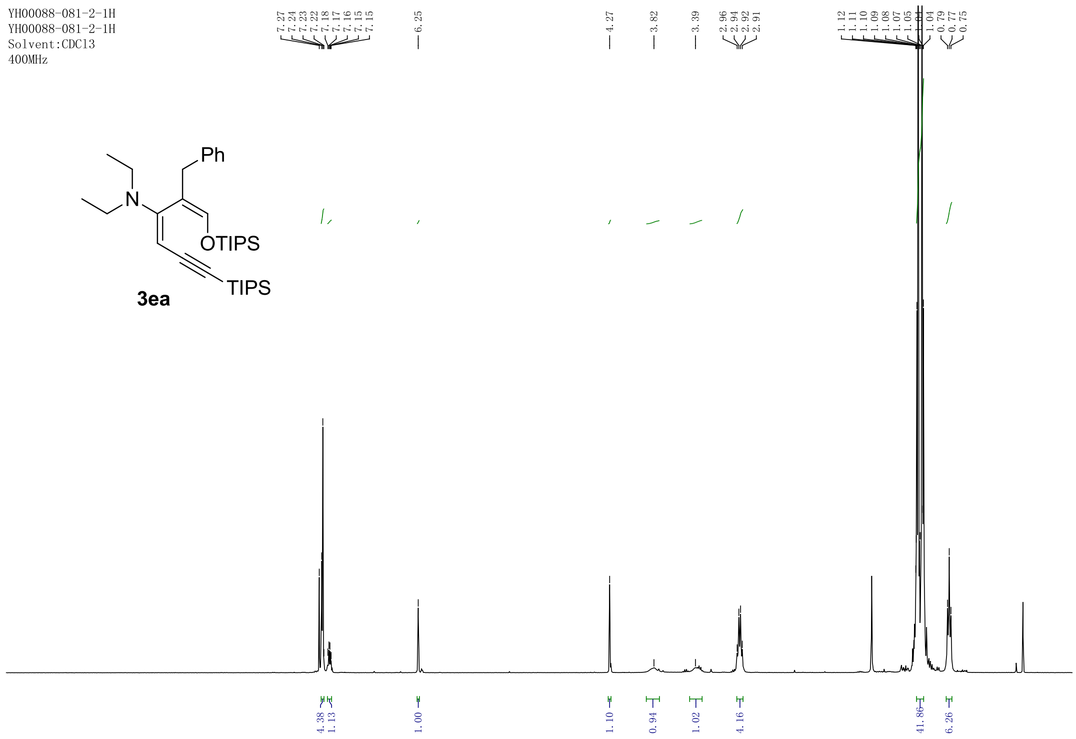

Supplementary Figure 35. ^{1}H NMR (400MHz, $CDCl_3$) spectrum for 3ea

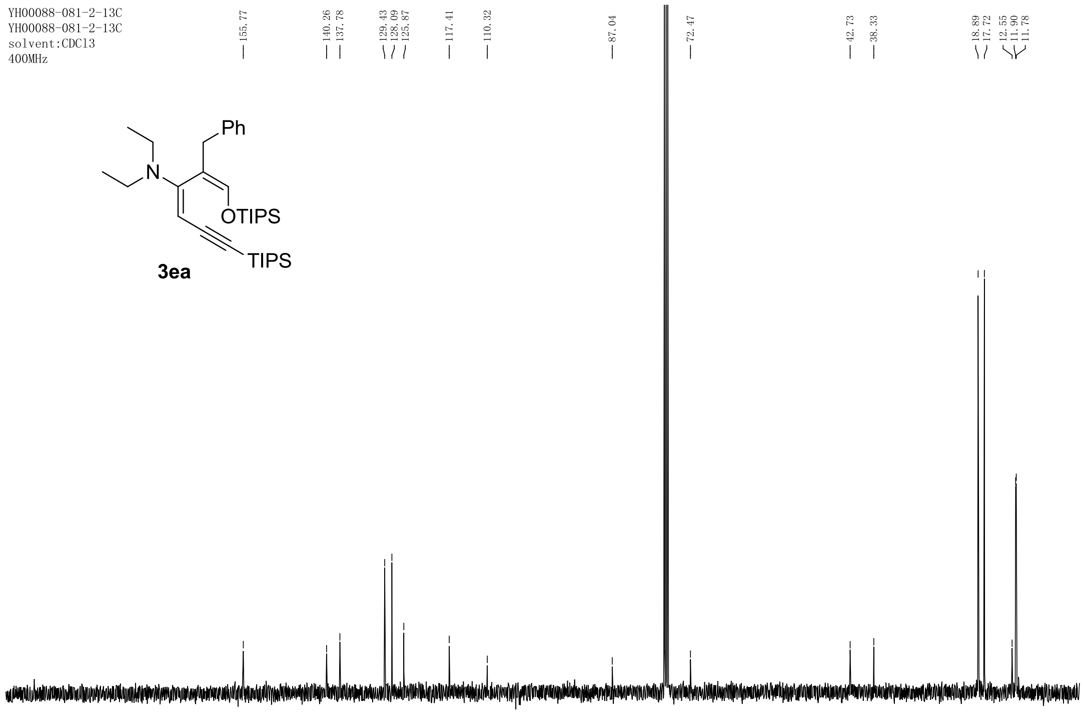

Supplementary Figure 36. ^{13}C NMR (101MHz, $CDCl_3$) spectrum for 3ea

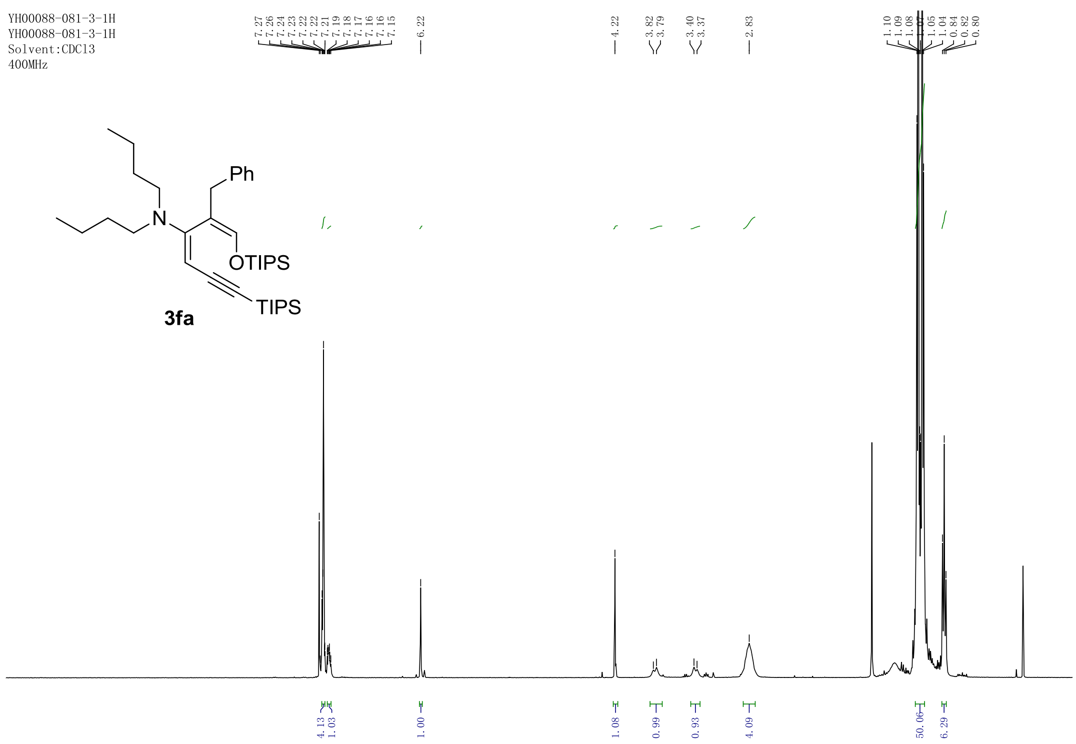

Supplementary Figure 37. ^{1}H NMR (400MHz, $CDCl_3$) spectrum for 3fa

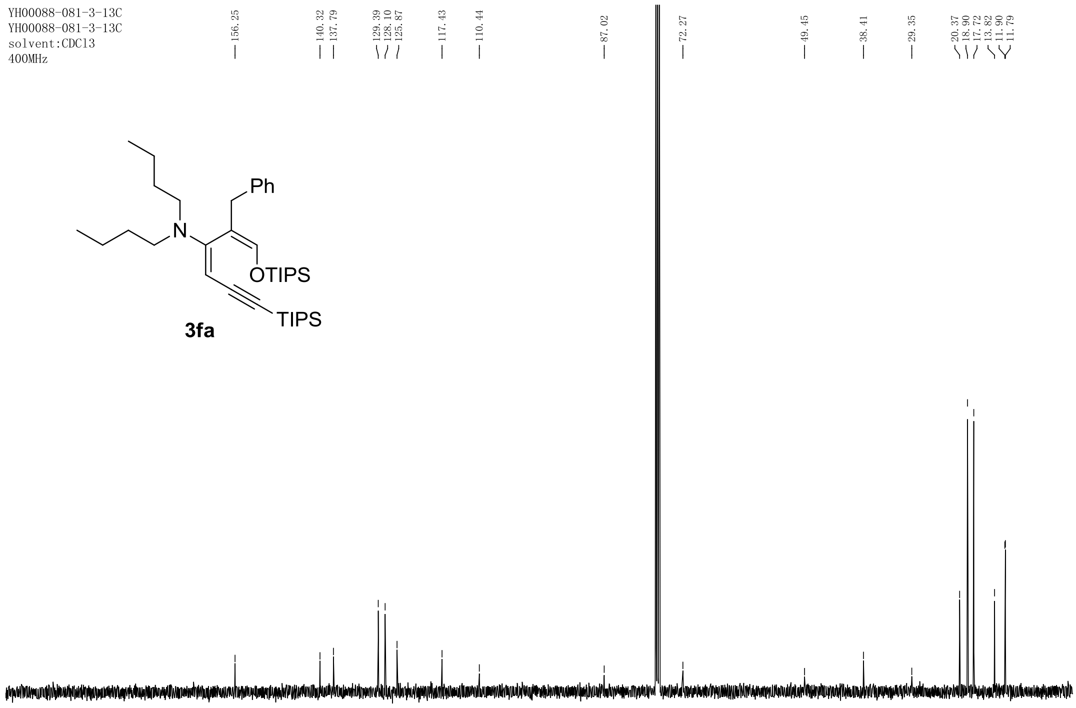

Supplementary Figure 38. ^{13}C NMR (101MHz, $CDCl_3$) spectrum for 3fa

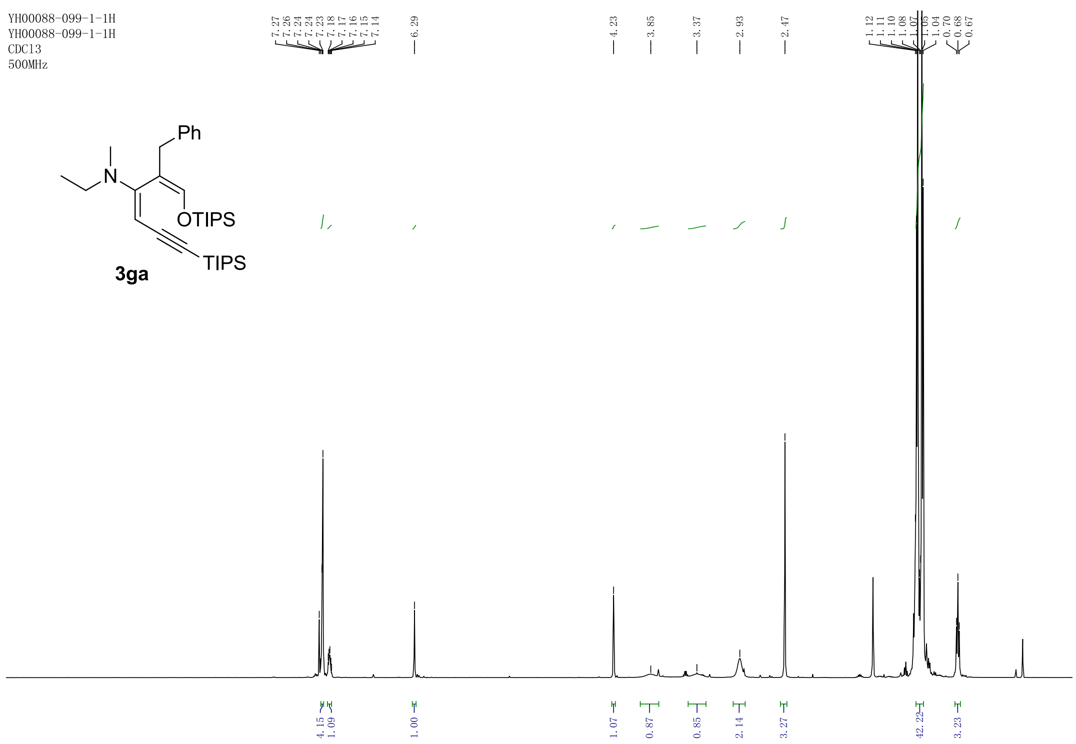

Supplementary Figure 39. ^{1}H NMR (500MHz, CDCl₃) spectrum for 3ga

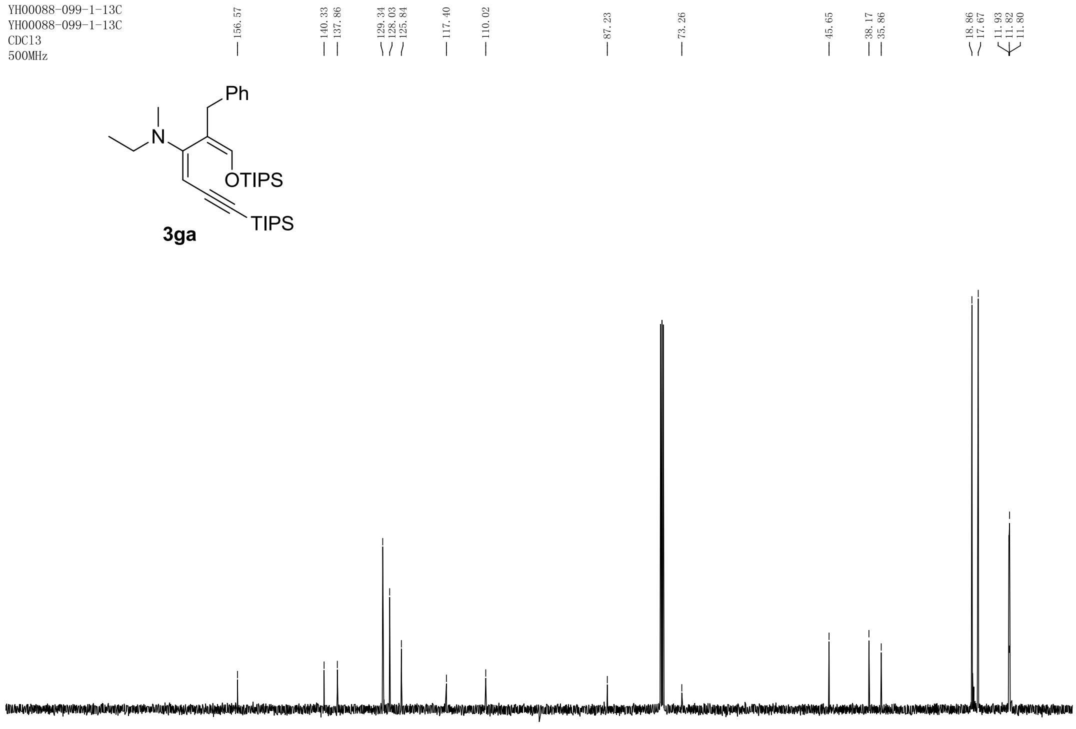

Supplementary Figure 40. ^{13}C NMR (126MHz, CDCl₃) spectrum for 3ga

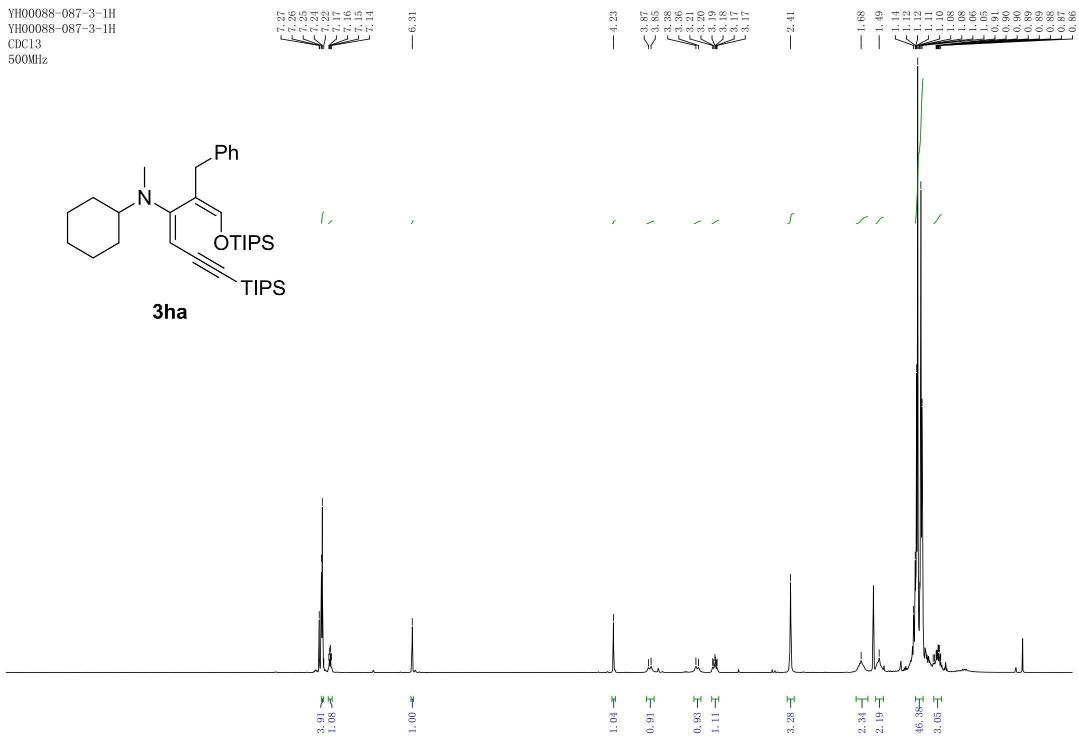

Supplementary Figure 41. ^{1}H NMR (500MHz, CDCl$_3$) spectrum for 3ha

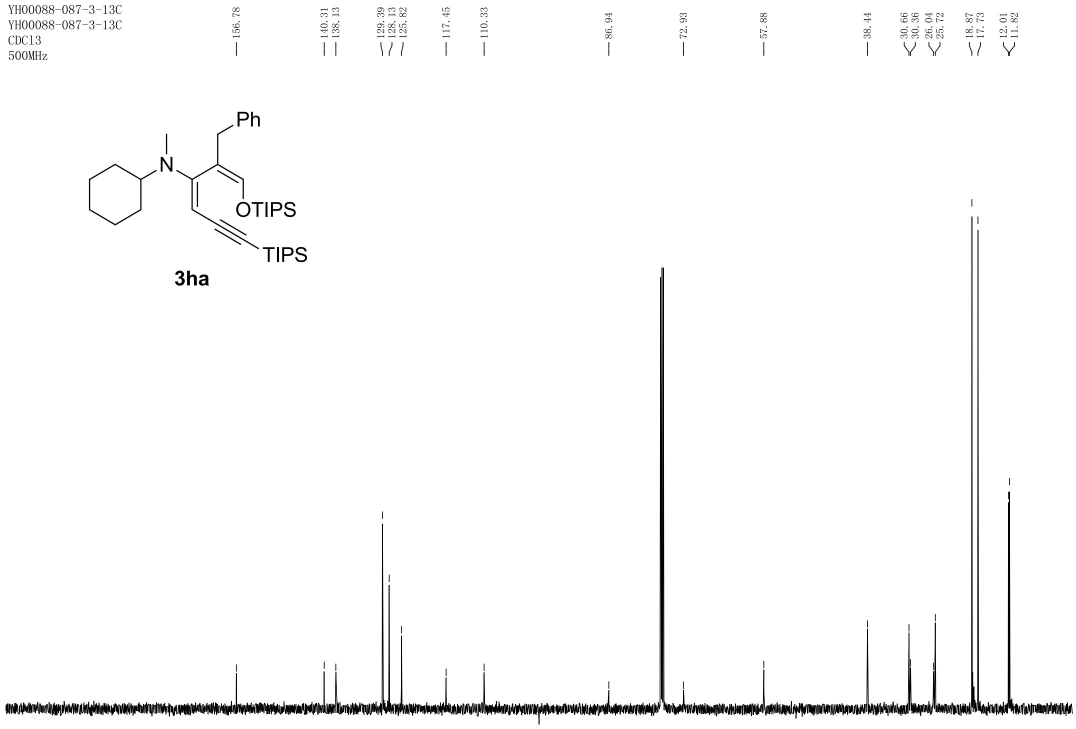

Supplementary Figure 42. ^{13}C NMR (126MHz, CDCl$_3$) spectrum for 3ha

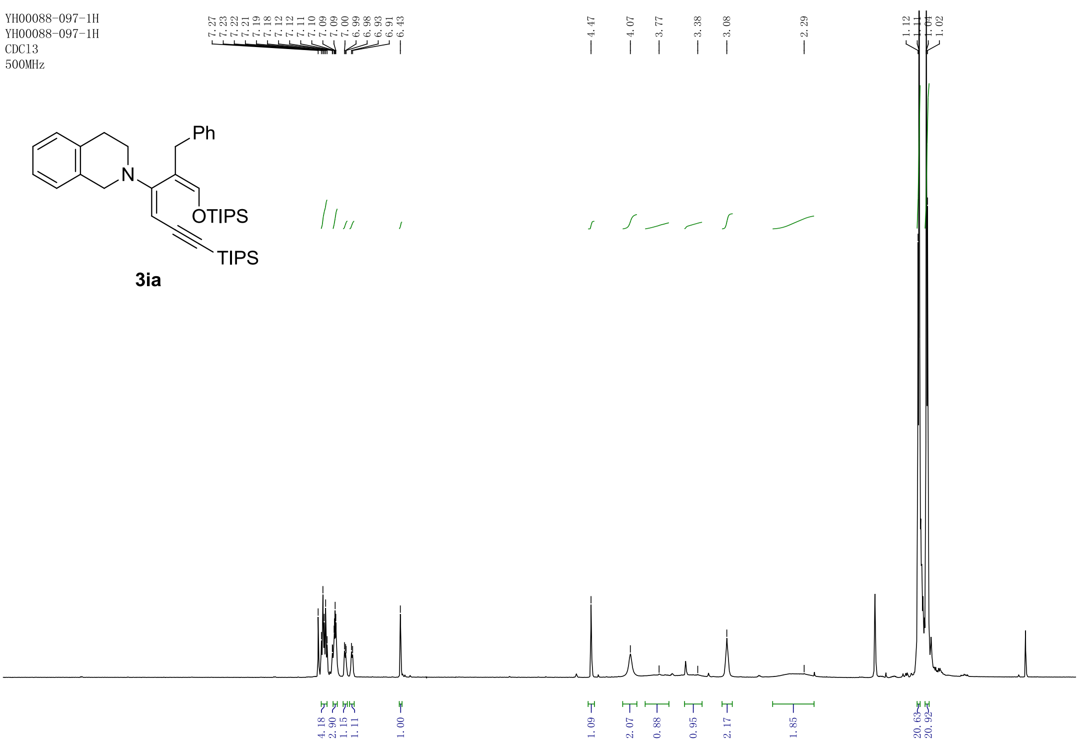

Supplementary Figure 43. **^{1}H NMR (500MHz, $CDCl_3$) spectrum for 3ia**

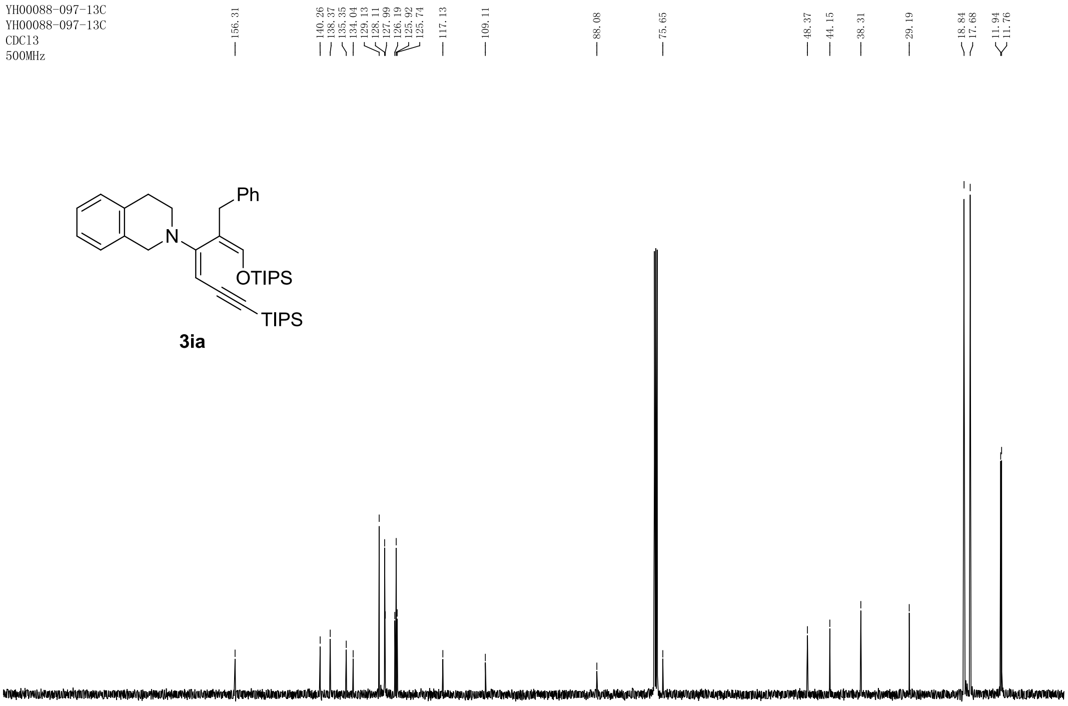

Supplementary Figure 44. **^{13}C NMR (126MHz, $CDCl_3$) spectrum for 3ia**

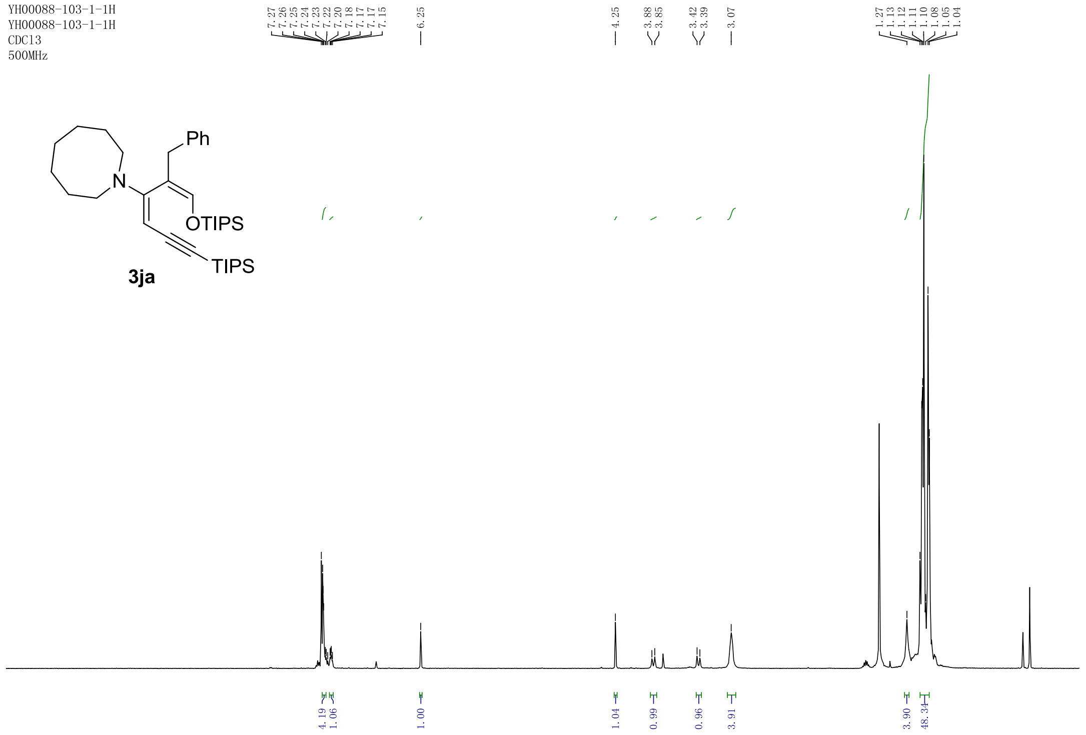

Supplementary Figure 45. ^{1}**H NMR (500MHz, CDCl₃) spectrum for 3ja**

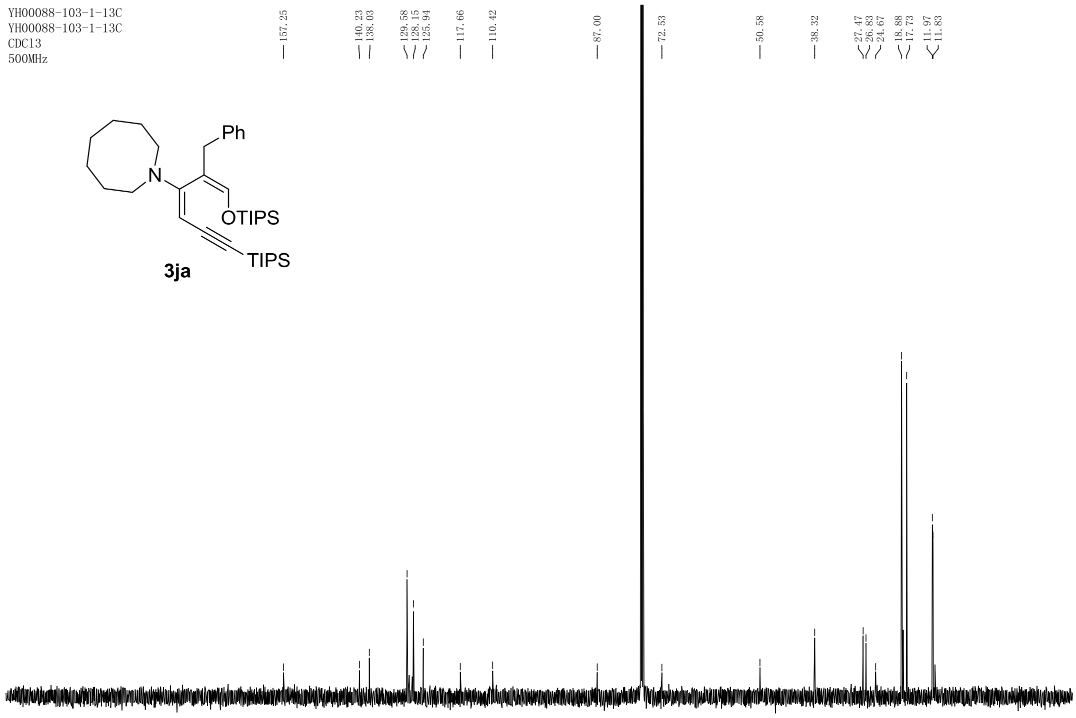

Supplementary Figure 46. ^{13}**C NMR (126MHz, CDCl₃) spectrum for 3ja**

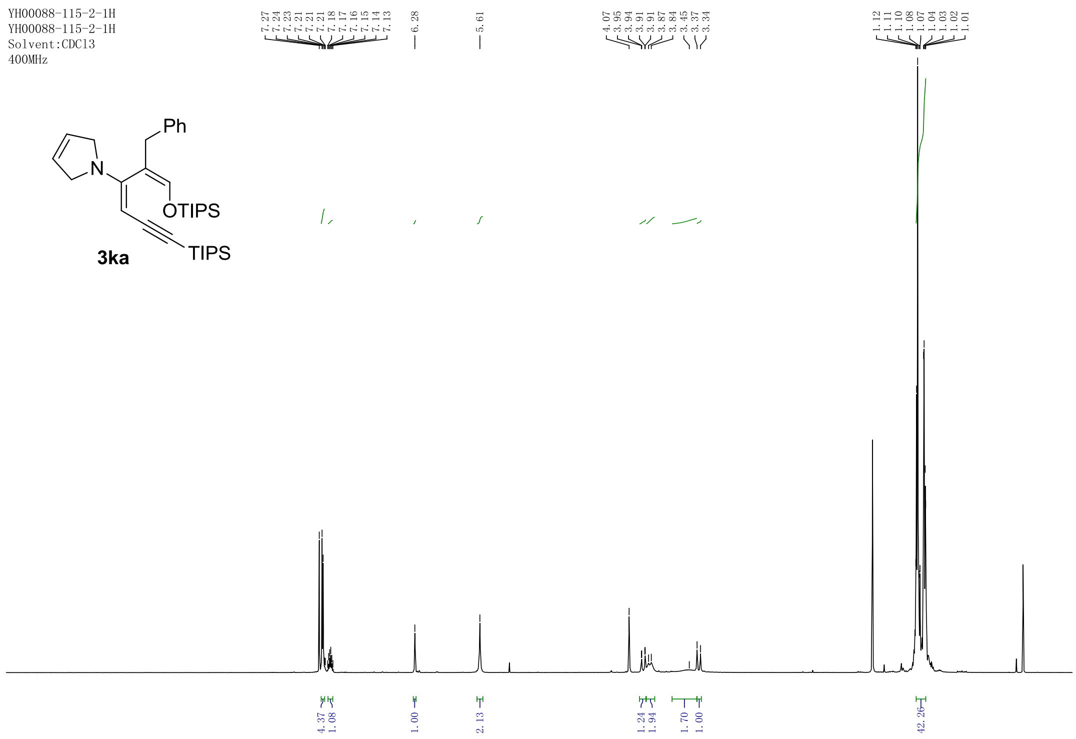

Supplementary Figure 47. ^{1}H NMR (400MHz, $CDCl_3$) spectrum for 3ka

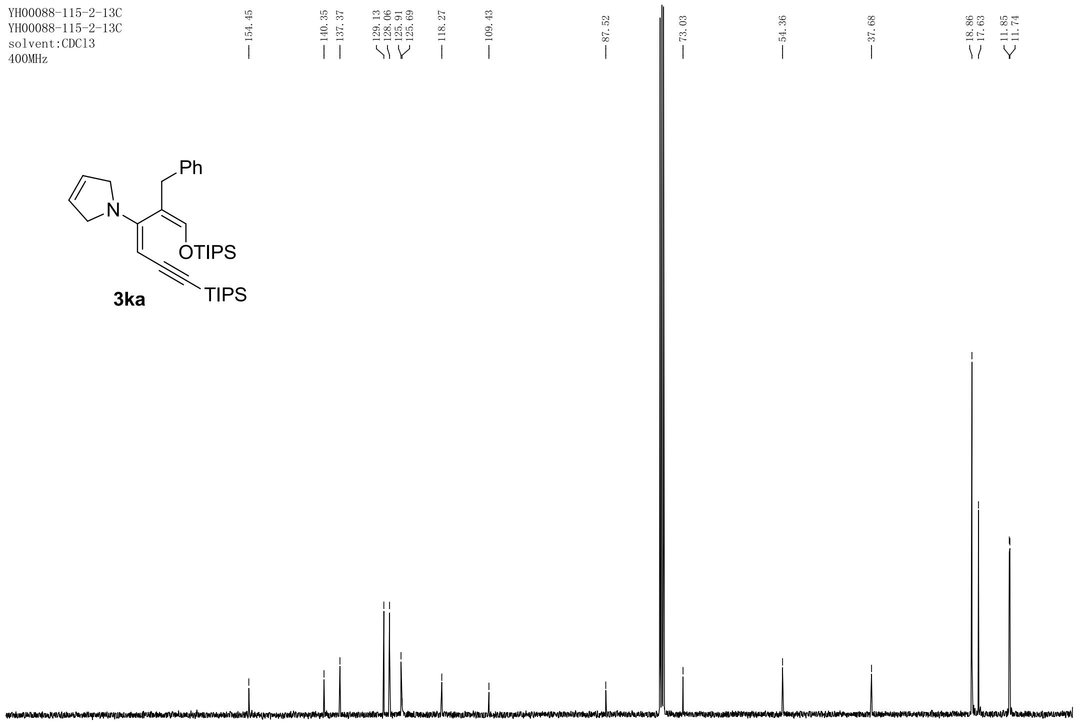

Supplementary Figure 48. ^{13}C NMR (101MHz, $CDCl_3$) spectrum for 3ka

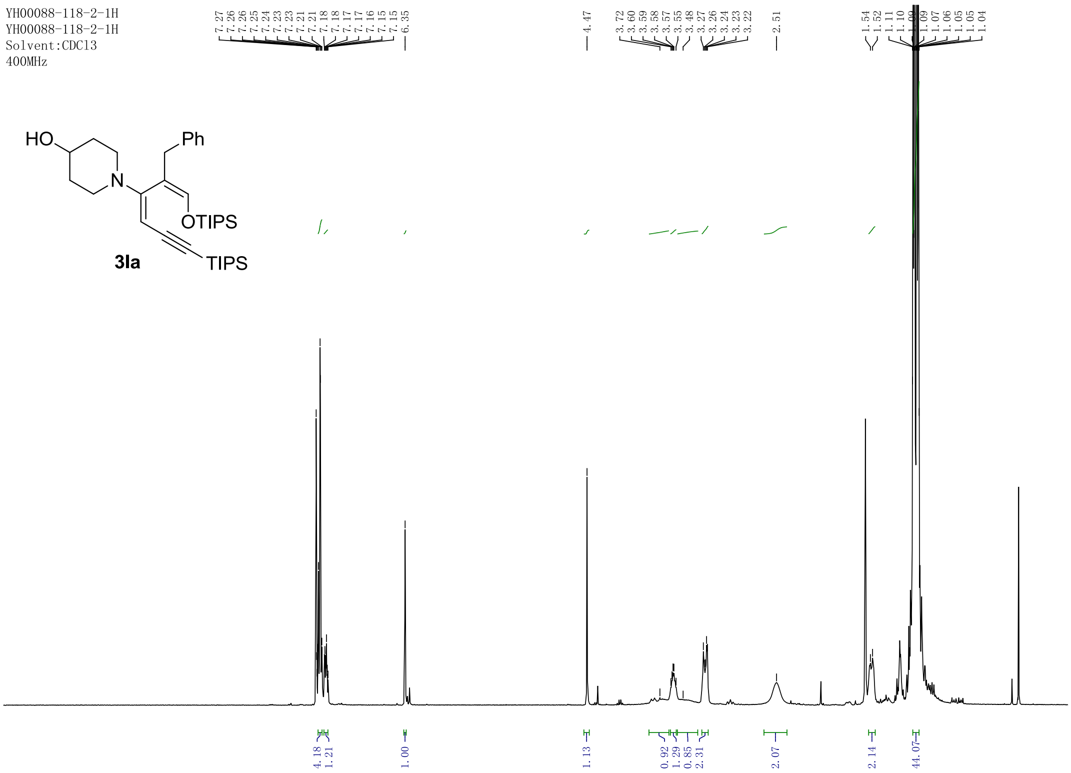

Supplementary Figure 49. ^{1}H NMR (400MHz, CDCl₃) spectrum for 3la

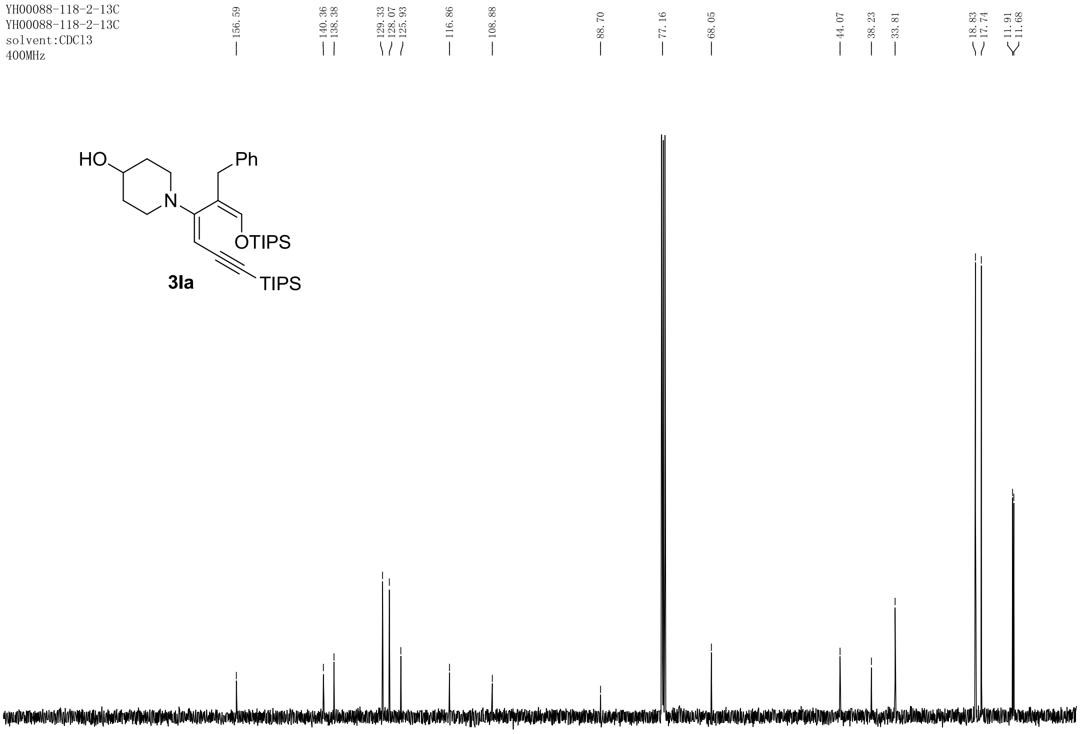

Supplementary Figure 50. ^{13}C NMR (101MHz, CDCl₃) spectrum for 3la

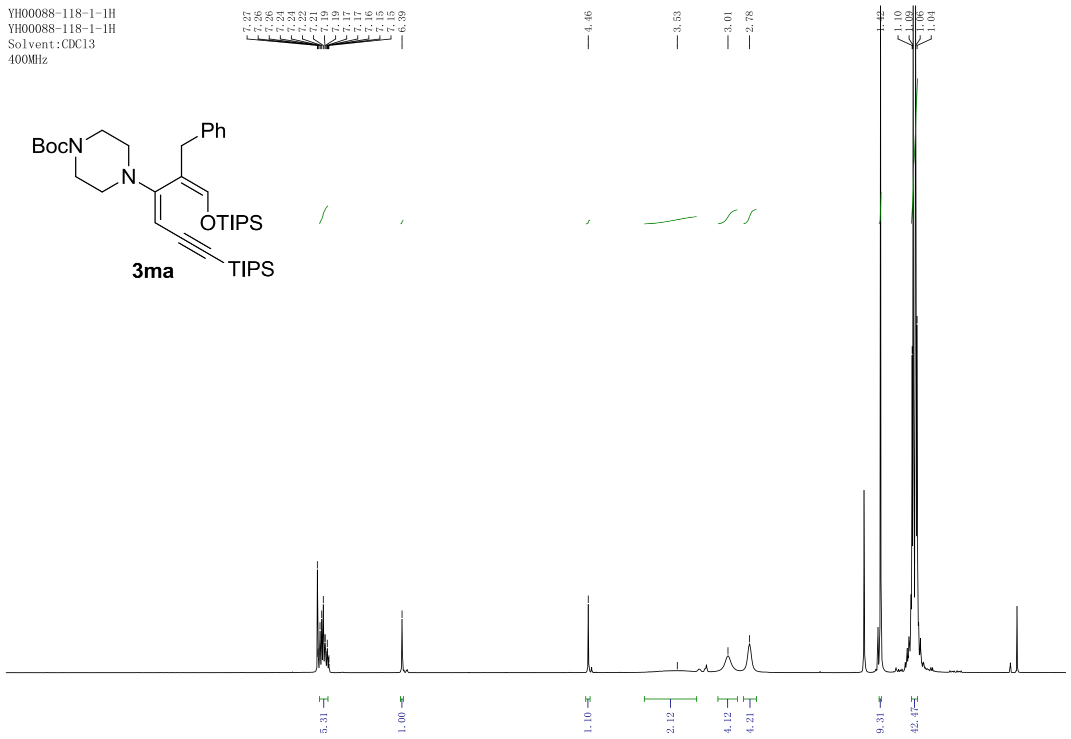

Supplementary Figure 51. ^{1}H NMR (400MHz, $CDCl_3$) spectrum for 3ma

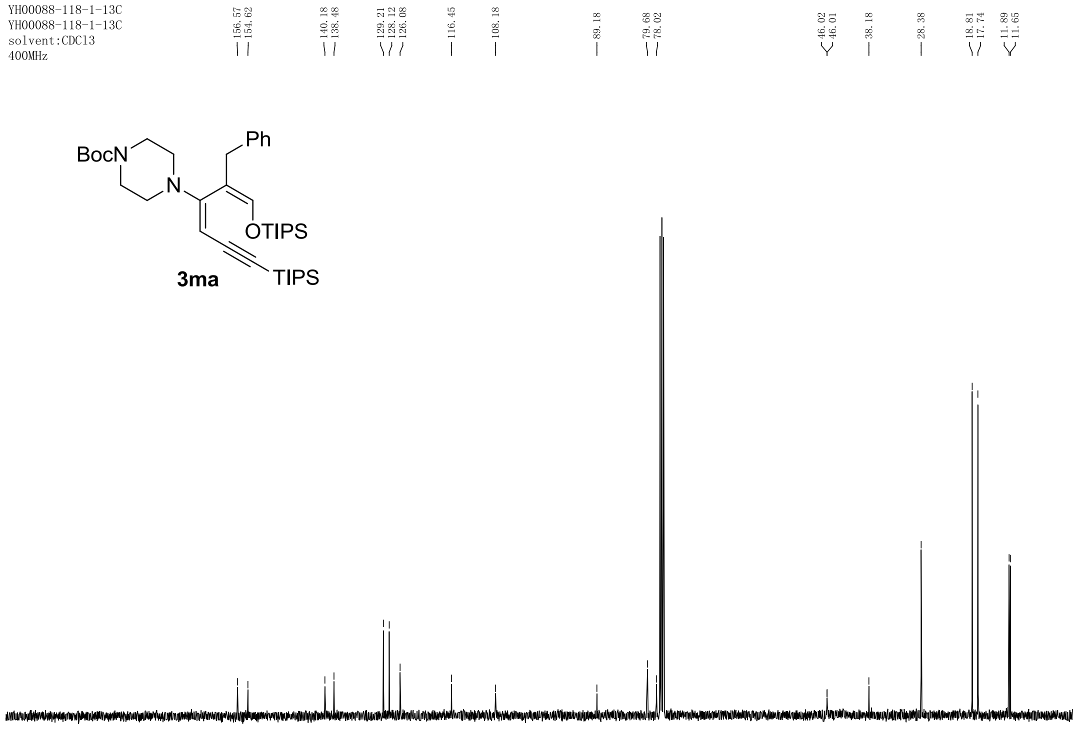

Supplementary Figure 52. ^{13}C NMR (101MHz, $CDCl_3$) spectrum for 3ma

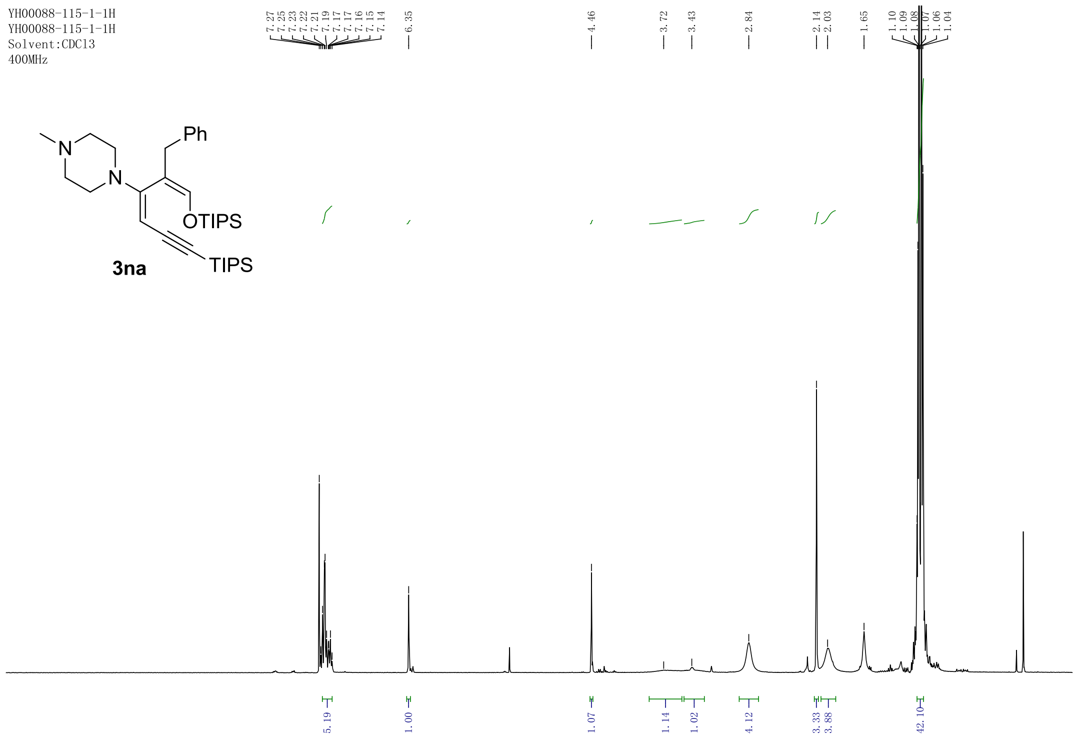

Supplementary Figure 53. ^{1}**H NMR (400MHz, CDCl₃) spectrum for 3na**

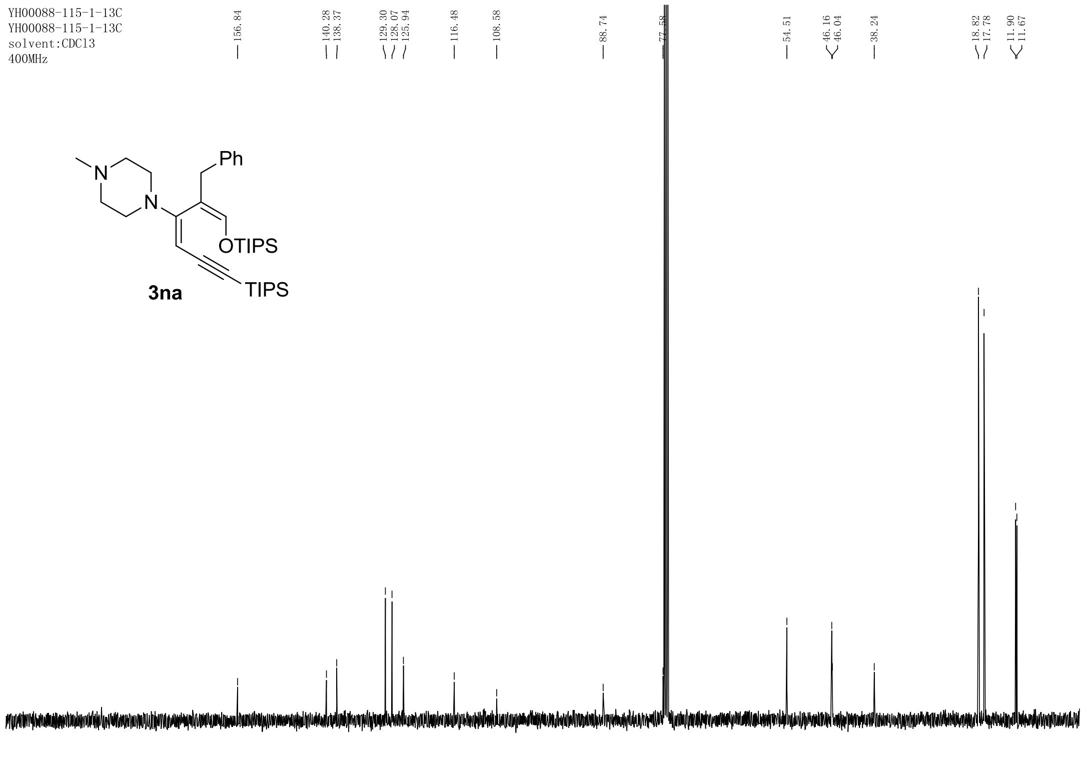

Supplementary Figure 54. ^{13}**C NMR (101MHz, CDCl₃) spectrum for 3na**

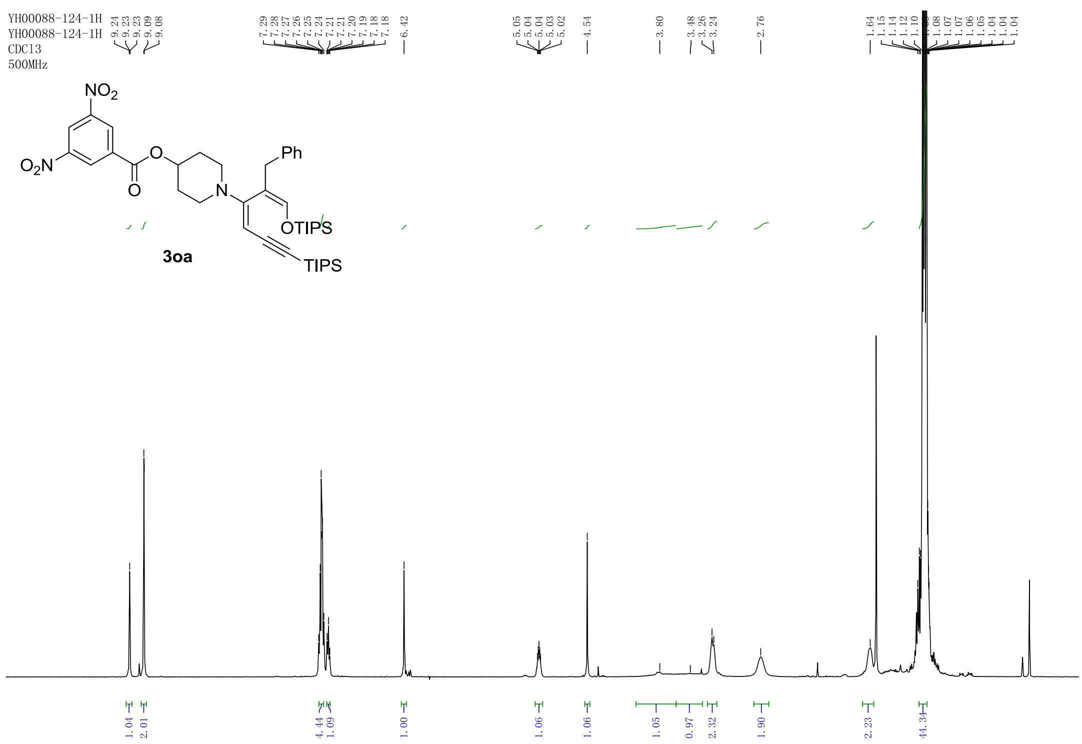

Supplementary Figure 55. ^{1}H NMR (500MHz, CDCl$_3$) spectrum for 3oa

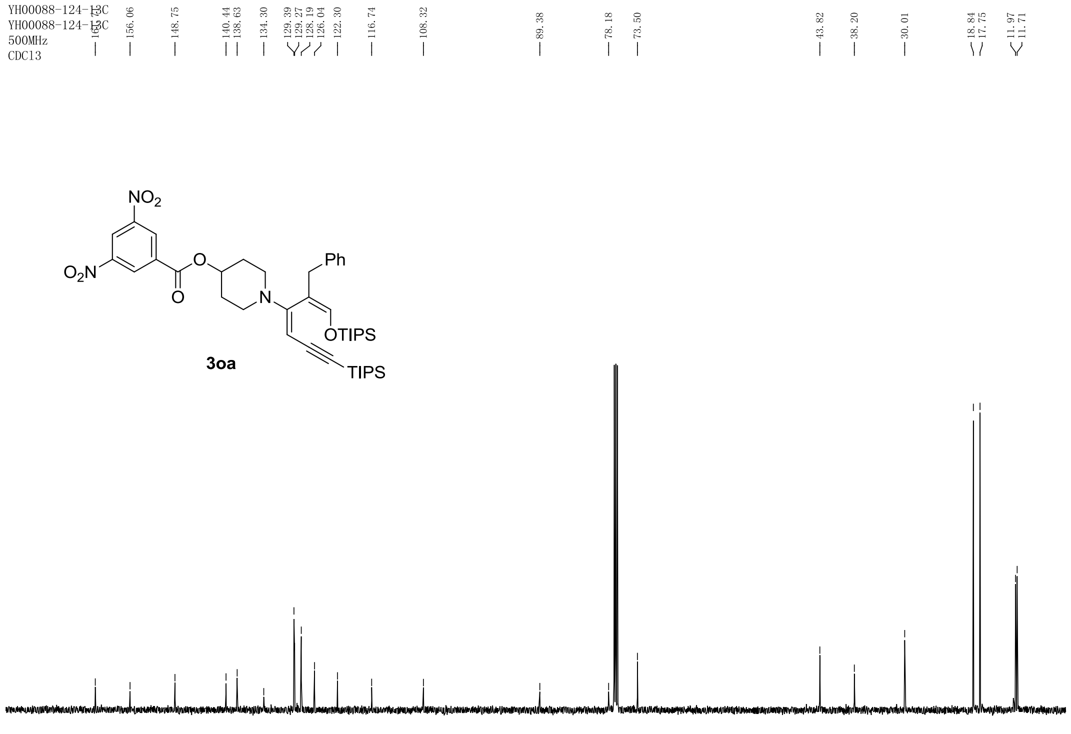

Supplementary Figure 56. ^{13}C NMR (126MHz, CDCl$_3$) spectrum for 3oa

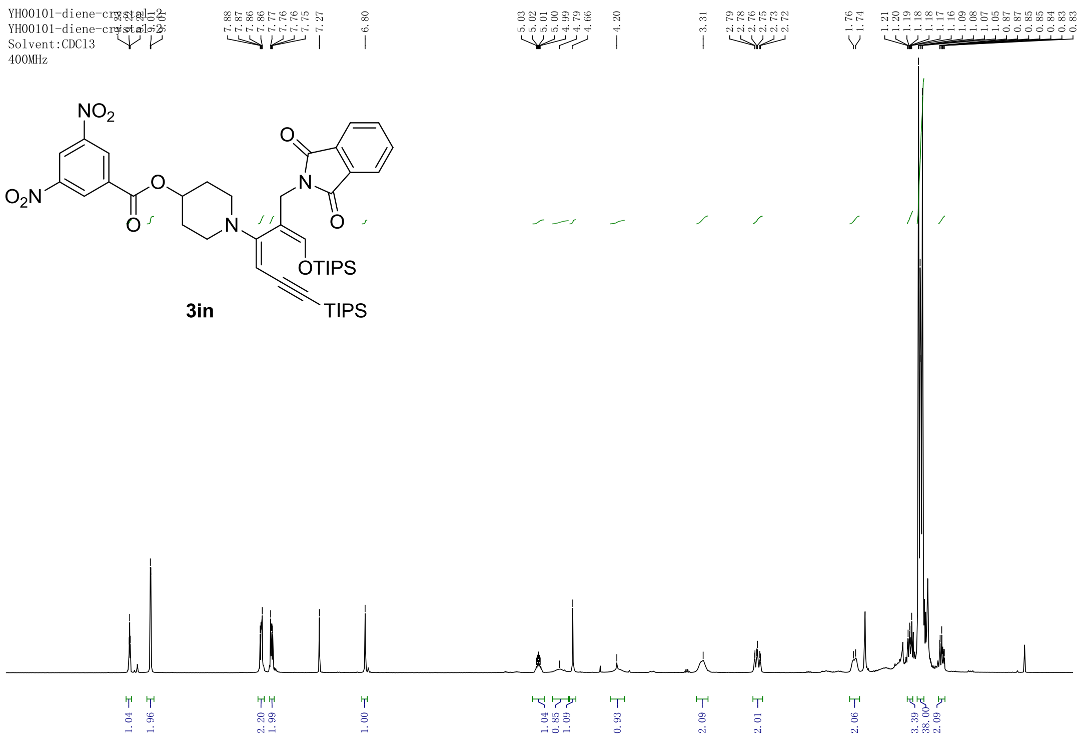

Supplementary Figure 57. ^{1}H NMR (400MHz, $CDCl_3$) spectrum for 3in

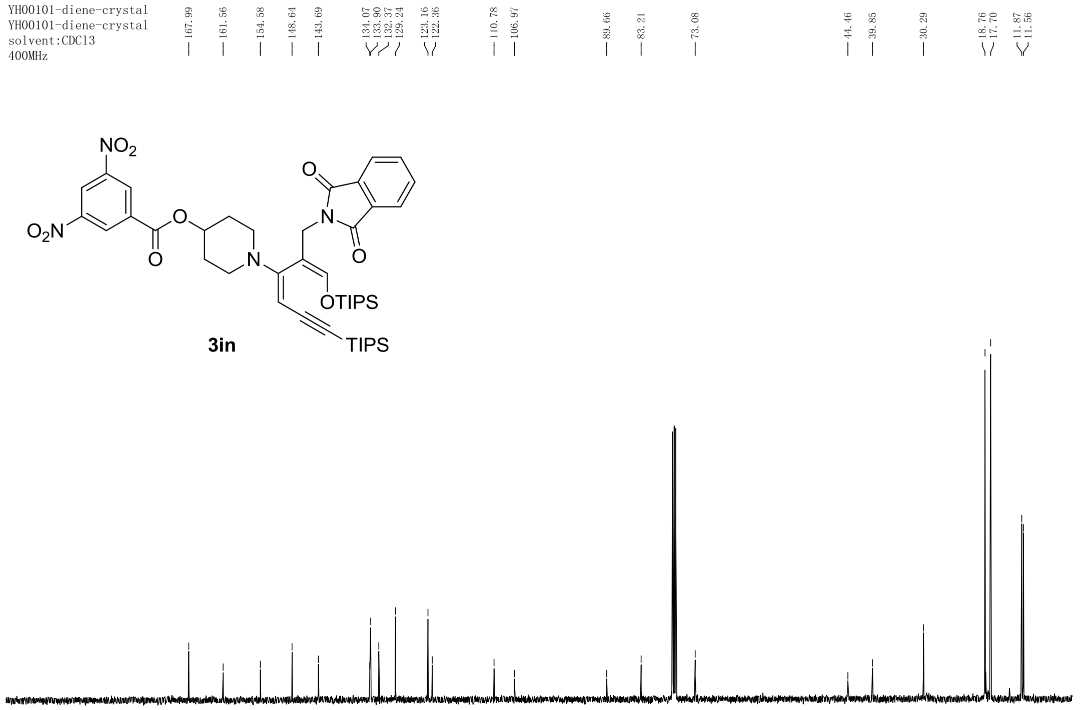

Supplementary Figure 58. ^{13}C NMR (101MHz, $CDCl_3$) spectrum for 3in

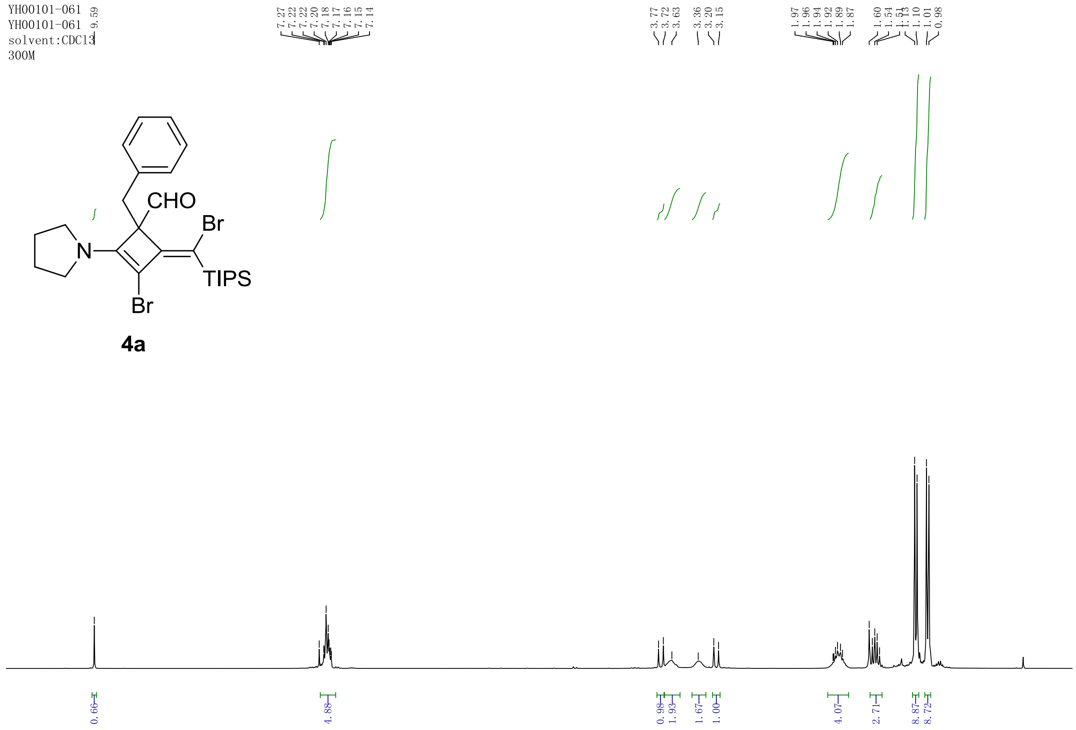

Supplementary Figure 59. ^{1}**H NMR (300MHz, CDCl₃) spectrum for 4a**

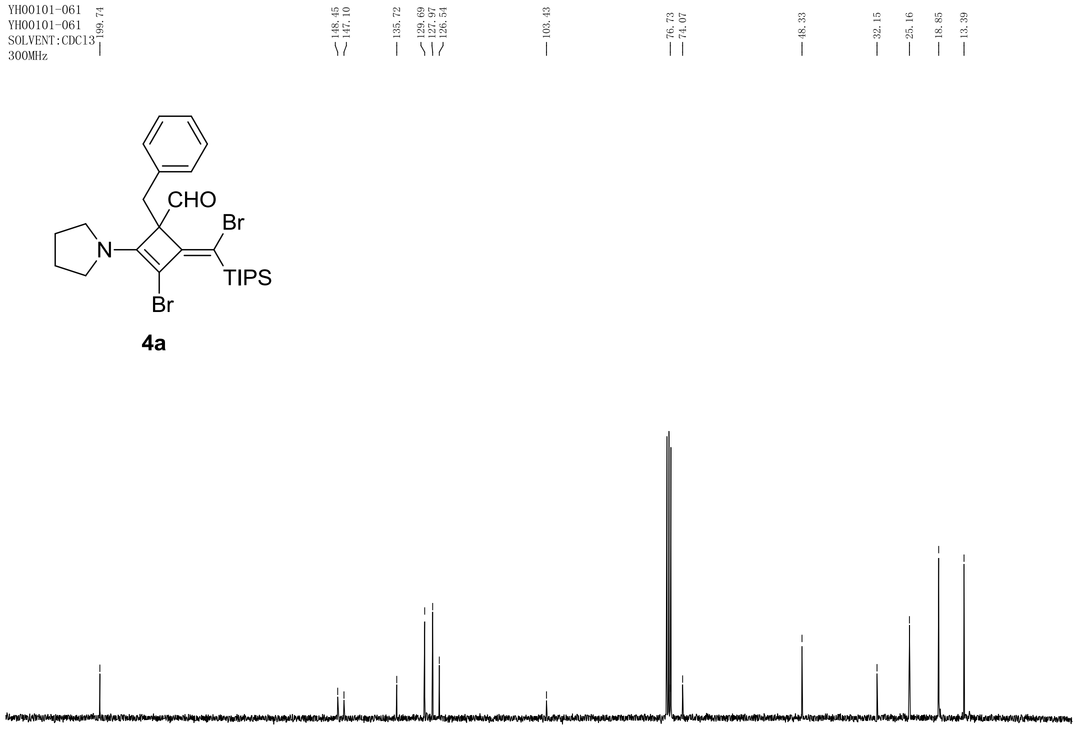

Supplementary Figure 60. ^{13}**C NMR (75MHz, CDCl₃) spectrum for 4a**

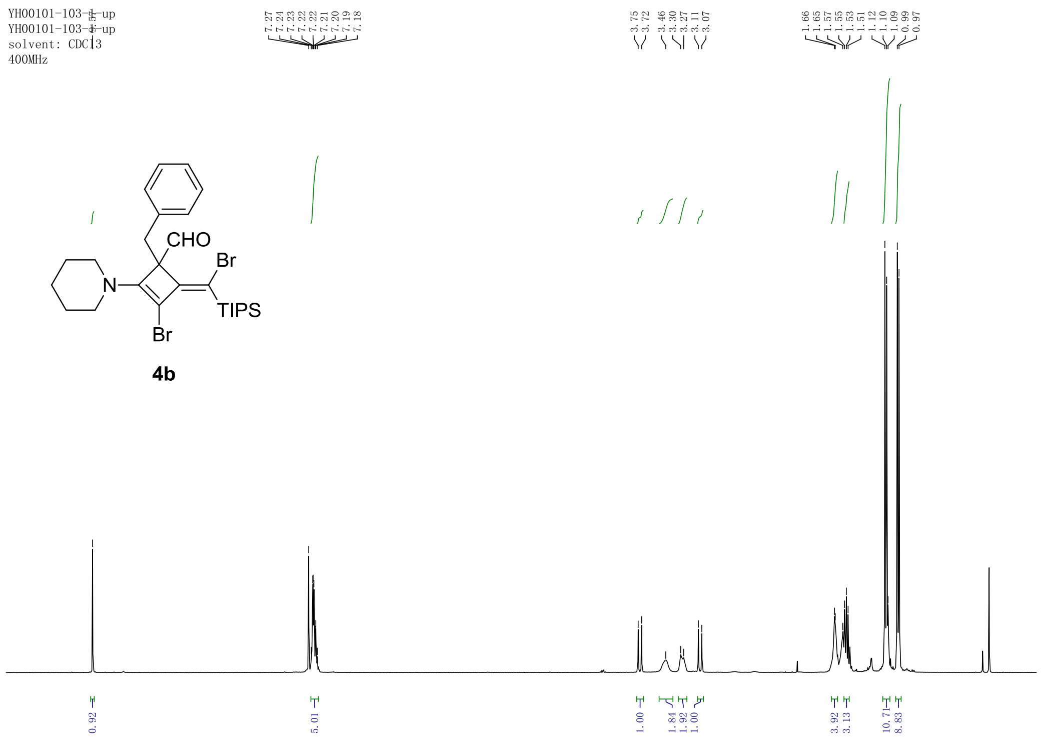

Supplementary Figure 61. ^{1}H NMR (400MHz, CDCl₃) spectrum for 4b

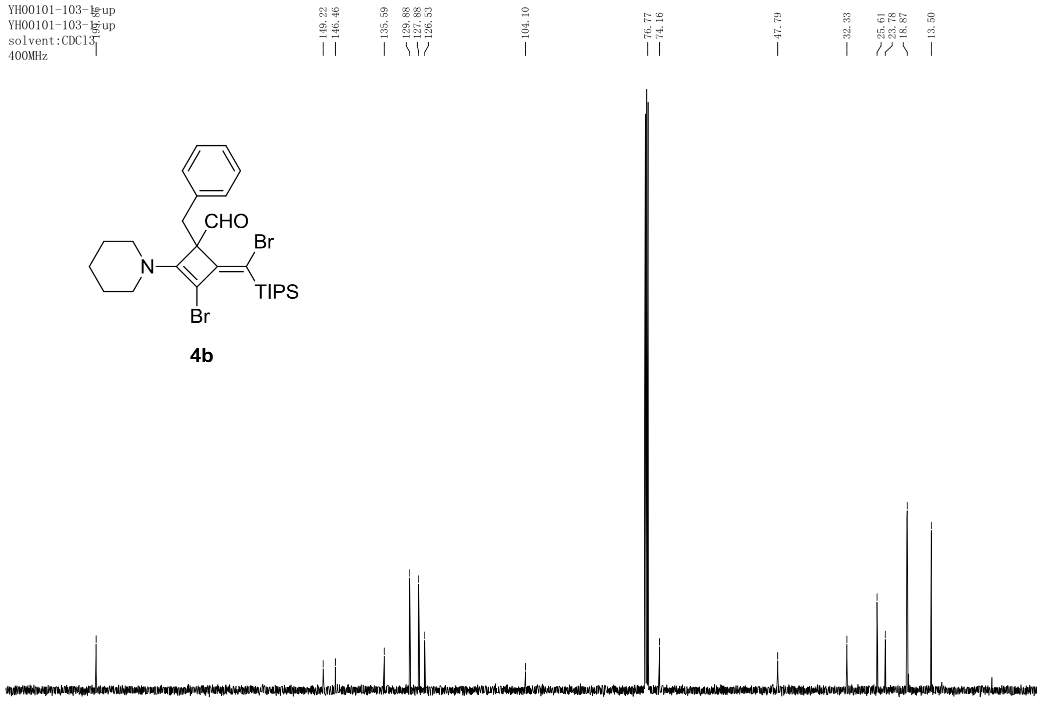

Supplementary Figure 62. ^{13}C NMR (101MHz, CDCl₃) spectrum for 4b

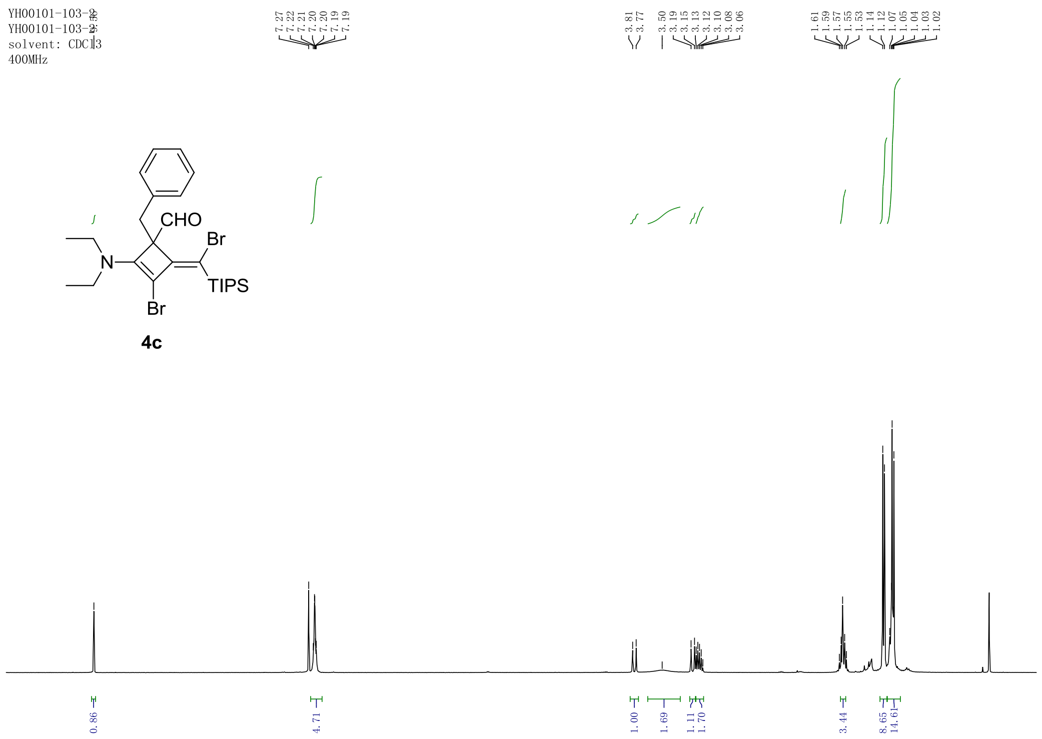

Supplementary Figure 63. **^{1}H NMR (400MHz, $CDCl_3$) spectrum for 4c**

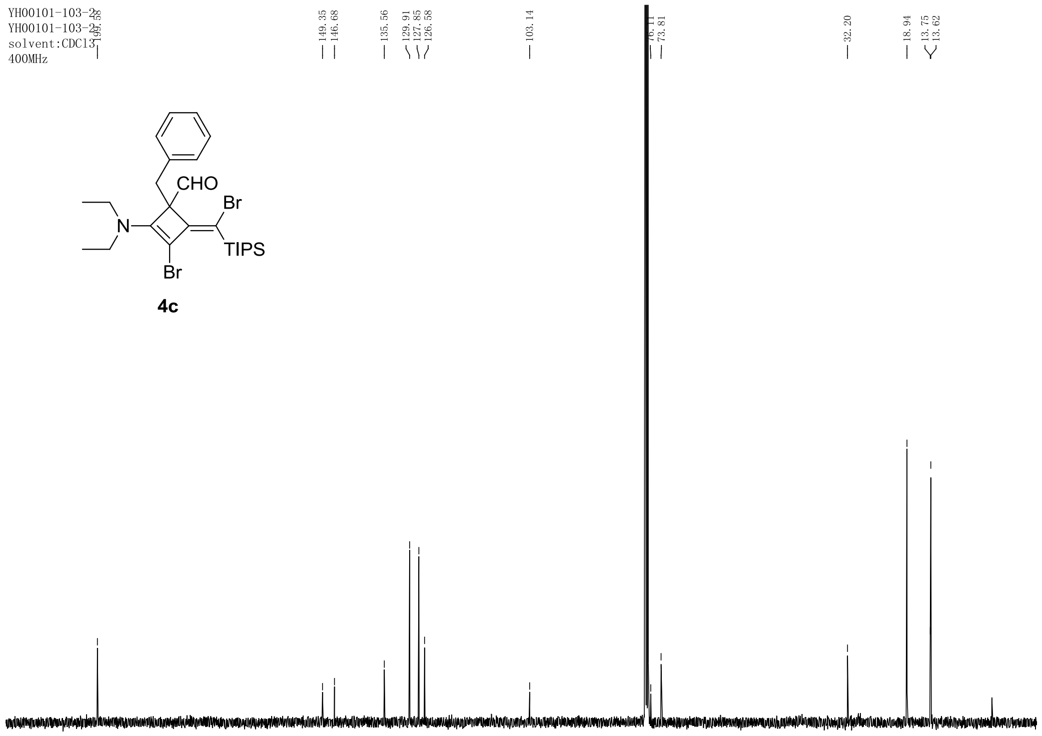

Supplementary Figure 64. **^{13}C NMR (101MHz, $CDCl_3$) spectrum for 4c**

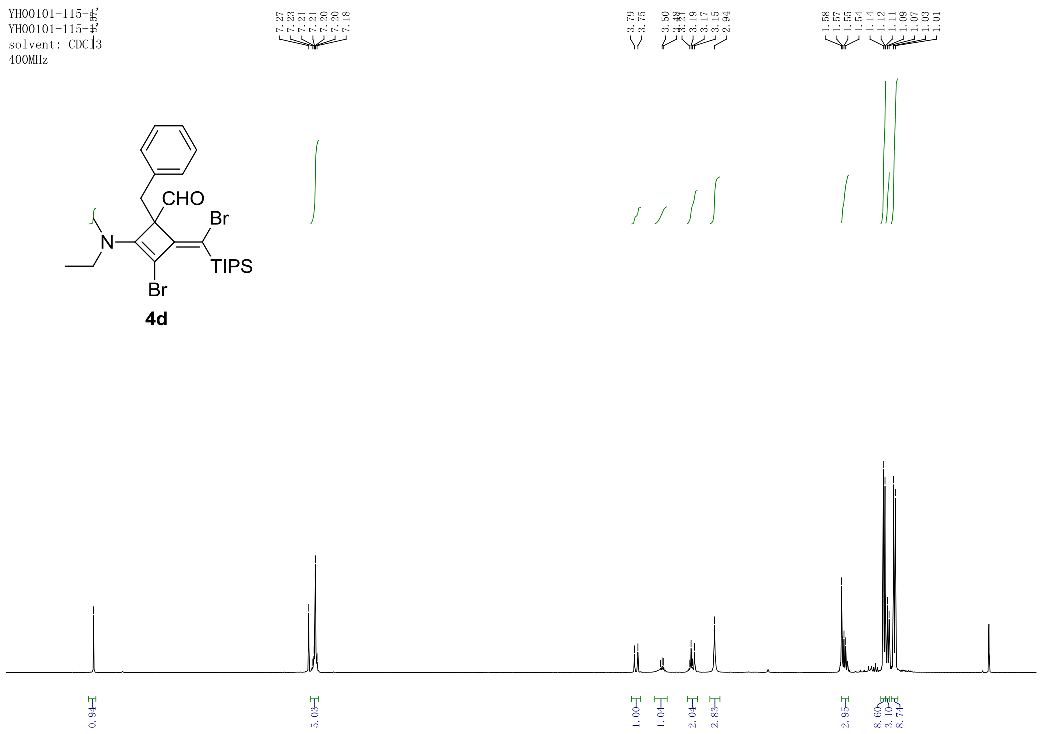

Supplementary Figure 65. ^{1}H NMR (400MHz, $CDCl_3$) spectrum for 4d

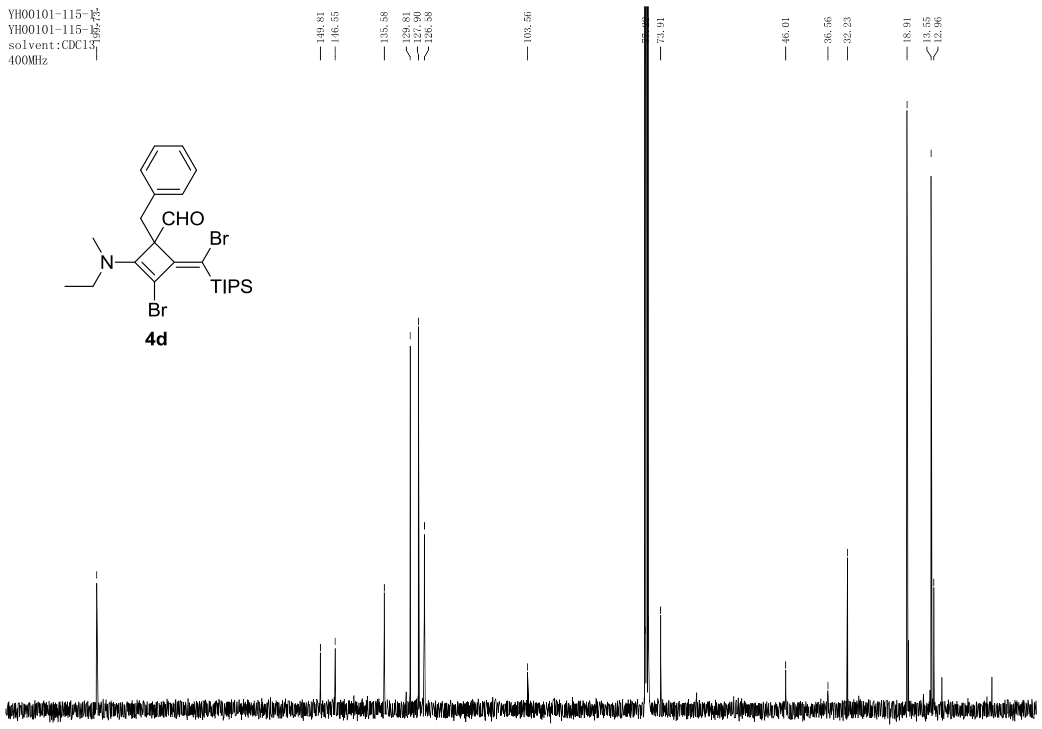

Supplementary Figure 66. ^{13}C NMR (101MHz, $CDCl_3$) spectrum for 4d

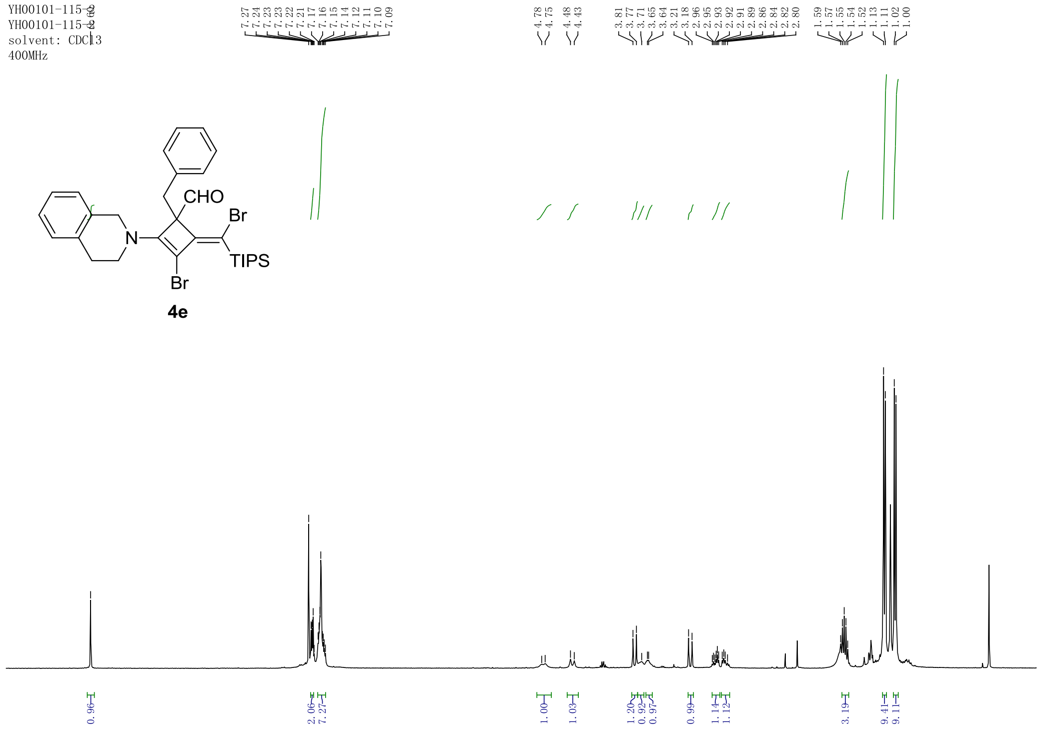

Supplementary Figure 67. ^{1}H NMR (400MHz, $CDCl_3$) spectrum for 4e

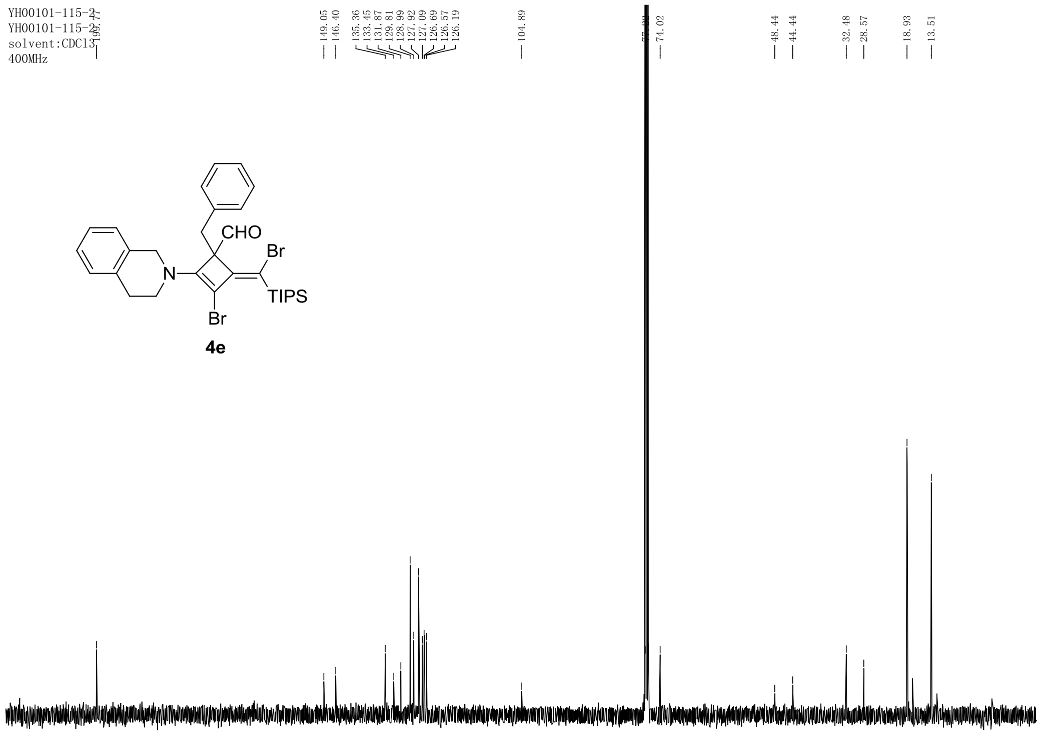

Supplementary Figure 68. ^{13}C NMR (101MHz, $CDCl_3$) spectrum for 4e

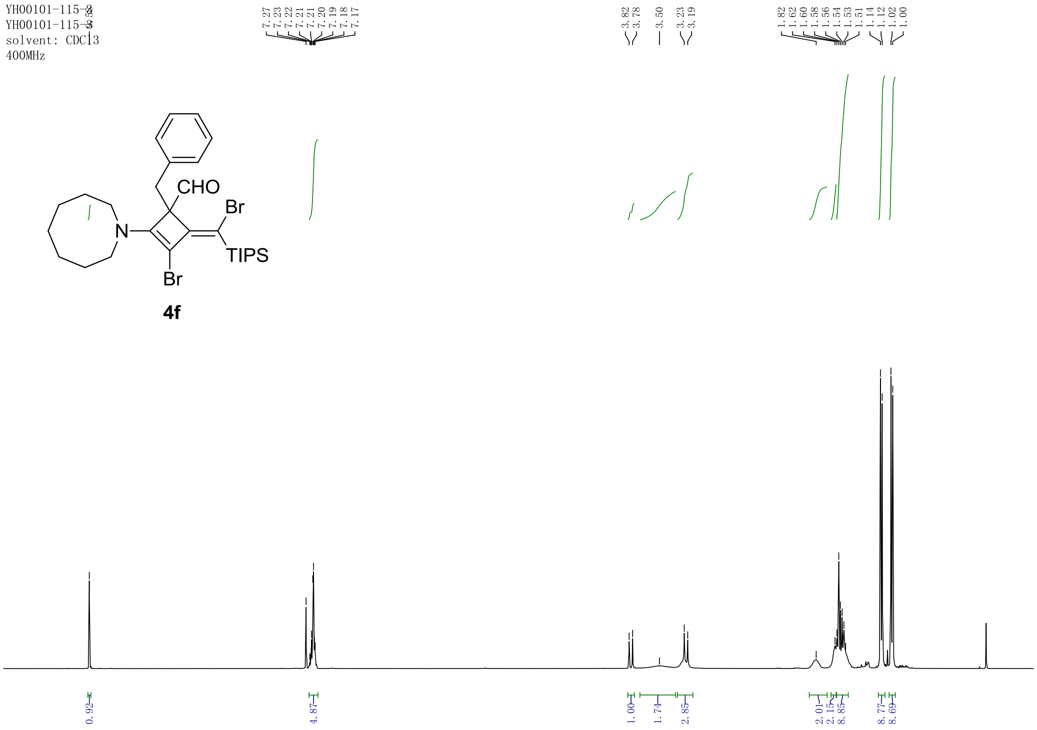

Supplementary Figure 69. ^{1}H NMR (400MHz, $CDCl_3$) spectrum for 4f

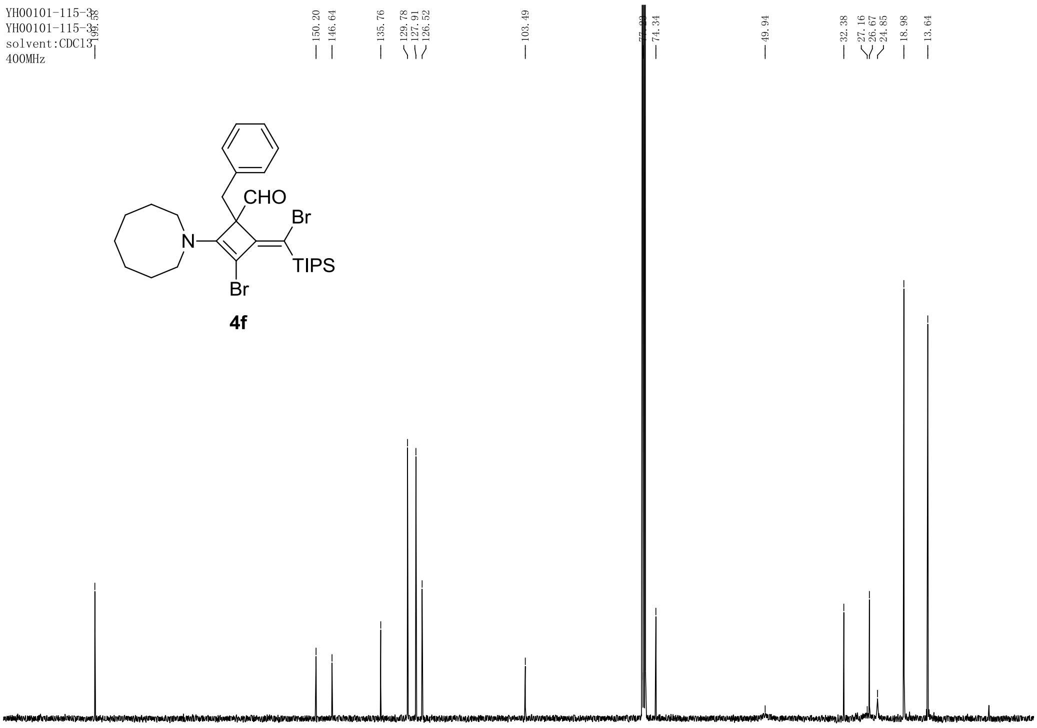

Supplementary Figure 70. ^{13}C NMR (101MHz, $CDCl_3$) spectrum for 4f

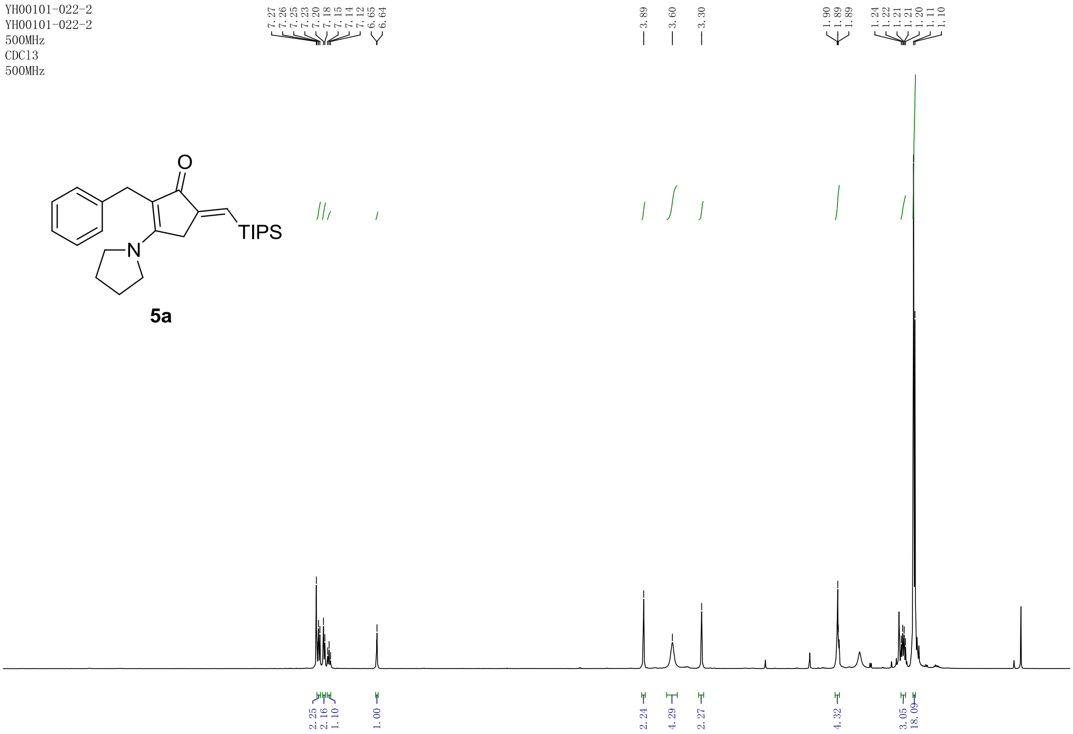

Supplementary Figure 71. ^{1}H NMR (500MHz, CDCl$_3$) spectrum for 5a

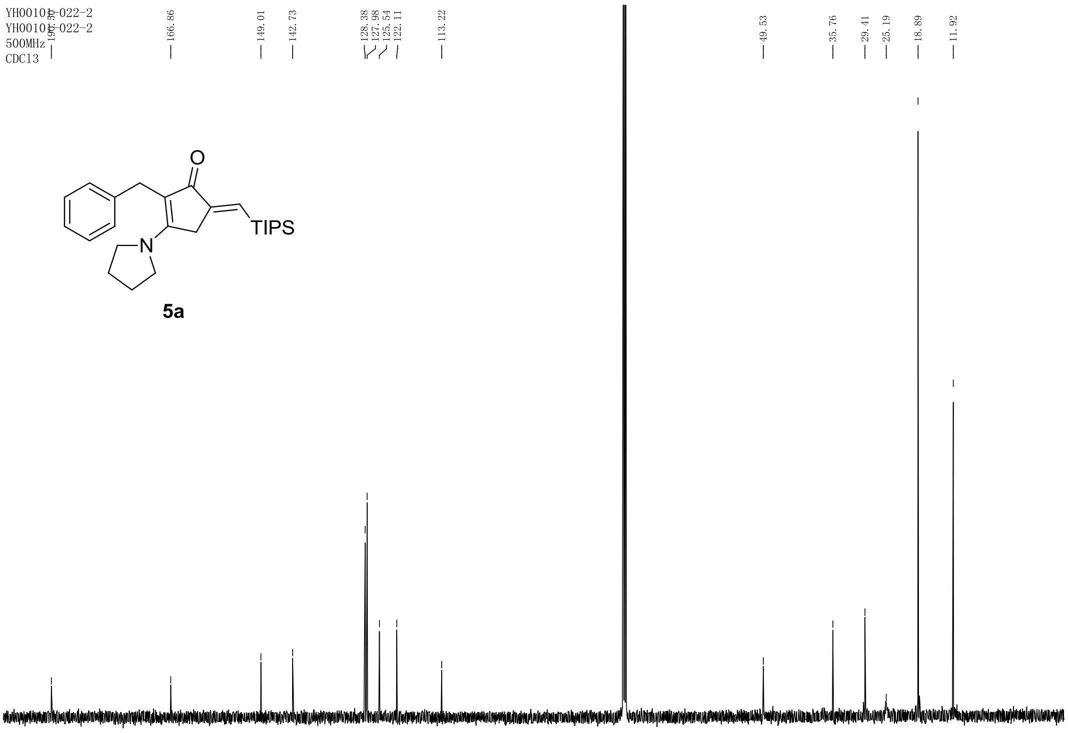

Supplementary Figure 72. ^{13}C NMR (126MHz, CDCl$_3$) spectrum for 5a

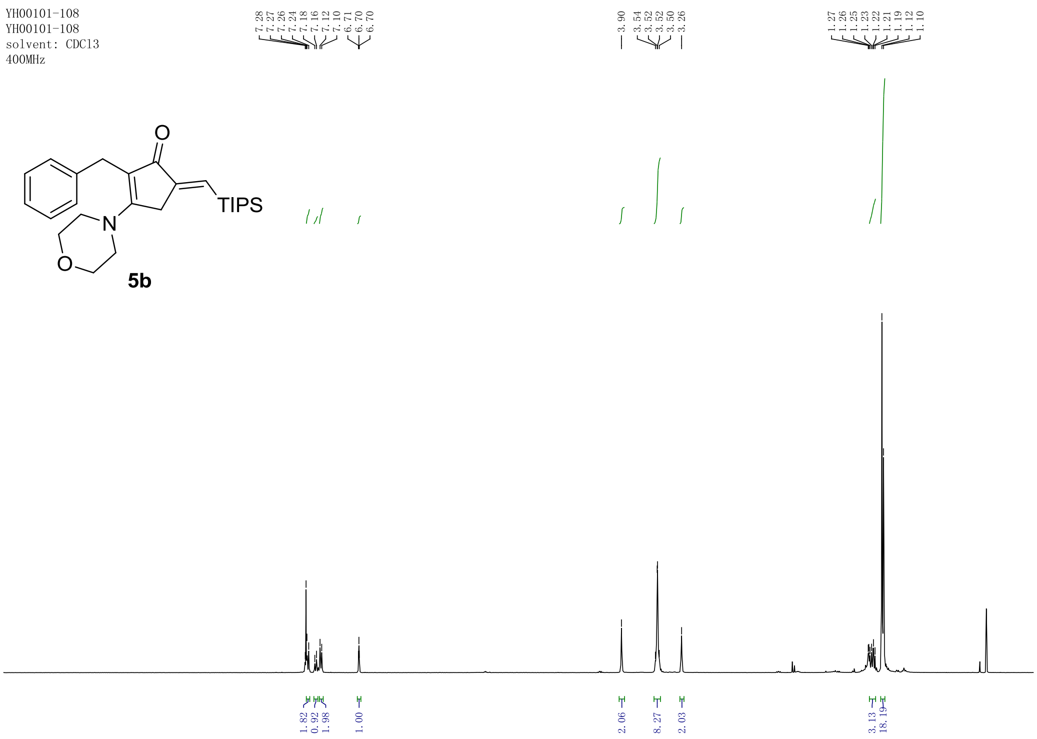

Supplementary Figure 73. ^{1}H NMR (400MHz, $CDCl_3$) spectrum for 5b

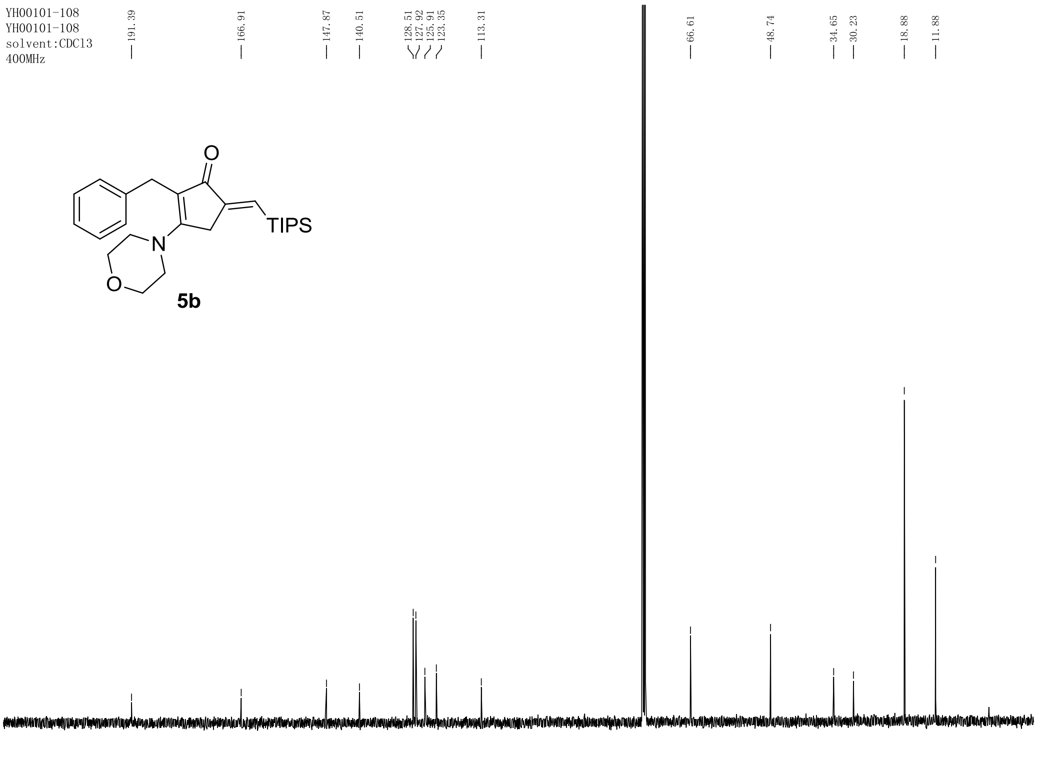

Supplementary Figure 74. ^{13}C NMR (101MHz, $CDCl_3$) spectrum for 5b

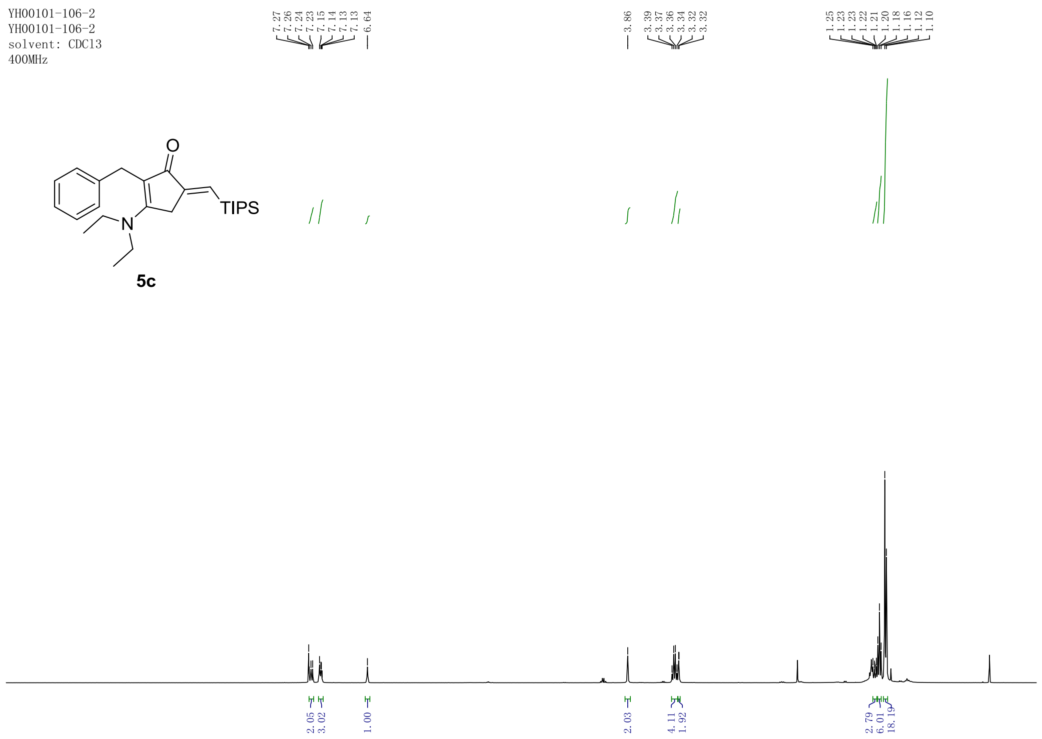

Supplementary Figure 75. ^{1}H NMR (400MHz, $CDCl_3$) spectrum for 5c

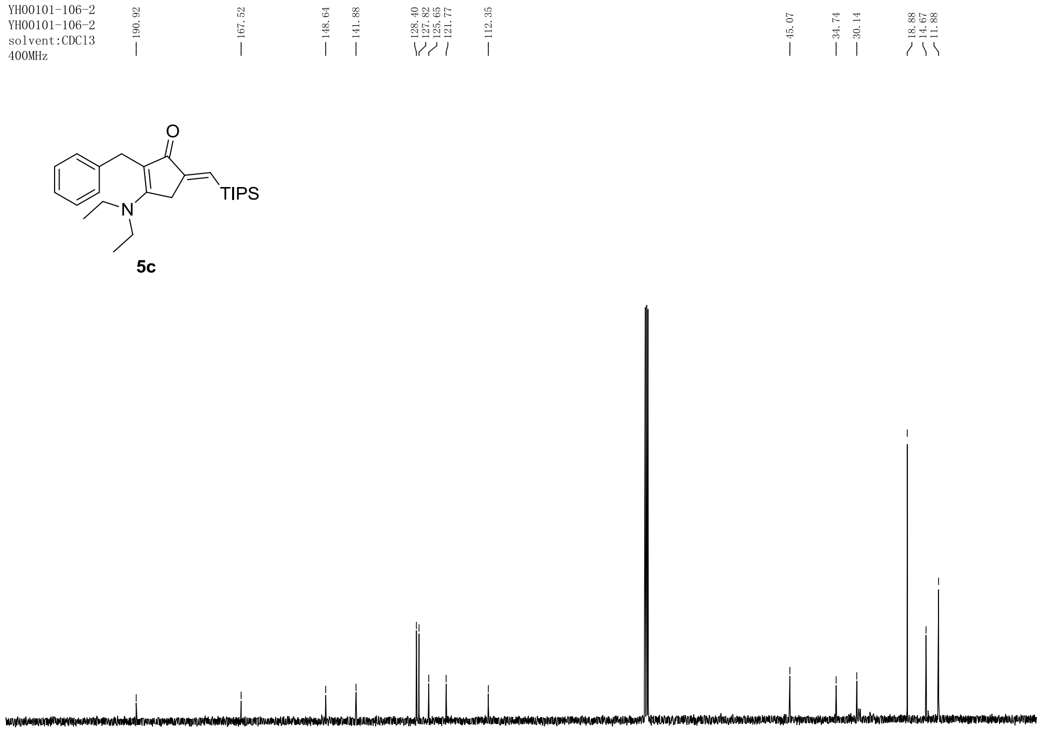

Supplementary Figure 76. ^{13}C NMR (101MHz, $CDCl_3$) spectrum for 5c

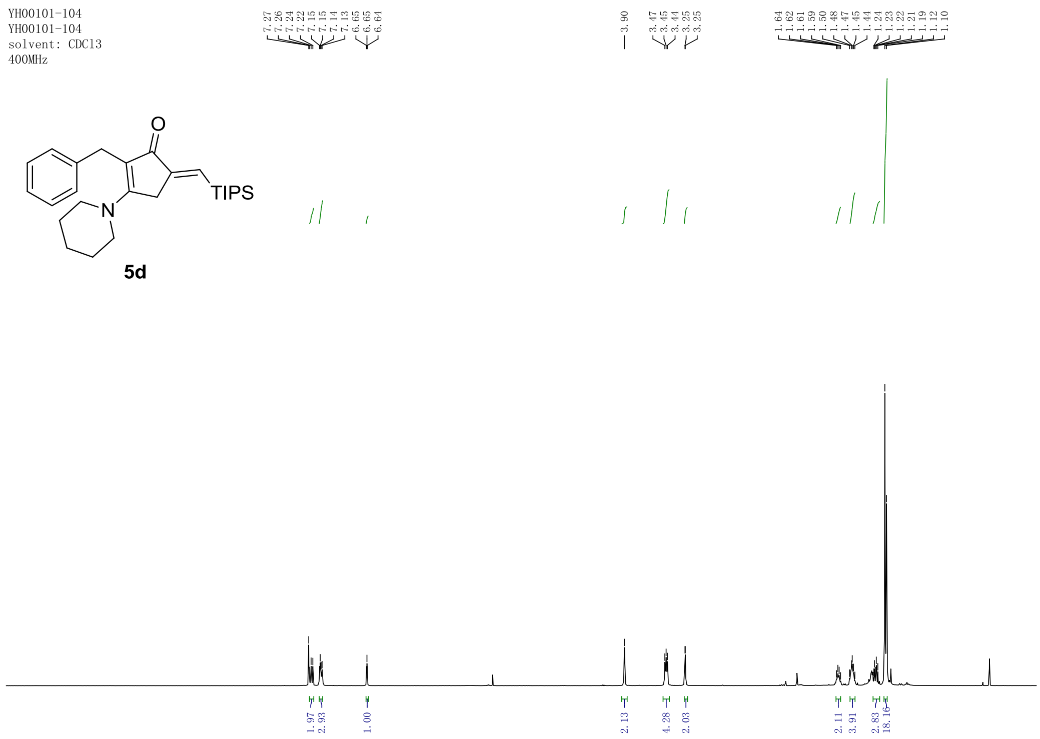

Supplementary Figure 77. ^{1}H NMR (400MHz, $CDCl_3$) spectrum for 5d

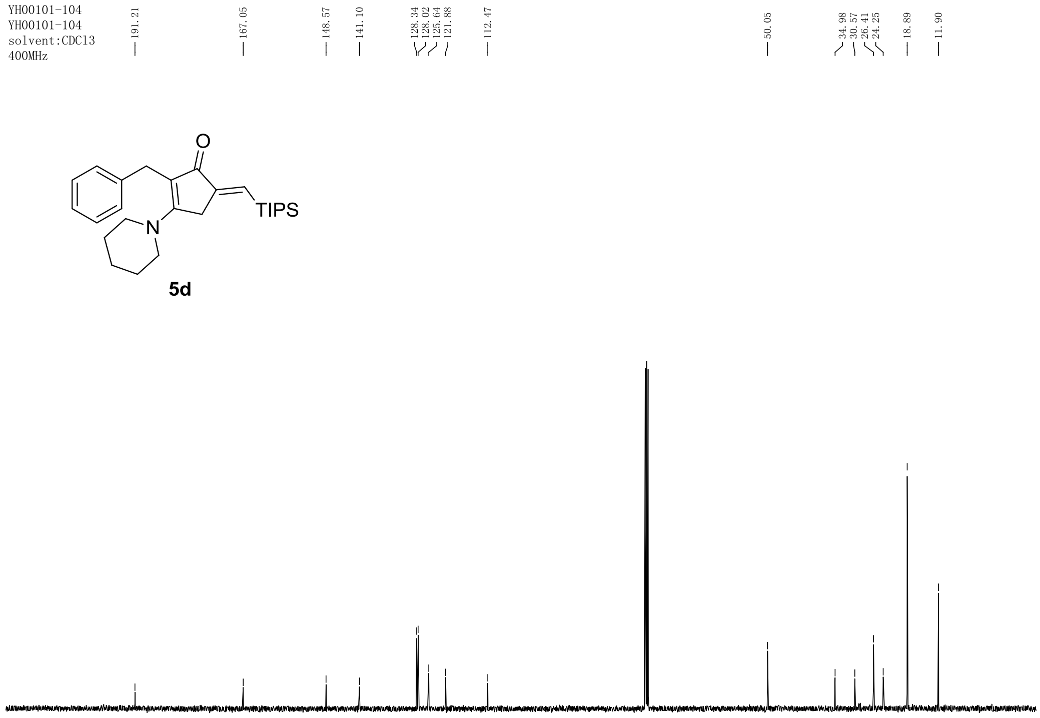

Supplementary Figure 78. ^{13}C NMR (101MHz, $CDCl_3$) spectrum for 5d

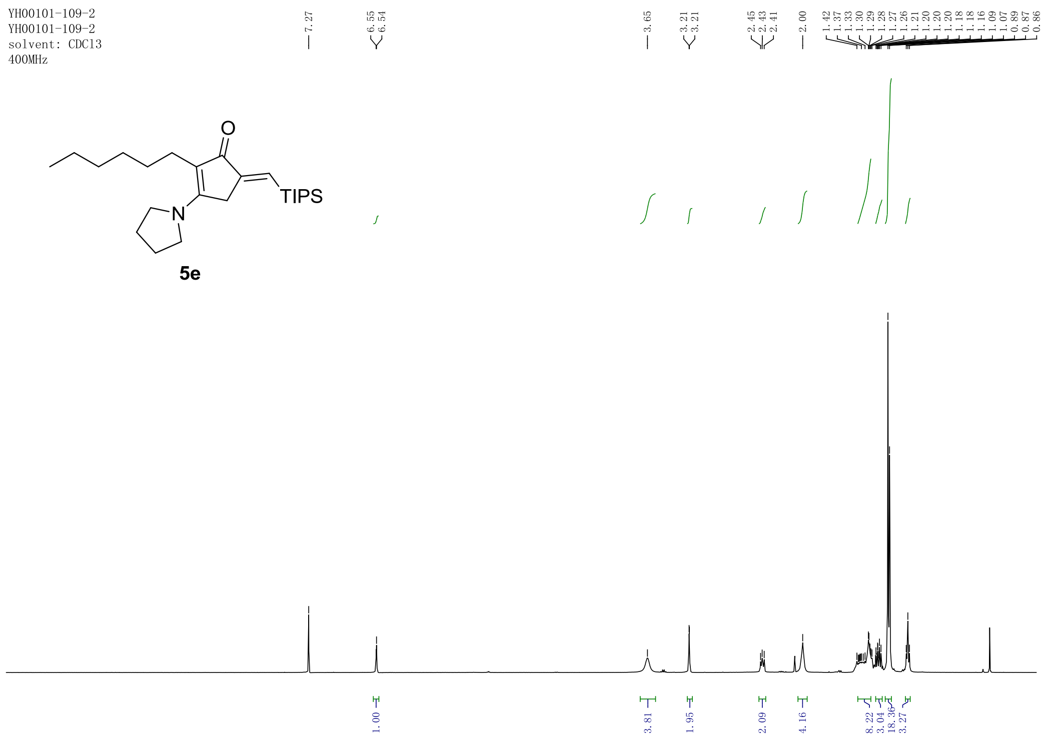

Supplementary Figure 79. ^{1}H NMR (400MHz, $CDCl_3$) spectrum for 5e

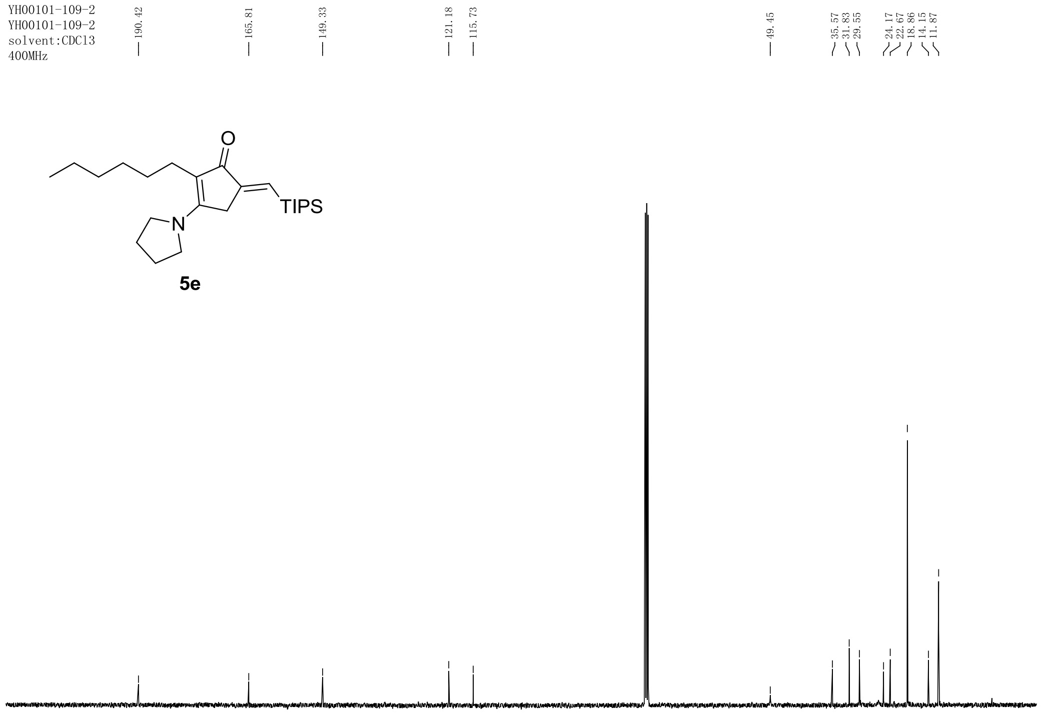

Supplementary Figure 80. ^{13}C NMR (101MHz, $CDCl_3$) spectrum for 5e

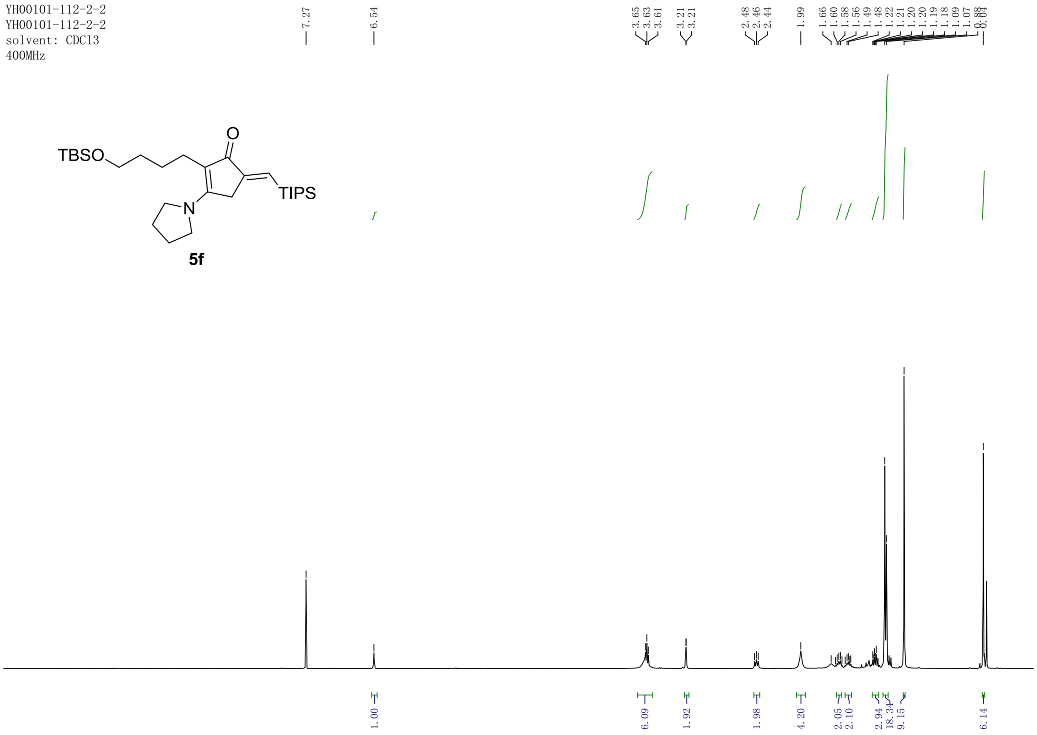

Supplementary Figure 81. ^{1}H NMR (400MHz, CDCl₃) spectrum for 5f

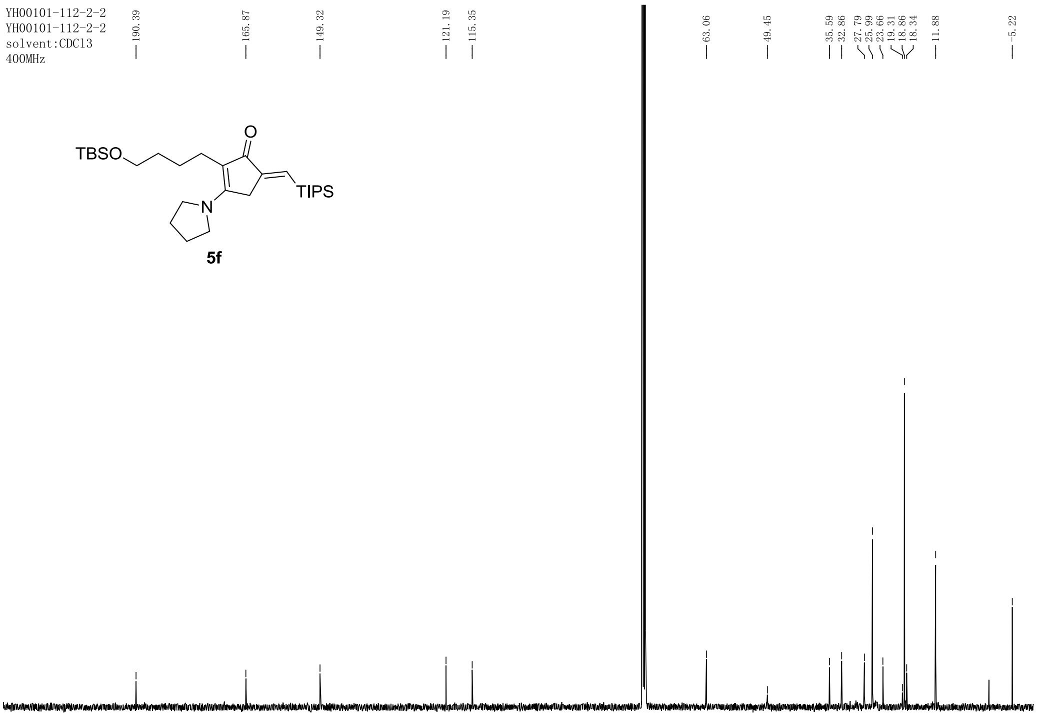

Supplementary Figure 82. ^{13}C NMR (101MHz, CDCl₃) spectrum for 5f

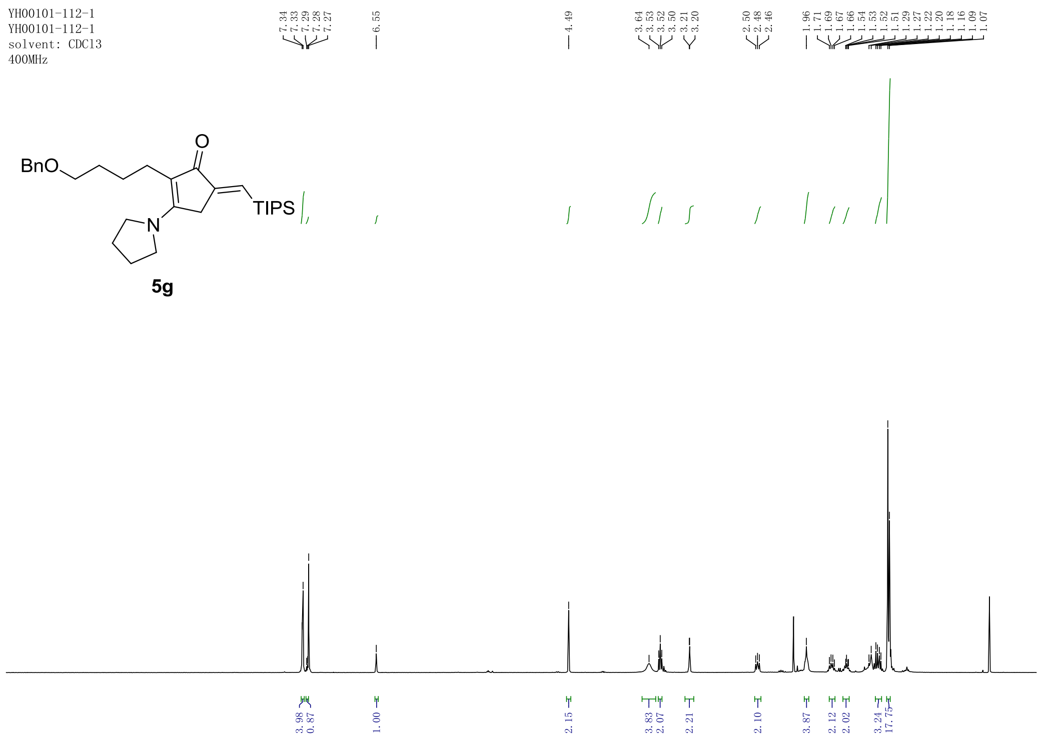

Supplementary Figure 83. ^{1}H NMR (400MHz, $CDCl_3$) spectrum for 5g

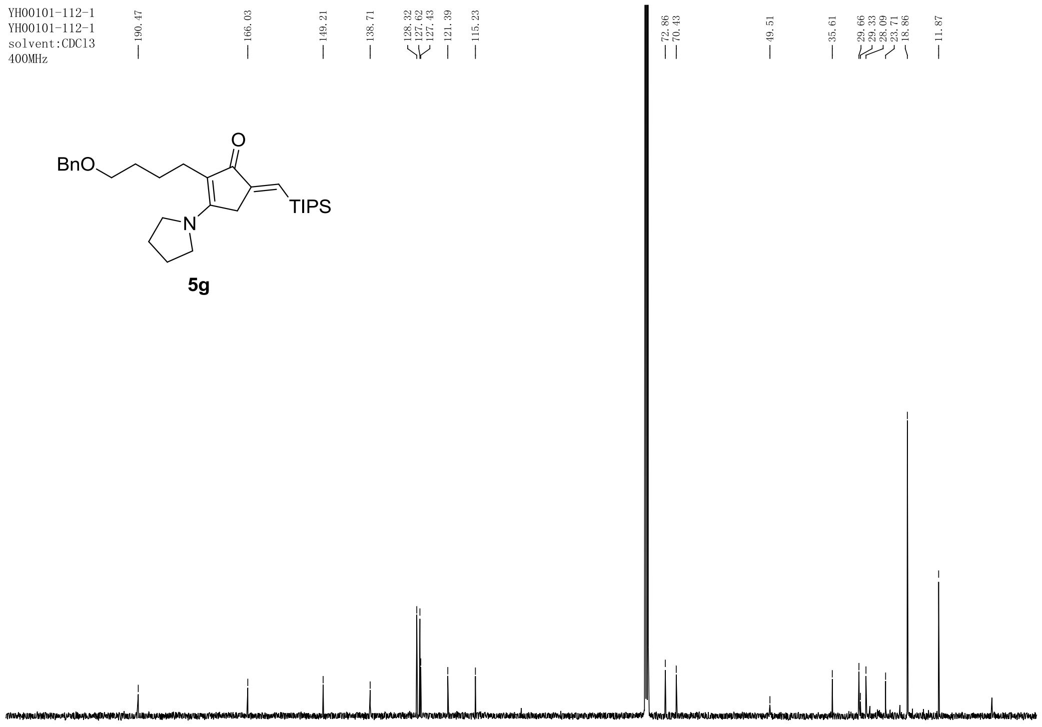

Supplementary Figure 84. ^{13}C NMR (101MHz, $CDCl_3$) spectrum for 5g

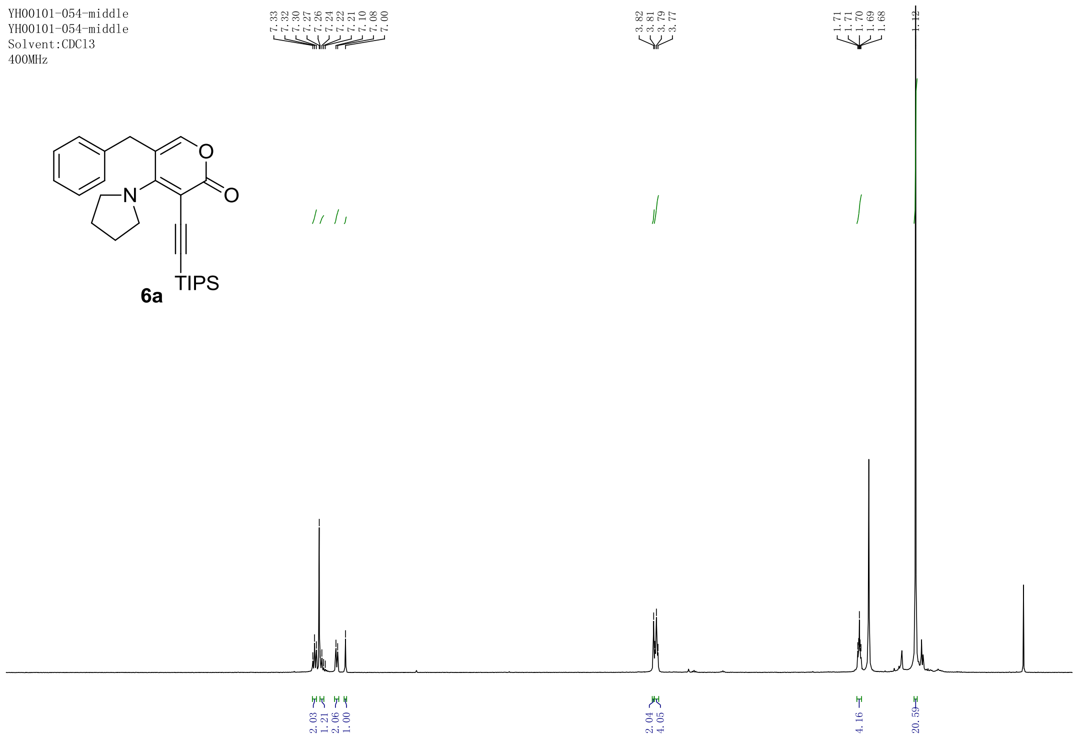

Supplementary Figure 85. ^{1}H NMR (400MHz, $CDCl_3$) spectrum for 6a

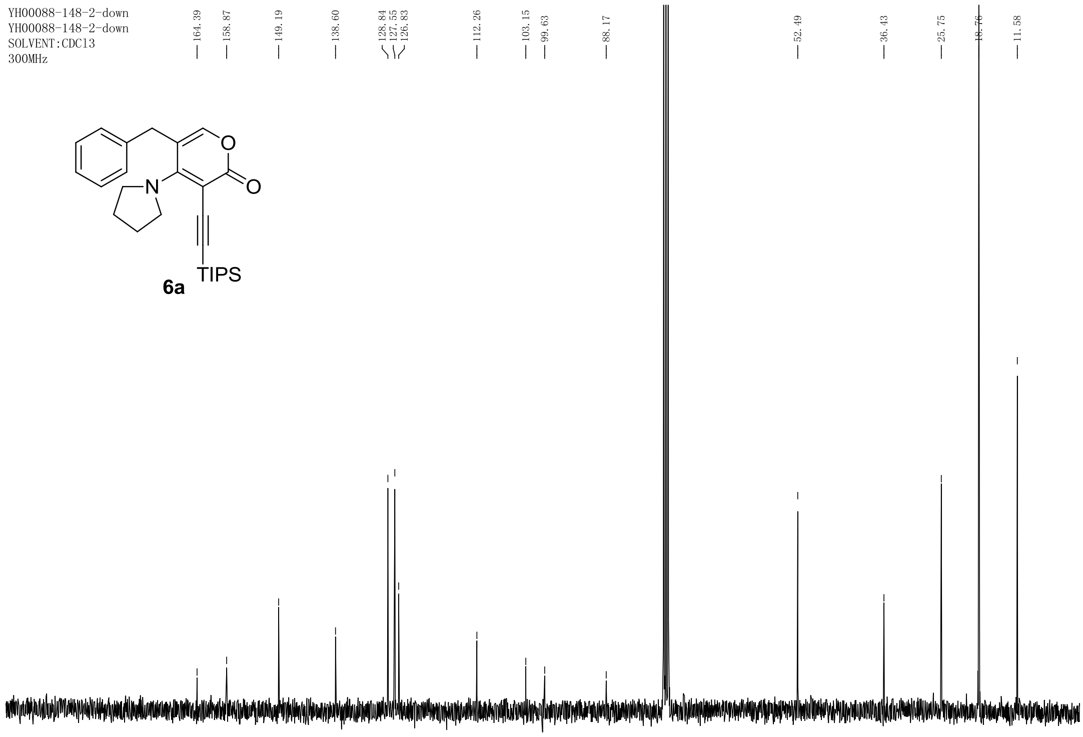

Supplementary Figure 86. ^{13}C NMR (75MHz, $CDCl_3$) spectrum for 6a

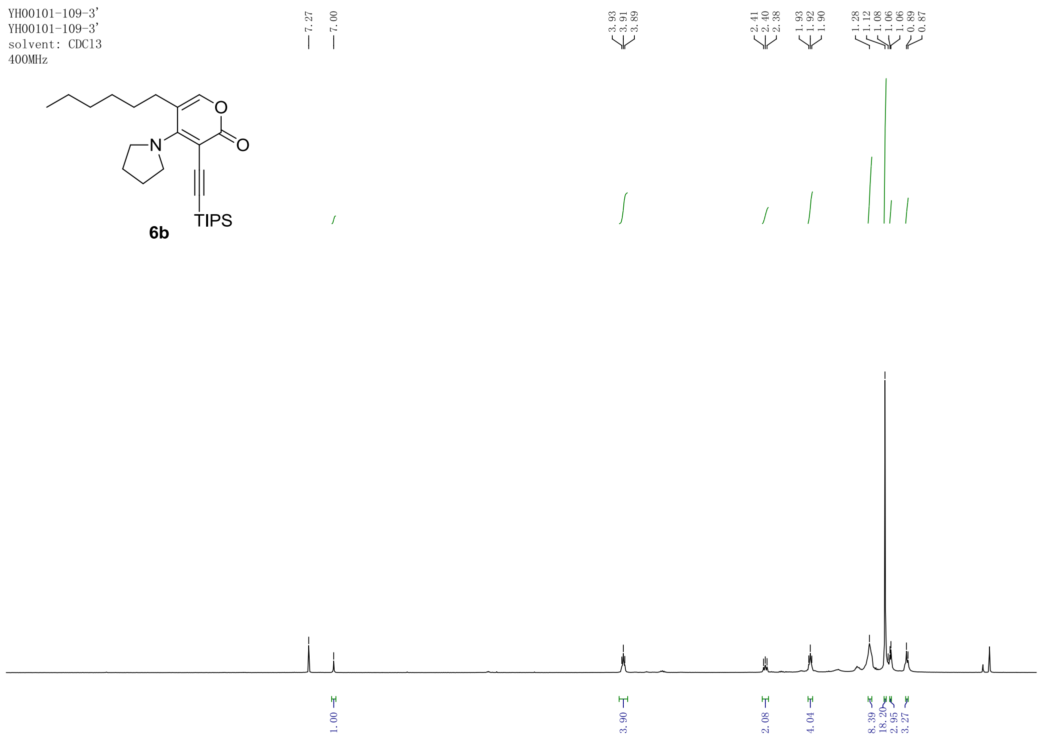

Supplementary Figure 87. ^{1}H **NMR (400MHz,** $CDCl_3$**) spectrum for 6b**

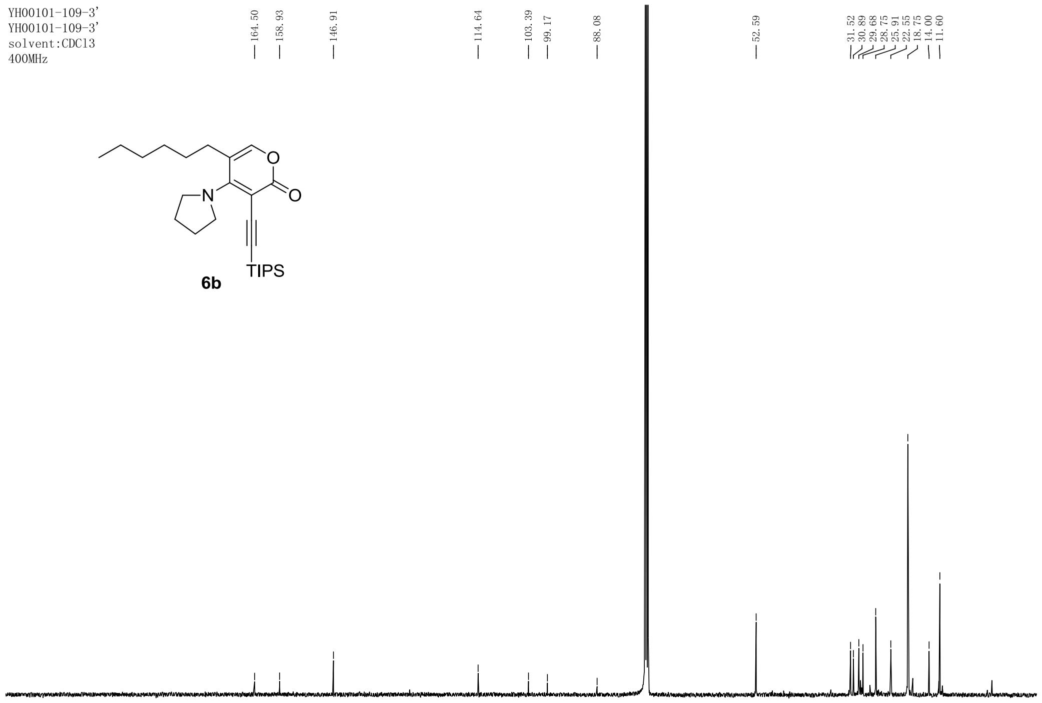

Supplementary Figure 88. ^{13}C **NMR (101MHz,** $CDCl_3$**) spectrum for 6b**

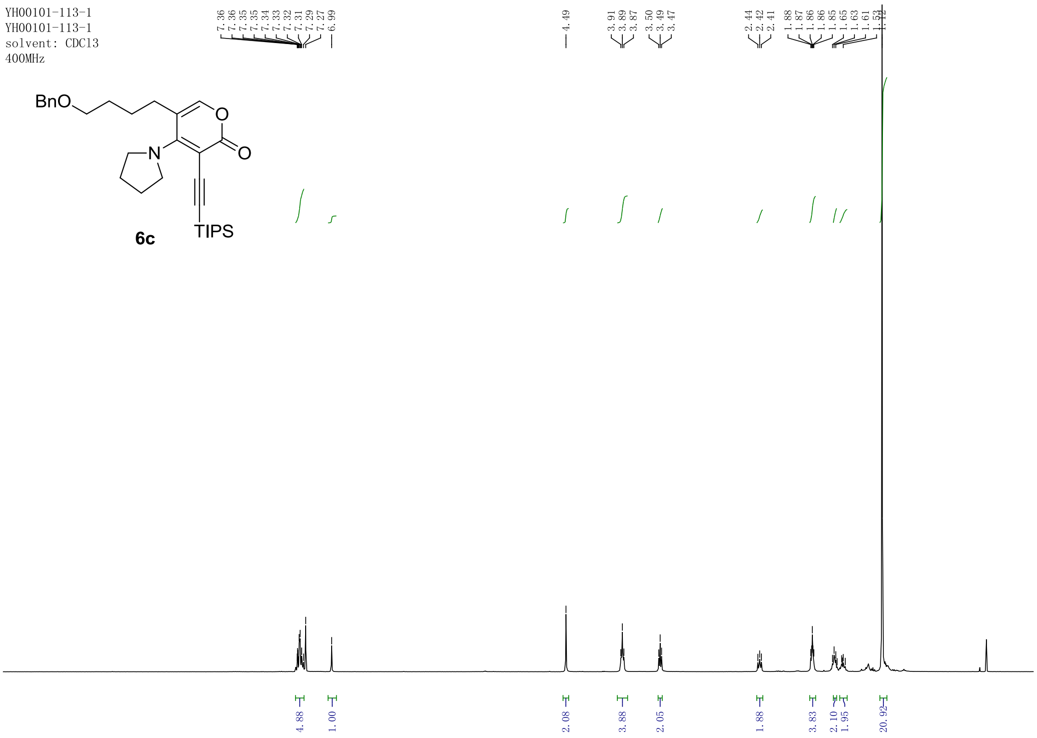

Supplementary Figure 89. ^{1}H NMR (400MHz, $CDCl_3$) spectrum for 6c

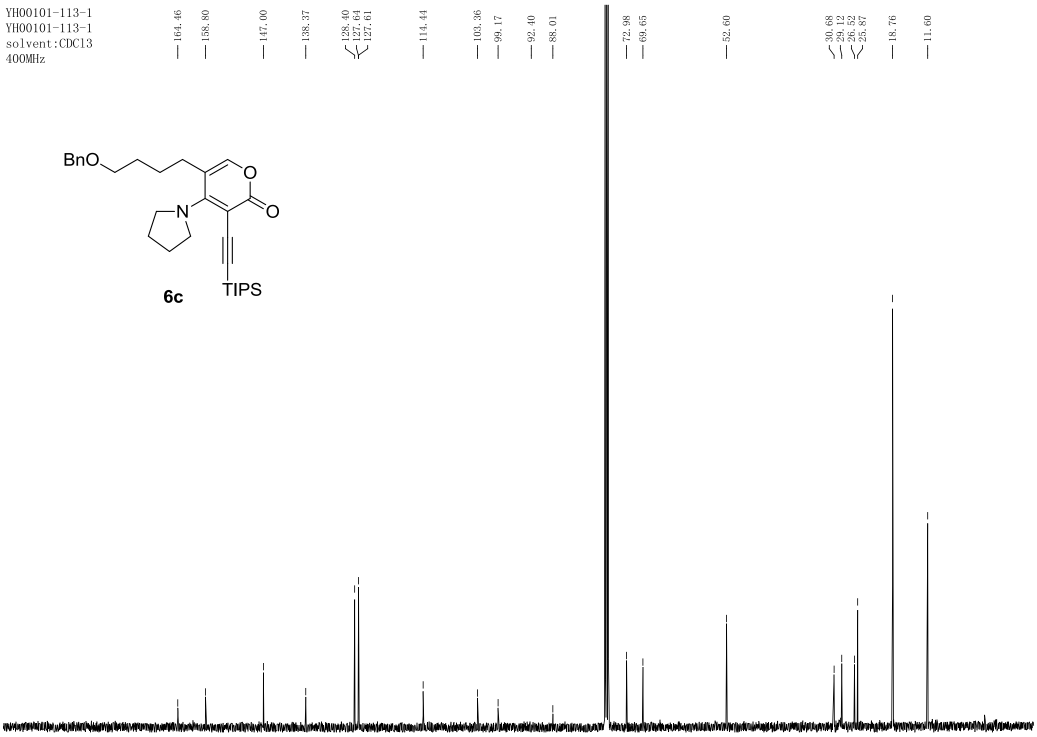

Supplementary Figure 90. ^{13}C NMR (101MHz, $CDCl_3$) spectrum for 6c

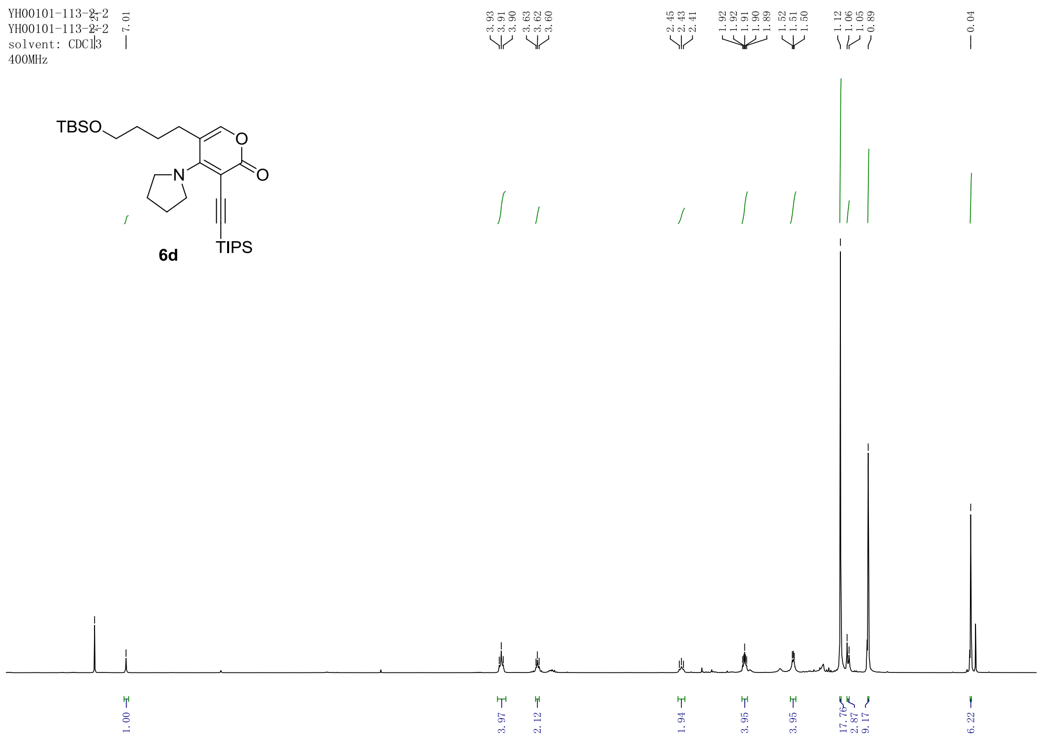

Supplementary Figure 91. ^{1}H NMR (400MHz, $CDCl_3$) spectrum for 6d

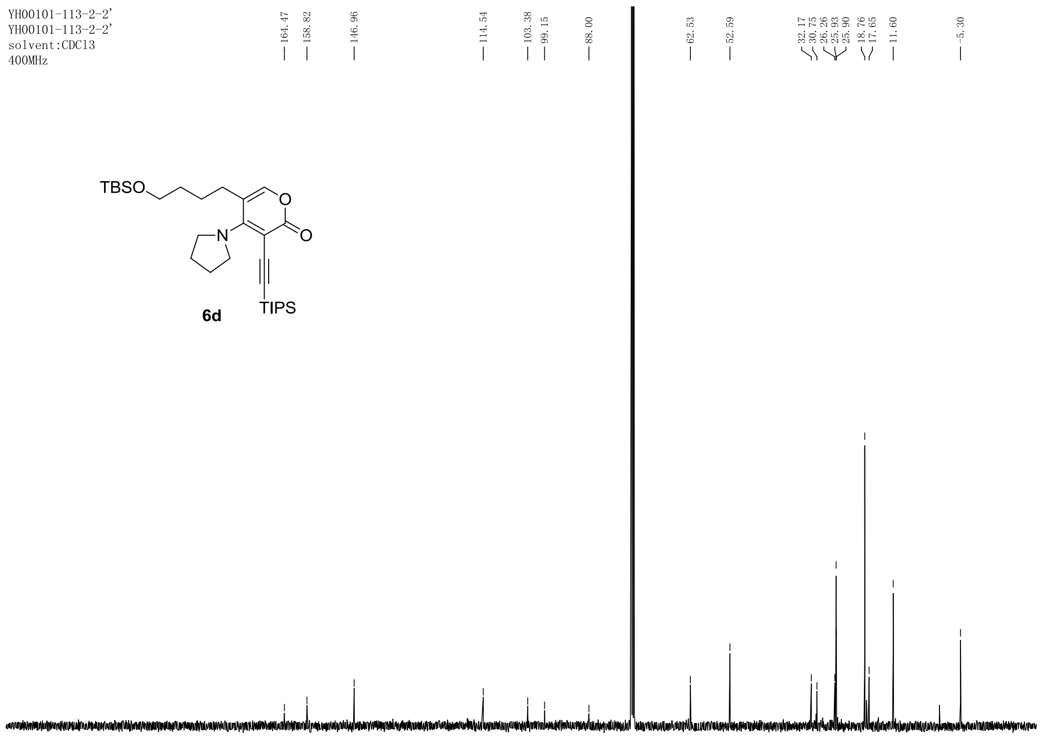

Supplementary Figure 92. ^{13}C NMR (101MHz, $CDCl_3$) spectrum for 6d

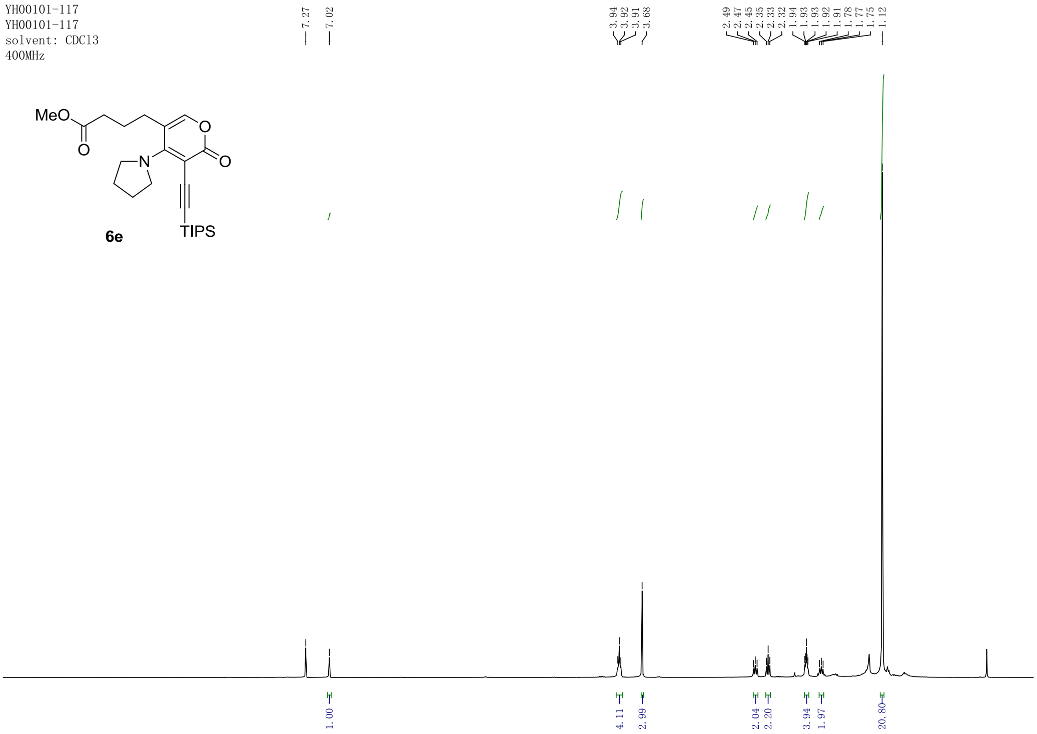

Supplementary Figure 93. ^{1}H NMR (400MHz, $CDCl_3$) spectrum for 6e

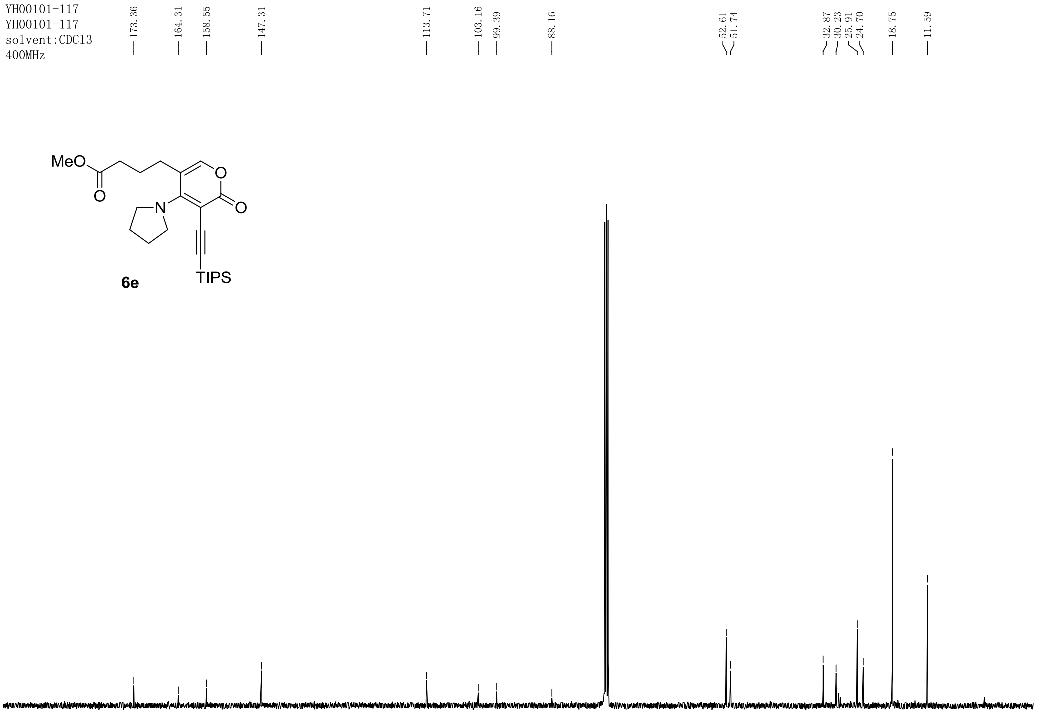

Supplementary Figure 94. ^{13}C NMR (101MHz, $CDCl_3$) spectrum for 6e

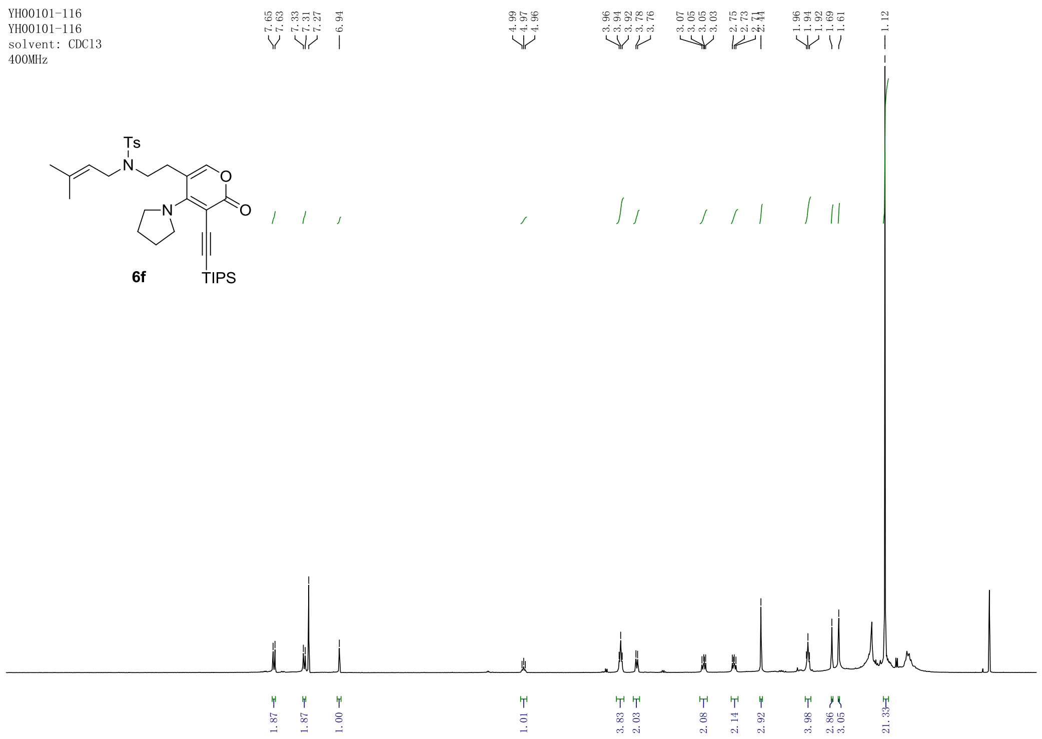

Supplementary Figure 95. ^{1}H NMR (400MHz, $CDCl_3$) spectrum for 6f

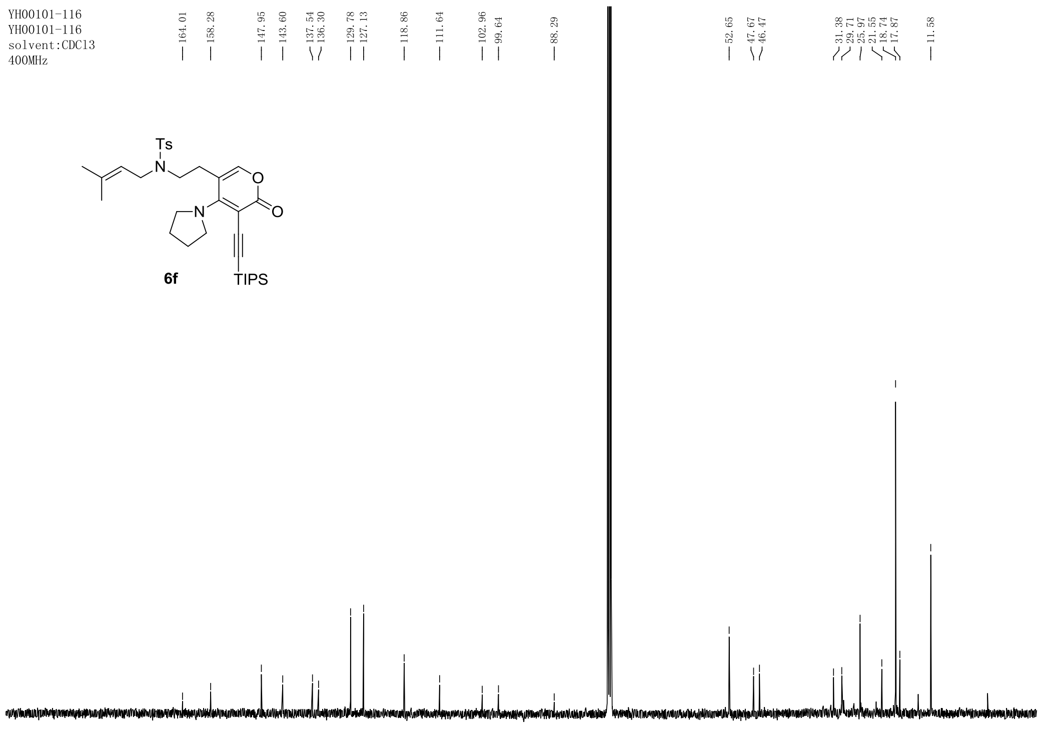

Supplementary Figure 96. ^{13}C NMR (101MHz, $CDCl_3$) spectrum for 6f

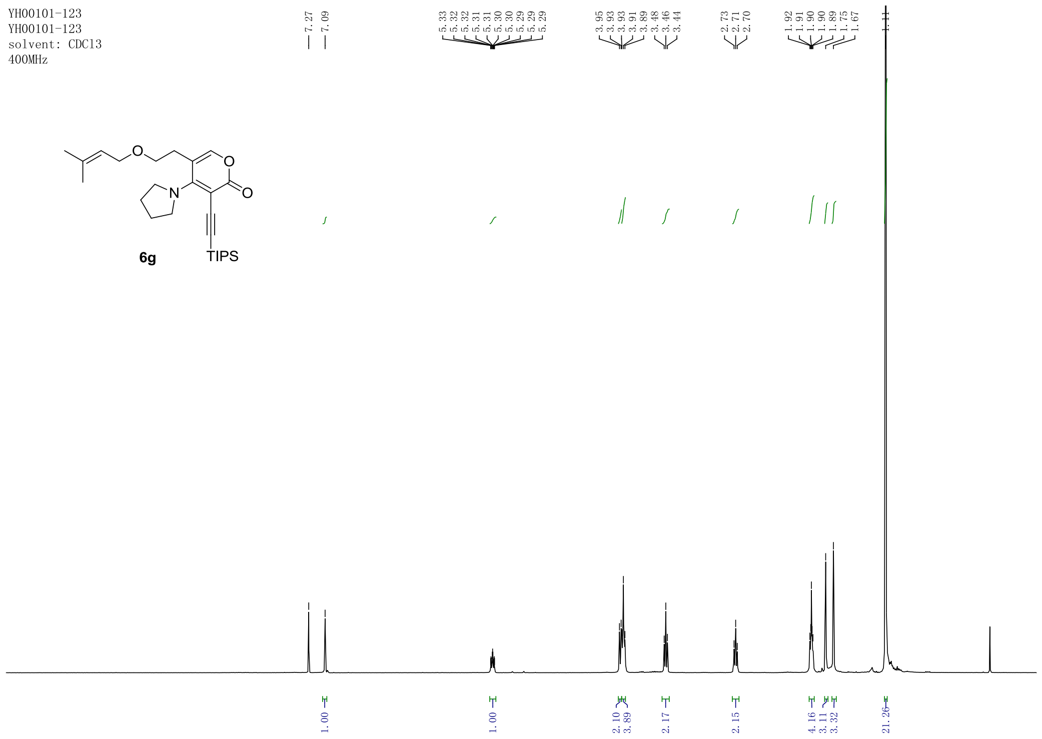

Supplementary Figure 97. ^{1}H NMR (400MHz, $CDCl_3$) spectrum for 6g

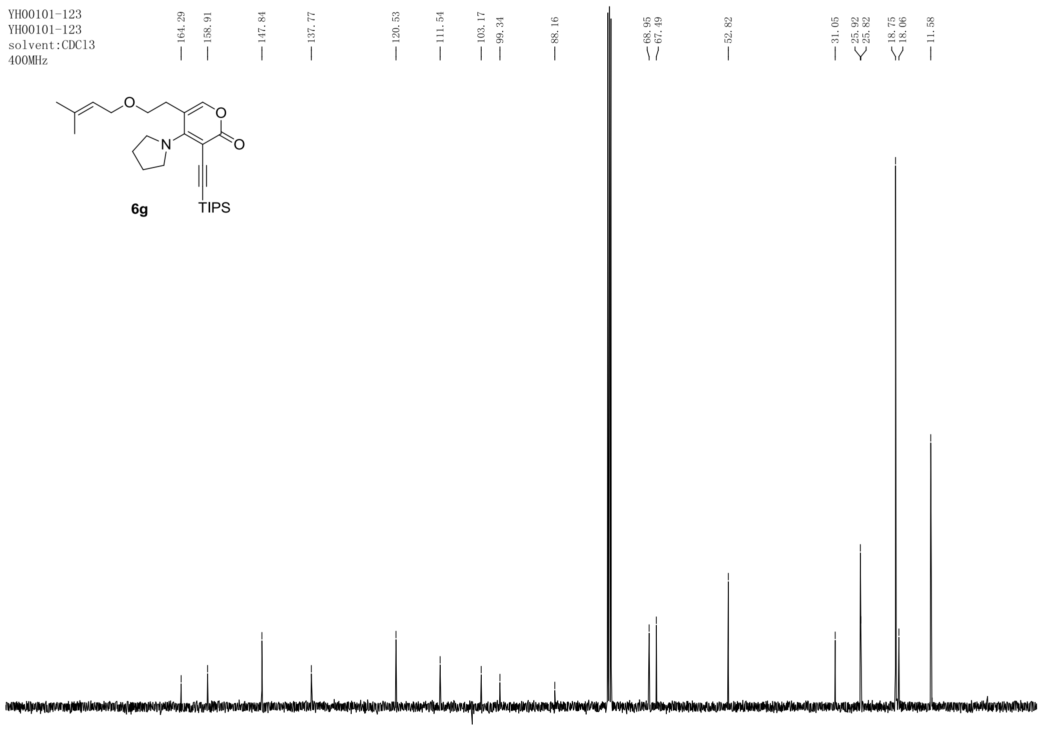

Supplementary Figure 98. ^{13}C NMR (101MHz, $CDCl_3$) spectrum for 6g

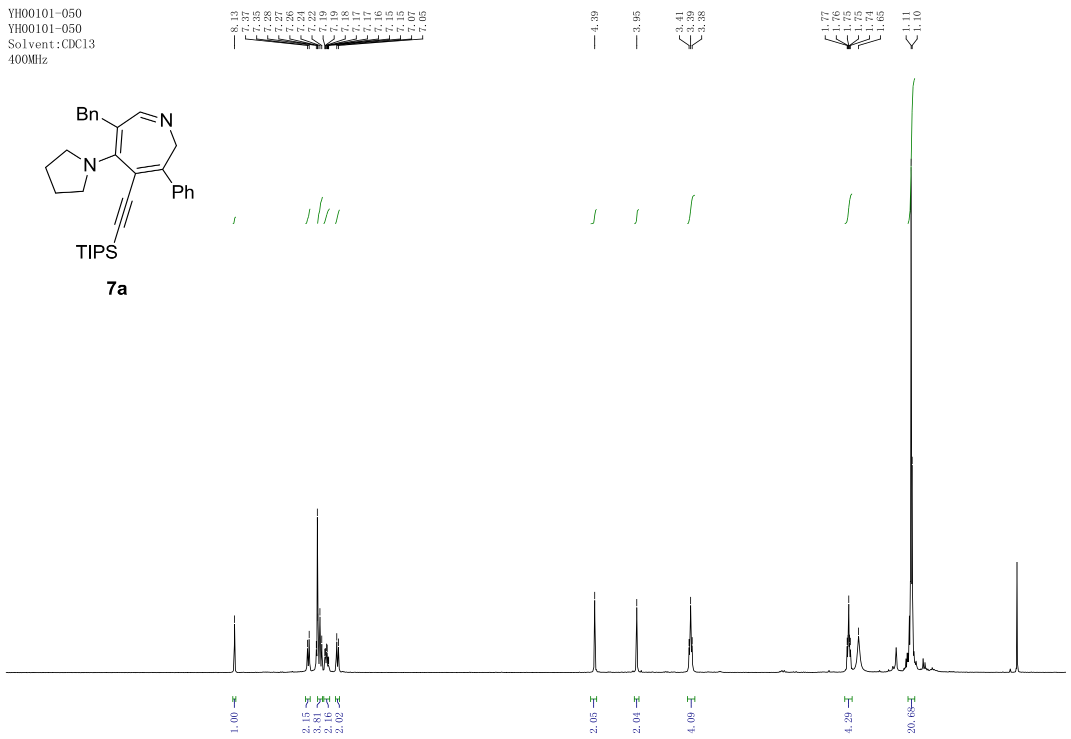

Supplementary Figure 99. ^{1}H NMR (400MHz, $CDCl_3$) spectrum for 7a

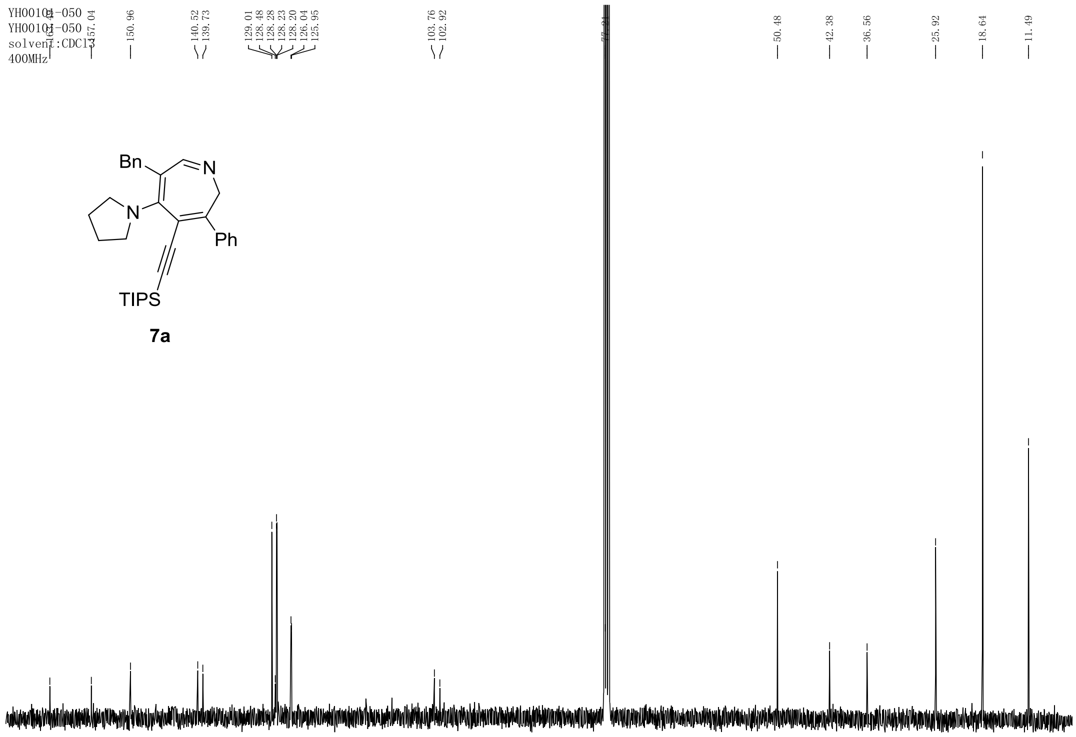

Supplementary Figure 100. ^{13}C NMR (101MHz, $CDCl_3$) spectrum for 7a

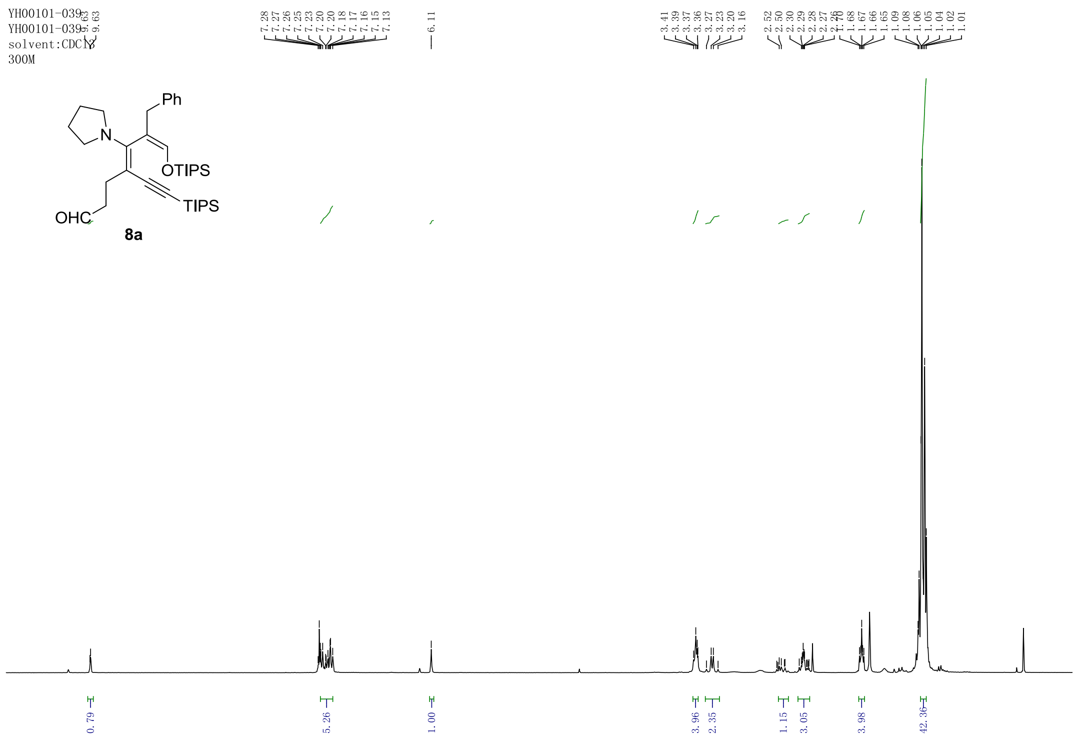

Supplementary Figure 101. ^{1}**H NMR (300MHz, CDCl$_3$) spectrum for 8a**

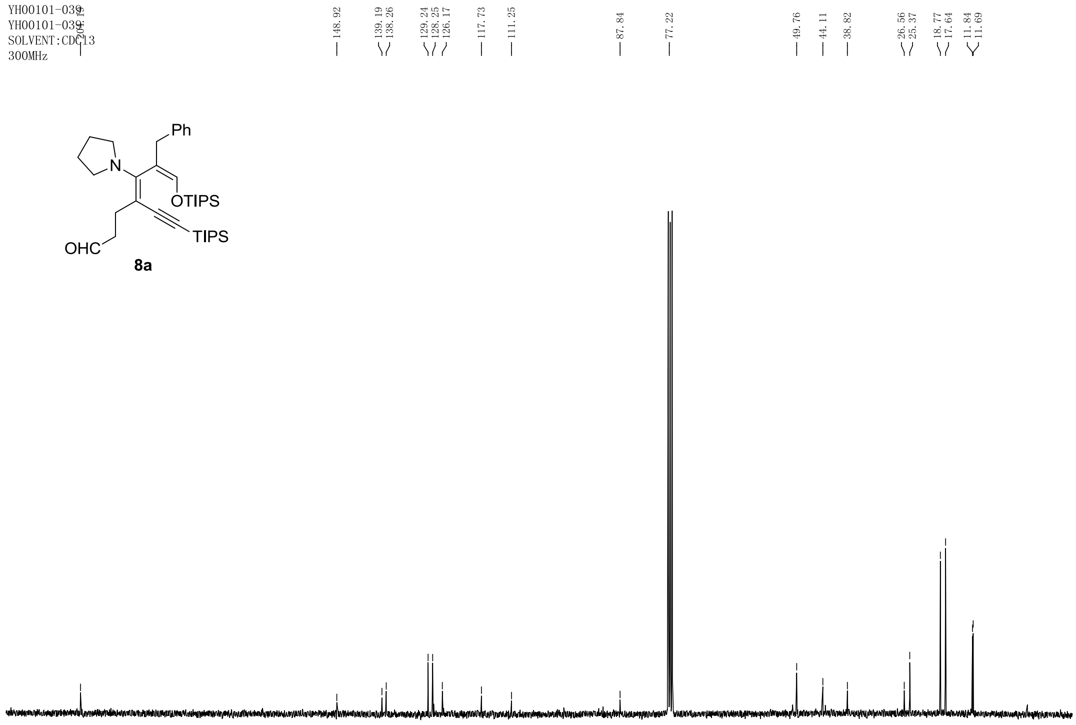

Supplementary Figure 102. ^{13}**C NMR (75MHz, CDCl$_3$) spectrum for 8a**

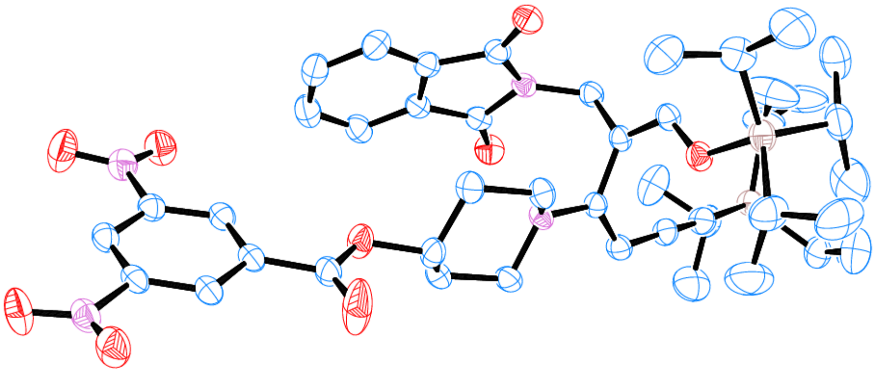

Supplementary Figure 103. Ortep structure for 3in

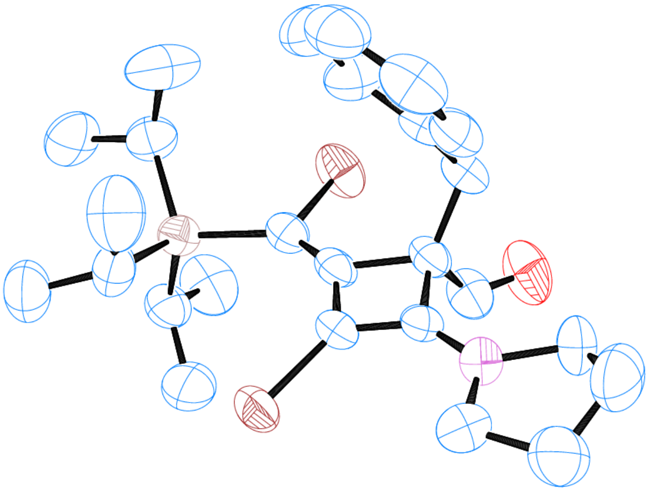

Supplementary Figure 104. Ortep structure for 4a

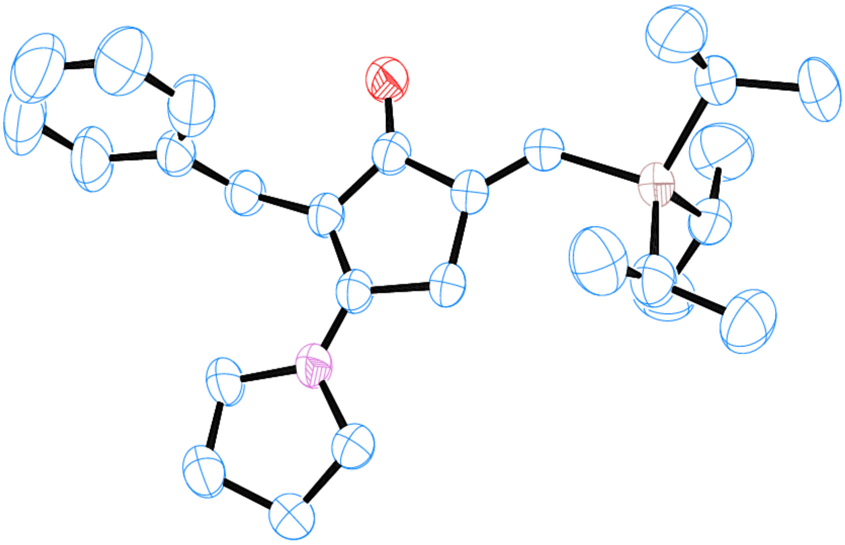

Supplementary Figure 105. Ortep structure for 5a

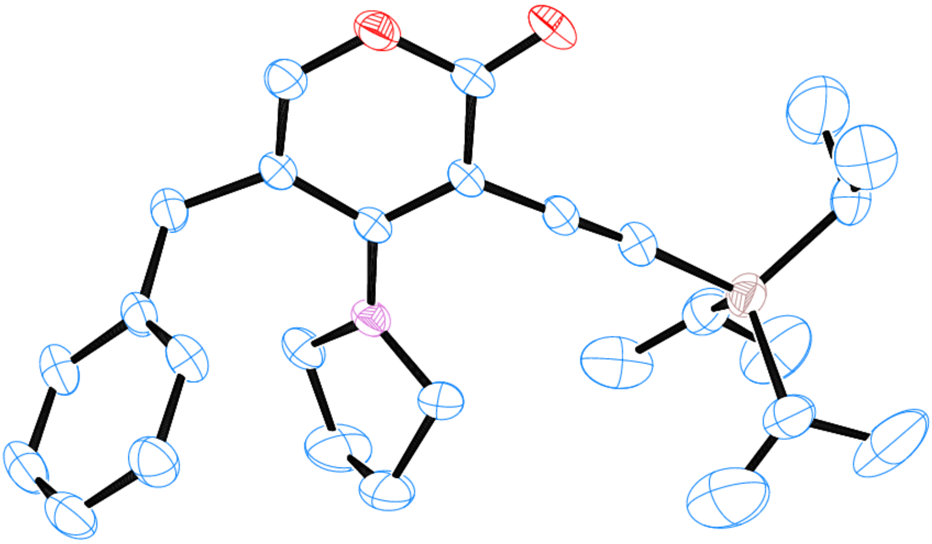

Supplementary Figure 106. Ortep structure for 6a

Supplementary methods

Analytical data of the products:

(1) 1-((1Z,3E)-2-benzyl-6-(triisopropylsilyl)-1-((triisopropylsilyl)oxy)hexa-1,3-dien-5-yn-3-yl)pyrrolidine **3aa** (YH00088-1)

The title compound was prepared according to the general procedure for the synthesis of **3** (87%). **^{1}H NMR** (400 MHz, $CDCl_3$): δ 7.24 - 7.19 (m, 4H), 7.17-7.13 (m, 1H), 6.27 (s, 1H), 4.07 (s, 1H), 3.92 (dd, *J* = 14.0, 1.3 Hz, 1H), 3.32 (d, *J* = 14.1 Hz, 1H), 2.97 (s, 2H), 2.67 (s, 2H), 1.62 (s, 2H), 1.52 (s, 2H), 1.12-1.04 (m, 42H). ppm. **^{13}C NMR** (101 MHz, $CDCl_3$): δ 154.87, 140.44, 137.02, 129.16, 127.98, 125.82, 118.61, 110.00, 87.02, 72.13, 47.41, 37.85, 25.29, 18.88, 17.69, 11.87, 11.76. ppm. **HRMS** (**APCI-TOF**) calculated for C35H59NOSi2H(+): 566.4213; Found: 566.4208.

(2) 1-((1Z,3E)-2-(4-bromobenzyl)-6-(triisopropylsilyl)-1-((triisopropylsilyl)oxy)hexa-1,3-dien-5-yn-3-yl)pyrrolidine **3ab** (YH00088-2)

The title compound was prepared according to the general procedure for the synthesis of **3** (89%). **^{1}H NMR** (500 MHz, $CDCl_3$): δ 7.35 (d, *J* = 8.2 Hz, 2H), 7.10 (d, *J* = 8.2 Hz, 2H), 6.27 (s, 1H), 4.07 (s, 1H), 3.90 (d, *J* = 14.1 Hz, 1H), 3.25 (d, *J* = 14.2 Hz, 1H), 2.99 (s, 2H), 2.69 (s, 2H), 1.65 (s, 2H), 1.55 (s, 2H), 1.10-1.03 (m, 42H). ppm. **^{13}C NMR** (126 MHz, $CDCl_3$): δ 154.34, 139.48, 137.38, 131.01, 130.93, 119.65, 118.07, 109.80, 87.33, 72.73, 47.47, 37.16, 25.26, 18.84, 17.66, 11.91, 11.77. ppm. **HRMS** (**APCI-TOF**) calculated for C35H59BrNOSi2H(+): 644.3319; Found: 644.3313.

(3) 1-((1Z,3E)-2-(4-iodobenzyl)-6-(triisopropylsilyl)-1-((triisopropylsilyl)oxy)hexa-1,3-dien-5-yn-3-yl)pyrrolidine **3ac** (YH00088-3)

The title compound was prepared according to the general procedure for the synthesis of **3** (90%). **^{1}H NMR** (300 MHz, $CDCl_3$): δ 7.55 (d, *J* = 8.2 Hz, 2H), 6.98 (d, *J* = 8.2 Hz, 2H), 6.28 (s, 1H), 4.06 (s, 1H), 3.89 (d, *J* = 13.8 Hz, 1H), 3.23 (d, *J* = 14.1 Hz, 1H), 2.97 (s, 2H), 2.68 (s, 2H), 1.63 (s, 2H), 1.61 (s, 2H), 1.09-1.03 (m, 42H). ppm. **^{13}C NMR** (75 MHz, $CDCl_3$): δ 154.40, 140.16, 137.34, 137.01, 131.29, 118.00, 109.80, 90.97, 87.32, 72.33, 47.48, 37.24, 25.30, 18.87, 17.69, 11.84, 11.73. ppm. **HRMS** (**APCI-TOF**) calculated for C35H59INOSi2H(+): 692.3180; Found: 692.3172.

(4) 1-((1Z,3E)-2-(naphthalen-2-ylmethyl)-6-(triisopropylsilyl)-1-((triisopropylsilyl)oxy)hexa-1,3-dien-5-yn-3-yl)pyrrolidine **3ad** (YH00088-4)

The title compound was prepared according to the general procedure for the synthesis of **3** (84%). **^{1}H NMR** (300 MHz, $CDCl_3$): δ 7.80-7.74 (m, 3H), 7.66 (s, 1H), 7.44-7.38 (m, 3H), 6.33 (s, 1H), 4.08 (d, *J* = 13.6 Hz, 2H), 3.49 (d, *J* = 14.2 Hz, 1H), 2.97 (s, 2H), 2.67 (s, 2H), 1.54 (s, 2H), 1.44 (s, 2H), 1.11-1.03 (m, 42H). ppm. **^{13}C NMR** (75 MHz, $CDCl_3$): δ 154.95, 137.96, 137.37, 133.58, 132.04, 128.04, 127.54, 127.40, 127.19, 125.67, 125.47, 125.02, 118.37, 110.01, 87.20, 72.19, 47.48, 38.03, 25.22, 18.92, 17.70, 11.87, 11.79. ppm. **HRMS** (**APCI-TOF**) calculated for C39H61NOSi2H(+): 616.4370; Found: 616.4365.

(5) 1-((1Z,3E)-2-(thianthren-1-ylmethyl)-6-(triisopropylsilyl)-1-((triisopropylsilyl)oxy)hexa-1,3-dien-5-yn-3-yl)pyrrolidine **3ae** (YH00088-5)

The title compound was prepared according to the general procedure for the synthesis of **3** (96%). **^{1}H NMR** (300 MHz, $CDCl_3$): δ 7.54-7.47 (m, 2H), 7.35 (d, *J* = 7.5 Hz, 1H), 7.24 (d, *J* = 5.2 Hz, 3H), 7.11 (t, *J* = 7.6 Hz, 1H), 6.50 (s, 1H), 4.12 (d, *J* = 13.4 Hz, 1H), 4.06 (s, 1H), 3.72 (d, *J* = 14.0 Hz, 1H), 2.90 (s, 2H), 2.59 (s, 2H), 1.50 (s, 4H), 1.08 (dd, *J* = 15.4, 5.0 Hz, 42H). ppm. **^{13}C NMR** (75 MHz, $CDCl_3$): δ 154.17, 139.78, 138.74, 136.52, 135.83, 135.68, 135.20, 129.80, 129.02, 128.49, 127.67, 127.45, 126.90, 116.18, 110.02, 87.24, 72.52, 47.43, 35.99, 25.24, 18.91, 17.72, 11.86, 11.76. ppm. **HRMS** (**APCI-TOF**) calculated for C41H61NOS2Si2H(+): 704.3811; Found: 704.3804.

(6) 1-((1Z,3E)-2-(4-(trifluoromethyl)benzyl)-6-(triisopropylsilyl)-1-((triisopropylsilyl)oxy)hexa-1,3-dien-5-yn-3-yl)pyrrolidine **3af** (YH00088-6)

The title compound was prepared according to the general procedure for the synthesis of **3** (91%). **^{1}H NMR** (400 MHz, $CDCl_3$): δ 7.49 (d, *J* = 8.0 Hz, 2H), 7.34 (d, *J* = 8.0 Hz, 2H), 6.31 (s, 1H), 4.08 (s, 1H), 4.03 (d, *J* = 13.9 Hz, 1H), 3.34 (d, *J* = 14.0 Hz, 1H), 2.96 (s, 2H), 2.64 (s, 2H), 1.62 (s, 2H), 1.52 (s, 2H), 1.13 – 1.02 (m, 42H). ppm. **^{13}C NMR** (101 MHz, $CDCl_3$): δ 154.16, 144.65, 137.60, 129.44, 128.56(q, 32 Hz), 124.89(q, 4 Hz), 124.41(q, 270 Hz), 117.70, 109.73, 87.48, 72.58, 47.46, 37.58, 25.24, 18.87, 17.67, 11.86, 11.74. ppm. **HRMS** (**APCI-TOF**) calculated for C36H58F3NOSi2H(+): 634.4087; Found: 634.4081.

(7) 1-((3E,5Z)-9-((tert-butyldimethylsilyl)oxy)-1-(triisopropylsilyl)-5-(((triisopropylsilyl)oxy)methylene)non-3-en-1-yn-4-yl)pyrrolidine **3ag** (YH00088-7)

The title compound was prepared according to the general procedure for the synthesis of **3** (89%). **^{1}H NMR** (300 MHz, $CDCl_3$): δ 6.21 (s, 1H), 4.03 (s, 1H), 3.61 (t, *J* = 6.3 Hz, 2H), 3.21 (s, 2H), 3.14 (s, 2H), 2.54 (t, *J* = 10.6 Hz, 1H), 2.15-2.04 (m, 1H), 1.83 (s, 4H), 1.11-1.04 (m, 46H), 0.89 (s, 9H), 0.04 (s, 6H). ppm. **^{13}C NMR** (126 MHz, $CDCl_3$): δ 155.82, 136.42, 118.33, 109.75, 86.60, 72.26, 63.21, 47.69, 32.97, 31.60, 25.98, 25.49, 25.11, 18.83, 18.75, 17.72, 11.91, 11.73, -5.27. ppm. **HRMS** (**APCI-TOF**) calculated for C38H75NO2Si3H(+): 662.5184; Found: 662.5179.

(8) (5E,6Z)-methyl-6-(pyrrolidin-1-yl)-9-(triisopropylsilyl)-5-(((triisopropylsilyl)oxy)methylene)non-6-en-8-ynoate **3ah** (YH00088-8)

The title compound was prepared according to the general procedure for the synthesis of **3** (78%). **^{1}H NMR** (500 MHz, $CDCl_3$): δ 6.24 (s, 1H), 4.05 (s, 1H), 3.66 (s, 3H), 3.22 (s, 2H), 3.13 (s, 2H), 2.58 (t, *J* = 9.6 Hz, 1H), 2.32 (t, *J* = 7.5 Hz, 2H), 2.10 (t, *J* = 15.0 Hz, 1H), 1.84 (s, 4H), 1.11-0.99 (m, 44H). ppm. **^{13}C NMR** (126 MHz, $CDCl_3$): δ 174.09, 155.20, 138.60, 137.09, 117.43, 109.61, 100.00, 86.85, 72.86, 51.34, 47.74, 34.12, 31.30, 25.45, 24.25, 18.80, 17.69, 11.93, 11.73. ppm. **HRMS** (**APCI-TOF**) calculated for C33H61NO3Si2H(+): 576.4268; Found: 576.4260.

(9) 2-((2Z,3E)-3-(pyrrolidin-1-yl)-6-(triisopropylsilyl)-2-(((triisopropylsilyl)oxy)methylene)hex-3-en-5-yn-1-yl)isoindoline-1,3-dione **3ai** (YH00088-9)

The title compound was prepared according to the general procedure for the synthesis of **3** (65%). **^{1}H NMR** (300 MHz, $CDCl_3$): δ 7.83 (dd, *J* = 5.3, 3.0 Hz, 2H), 7.69 (dd, *J* = 5.3, 3.1 Hz, 2H), 6.71 (s, 1H), 4.91 (d, *J* = 14.3 Hz, 1H), 4.22 (d, *J* = 14.4 Hz, 1H), 4.15 (s, 1H), 3.09 (s, 2H), 2.88 (s, 2H), 1.63 (d, *J* = 5.7 Hz, 4H), 1.16-1.04 (m, 42H). ppm. **^{13}C NMR** (75 MHz, $CDCl_3$): δ 167.95, 152.96, 142.82, 133.72, 132.19, 123.13, 113.01, 109.18, 87.51, 74.49, 47.66, 39.56, 25.31, 18.86, 17.65, 11.83, 11.70. ppm. **HRMS** (**APCI-TOF**) calculated for C37H58N2O3Si2H(+): 635.4064; Found: 635.4058.

(10)1-((3E,5Z,9Z)-1-(triisopropylsilyl)-5-(((triisopropylsilyl)oxy)methylene)dodeca-3,9-dien-1-yn-4-yl)pyrrolidine **3aj** (YH00088-10)

The title compound was prepared according to the general procedure for the synthesis of **3** (96%). **^{1}H NMR** (500 MHz, $CDCl_3$): δ 6.21 (s, 1H), 5.34 (d, *J* = 6.3 Hz, 2H), 4.04 (s, 1H), 3.22 (s, 2H), 3.14 (s, 2H), 2.56-2.51 (m, 1H), 2.16-2.08 (m, 1H), 2.08-2.01 (m, 4H), 1.84 (s, 4H), 1.11-1.01 (m, 44H), 0.96 (t, *J* = 7.5 Hz, 3H). ppm. **^{13}C NMR** (126 MHz, $CDCl_3$): δ 155.75, 136.41, 131.65, 129.10, 118.31, 109.73, 86.61, 72.36, 47.69, 31.48, 28.85, 27.32, 25.48, 20.53, 18.82, 17.72, 14.37, 11.92, 11.74. ppm. **HRMS** (**APCI-TOF**) calculated for C35H64NOSi2H(+): 572.4683; Found: 572.4680.

(11)1-((3E,5Z)-7-((3-methylbut-2-en-1-yl)oxy)-1-(triisopropylsilyl)-5-(((triisopropylsilyl)oxy)methylene)hept-3-en-1-yn-4-yl)pyrrolidine **3ak** (YH00088-11)

The title compound was prepared according to the general procedure for the synthesis of **3** (98%). **^{1}H NMR** (500 MHz, $CDCl_3$): δ 6.29 (s, 1H), 5.33-5.32 (m, 1H), 4.05 (s, 1H), 3.95-3.91 (m, 2H), 3.49 (t, 4.0 Hz, 2H), 3.19 (s, 4H), 2.85 (t, 4.0 Hz, 1H), 2.36-2.32 (m, 1H), 1.83 (d, *J* = 4.6 Hz, 4H), 1.73 (s, 3H), 1.66 (s, 3H), 1.12-1.00 (m, 42H). ppm. **^{13}C NMR** (126 MHz, $CDCl_3$): δ 155.01, 138.07, 121.52, 115.06, 109.72, 86.79, 72.69, 69.12, 67.03, 47.65, 31.91, 25.71, 25.50, 18.81, 17.91, 17.69, 11.95, 11.75. ppm. **HRMS** (**APCI-TOF**) calculated for C35H65NO2Si2H(+): 588.4632; Found: 588.4627

(12)1-((3E,5Z)-9-(benzyloxy)-1-(triisopropylsilyl)-5-(((triisopropylsilyl)oxy)methylene)non-3-en-1-yn-4-yl)pyrrolidine **3al** (YH00088-12)

The title compound was prepared according to the general procedure for the synthesis of **3** (89%). **^{1}H NMR** (400 MHz, $CDCl_3$): δ 7.34 (d, *J* = 4.4 Hz, 4H), 7.30-7.28 (m, 1H), 6.21 (s, 1H), 4.51 (s, 2H), 4.04 (s, 1H), 3.50-3.46 (m, 2H), 3.21 (s, 2H), 3.14 (s, 2H), 2.61-2.54 (m, 1H), 2.13-2.06 (m, 1H), 1.84 (d, *J* = 6.2 Hz, 4H), 1.68-1.61 (m, 2H), 1.11-1.02 (m, 44H). ppm. **^{13}C NMR** (101 MHz, $CDCl_3$): δ 155.74, 138.75, 136.51, 128.31, 127.59, 127.44, 118.19, 109.73, 86.65, 72.81, 72.19, 70.33, 47.70, 31.62, 29.92, 25.49, 25.37, 18.84, 17.73, 11.89, 11.72. ppm. **HRMS** (**APCI-TOF**) calculated for C39H67NO2Si2H(+): 638.4789; Found: 638.4784.

(13)1-((3E,5Z)-1-(triisopropylsilyl)-5-(((triisopropylsilyl)oxy)methylene)undec-3-en-1-yn-4-yl) pyrrolidine **3am** (YH00088-13)

The title compound was prepared according to the general procedure for the synthesis of **3** (91%). **^{1}H NMR** (300 MHz, $CDCl_3$): δ 6.20 (s, 1H), 4.03 (s, 1H), 3.21 (s, 2H), 3.14 (s, 2H), 2.51 (d, *J* = 4.6 Hz, 1H), 2.11-2.02 (m, 1H), 1.83 (s, 4H), 1.27 (s, 8H), 1.05 (d, *J* = 3.2 Hz, 42H), 0.87 (d, *J* = 6.6 Hz, 3H). ppm. **^{13}C NMR** (75 MHz, $CDCl_3$): δ 156.02, 136.21, 118.53, 109.76, 86.54,

71.94, 47.68, 31.83, 29.54, 28.74, 25.52, 22.76, 18.84, 18.76, 17.74, 14.13, 11.88, 11.71. ppm. **HRMS** (**APCI-TOF**) calculated for C34H65NOSi2H(+): 560.4683; Found: 560.4677.

(14)4-methyl-N-(3-methylbut-2-en-1-yl)-N-((3Z,4E)-4-(pyrrolidin-1-yl)-7-(triisopropylsilyl)-3-(((triisopropylsilyl)oxy)methylene)hept-4-en-6-yn-1-yl)benzenesulfonamide **3an** (YH00088-14)

The title compound was prepared according to the general procedure for the synthesis of **3** (85%). **^{1}H NMR** (500 MHz, $CDCl_3$): δ 7.68 (d, *J* = 8.2 Hz, 2H), 7.27 (d, *J* = 7.9 Hz, 2H), 6.26 (s, 1H), 5.03 (t, *J* = 6.5 Hz, 1H), 4.04 (s, 1H), 3.84-3.79 (m, 1H), 3.77-3.70 (m, 1H), 3.41-3.31 (m, 1H), 3.16 (s, 2H), 3.12 (s, 2H), 3.01 (t, *J* = 10.6 Hz, 1H), 2.76 (t, *J* = 10.4 Hz, 1H), 2.42 (s, 3H), 2.18 (d, *J* = 9.3 Hz, 1H), 1.84 (s, 4H), 1.66 (s, 3H), 1.62 (s, 3H), 1.12-1.09 (m, 3H), 1.05 (t, *J* = 6.1 Hz, 18H), 0.99 (t, *J* = 11.0 Hz, 18H), 0.94 (d, *J* = 7.6 Hz, 3H). ppm. **^{13}C NMR** (126 MHz, $CDCl_3$): δ 154.02, 142.68, 138.66, 137.83, 136.53, 129.45, 127.22, 119.31, 114.55, 109.50, 86.91, 73.84, 47.77, 46.71, 45.17, 31.10, 25.68, 25.45, 21.42, 18.80, 17.79, 17.69, 11.94, 11.70. ppm. **HRMS** (**APCI-TOF**) calculated for C42H72N2O3SSi2H(+): 741.4880; Found: 741.4871.

(15)1-((1Z,3E)-2-benzyl-6-(triisopropylsilyl)-1-((triisopropylsilyl)oxy)hexa-1,3-dien-5-yn-3-yl)piperidine **3ba** (YH00088-15)

The title compound was prepared according to the general procedure for the synthesis of **3** (85%). **^{1}H NMR** (400 MHz, $CDCl_3$): δ 7.26-7.21 (m, 4H), 7.18-7.14 (m, 1H), 6.30 (s, 1H), 4.42 (s, 1H), 3.69 (bs, 1H), 3.41 (bs, 1H), 2.82 (s, 4H), 1.13-1.02 (m, 48H). ppm. **^{13}C NMR** (101 MHz, $CDCl_3$): δ 157.35, 140.38, 138.23, 129.38, 127.99, 125.80, 116.99, 109.50, 88.13, 75.92, 47.39, 38.30, 25.36, 24.37, 18.86, 17.75, 11.91, 11.72. ppm. **HRMS** (**APCI-TOF**) calculated for C36H61NOSi2H(+): 580.4370; Found: 580.4364.

(16)4-((1Z,3E)-2-benzyl-6-(triisopropylsilyl)-1-((triisopropylsilyl)oxy)hexa-1,3-dien-5-yn-3-yl) morpholine **3ca** (YH00088-16)

The title compound was prepared according to the general procedure for the synthesis of **3** (89%). **^{1}H NMR** (400 MHz, $CDCl_3$): δ 7.25-7.17 (m, 5H), 6.38 (s, 1H), 4.47 (s, 1H), 3.62 (bs, 1H), 3.43 (bs, 1H), 3.26 (t, *J* = 23.1 Hz, 4H), 2.76 (s, 4H), 1.10-1.05 (m, 42H). ppm. **^{13}C NMR** (101 MHz, $CDCl_3$): δ 156.97, 140.23, 138.39, 129.25, 128.09, 126.04, 116.12, 108.12, 89.19, 77.95, 66.33, 46.64, 38.21, 18.81, 17.75, 11.89, 11.64. ppm. **HRMS** (**APCI-TOF**) calculated for C35H59NO2Si2H(+): 582.4163; Found: 582.4159.

(17)(1Z,3E)-2-benzyl-N,N-dimethyl-6-(triisopropylsilyl)-1-((triisopropylsilyl)oxy)hexa-1,3-dien-5-yn-3-amine **3da** (YH00088-17)

The title compound was prepared according to the general procedure for the synthesis of **3** (84%). **^{1}H NMR** (400 MHz, $CDCl_3$): δ 7.26-7.20 (m, 4H), 7.18-7.15 (m, 1H), 6.29 (s, 1H), 4.20 (s, 1H), 3.82 (bs, 1H), 3.38 (bs, 1H), 2.48 (s, 6H), 1.12-1.02 (m, 42H). ppm. **^{13}C NMR** (101 MHz, $CDCl_3$): δ 157.52, 140.34, 137.70, 129.19, 128.01, 125.84, 117.29, 109.67, 87.26, 73.70, 39.09, 38.06, 18.87, 17.67, 11.86, 11.73. ppm. **HRMS** (**APCI-TOF**) calculated for C33H57NOSi2H(+): 540.4057; Found: 540.4053.

(18)(1Z,3E)-2-benzyl-N,N-diethyl-6-(triisopropylsilyl)-1-((triisopropylsilyl)oxy)hexa-1,3-dien-5-yn-3-amine **3ea** (YH00088-18)

The title compound was prepared according to the general procedure for the synthesis of **3** (84%). **^{1}H NMR** (400 MHz, $CDCl_3$): δ 7.23 (t, 4.0 Hz, 4H), 7.18-7.15 (m, 1H), 6.25 (s, 1H), 4.27 (s, 1H), 3.82 (bs, 1H), 3.39 (bs, 1H), 2.96-2.91 (m, 4H), 1.12-1.04 (m, 42H), 0.77 (t, *J* = 7.0 Hz, 6H). ppm. **^{13}C NMR** (101 MHz, $CDCl_3$): δ 155.77, 140.26, 137.78, 129.43, 128.09, 125.87, 117.41, 110.32, 87.04, 72.47, 42.73, 38.33, 18.89, 17.72, 12.55, 11.90, 11.78. ppm. **HRMS** (**APCI-TOF**) calculated for C35H61NOSi2H(+): 568.4370; Found: 568.4364.

(19)(1Z,3E)-2-benzyl-N,N-dibutyl-6-(triisopropylsilyl)-1-((triisopropylsilyl)oxy)hexa-1,3-dien-5-yn-3-amine **3fa** (YH00088-19)

The title compound was prepared according to the general procedure for the synthesis of **3** (85%). **^{1}H NMR** (400 MHz, $CDCl_3$): δ 7.26-7.21 (m, 4H), 7.19-7.15 (m, 1H), 6.22 (s, 1H), 4.22 (s, 1H), 3.81 (d, *J* = 12.8 Hz, 1H), 3.39 (d, *J* = 12.6 Hz, 1H), 2.83 (s, 4H), 1.10-1.04 (m, 50H), 0.82 (t, *J* = 7.2 Hz, 6H). ppm. **^{13}C NMR** (101 MHz, $CDCl_3$): δ 156.25, 140.32, 137.79, 129.39, 128.10, 125.87, 117.43, 110.44, 87.02, 72.27, 49.45, 38.41, 29.35, 20.37, 18.90, 17.72, 13.82, 11.90, 11.79. ppm. **HRMS** (**APCI-TOF**) calculated for C39H69NOSi2H(+): 624.4996; Found: 624.4990.

(20)(1Z,3E)-2-benzyl-N-ethyl-N-methyl-6-(triisopropylsilyl)-1-((triisopropylsilyl)oxy)hexa-1,3-dien-5-yn-3-amine **3ga** (YH00088-20)

The title compound was prepared according to the general procedure for the synthesis of **3** (99%). **^{1}H NMR** (500 MHz, $CDCl_3$): δ 7.26-7.23 (m, 4H), 7.18-7.14 (m, 1H), 6.29 (s, 1H), 4.23 (s, 1H), 3.85 (bs, 1H), 3.37 (bs, 1H), 2.93 (s, 2H), 2.47 (s, 3H), 1.12-1.04 (m, 42H), 0.68 (t, *J* = 7.1 Hz, 3H). ppm. **^{13}C NMR** (126 MHz, $CDCl_3$): δ 156.57, 140.33, 137.86, 129.34, 128.03, 125.84, 117.40, 110.02, 87.23, 73.26, 45.65, 38.17, 35.86, 18.86, 17.67, 11.93, 11.82, 11.80. ppm. **HRMS** (**APCI-TOF**) calculated for C34H59NOSi2H(+): 554.4213; Found: 554.4206.

(21)N-((1Z,3E)-2-benzyl-6-(triisopropylsilyl)-1-((triisopropylsilyl)oxy)hexa-1,3-dien-5-yn-3-yl)-N-methylcyclohexanamine **3ha** (YH00088-21)

The title compound was prepared according to the general procedure for the synthesis of **3** (79%). **^{1}H NMR** (500 MHz, $CDCl_3$): δ 7.26-7.22 (m, 4H), 7.17-7.14 (m, 1H), 6.31 (s, 1H), 4.23 (s, 1H), 3.86 (d, *J* = 13.8 Hz, 1H), 3.37 (d, *J* = 13.4 Hz, 1H), 3.21-3.17 (m, 1H), 2.41 (s, 3H), 1.68 (s, 2H), 1.49 (s, 2H), 1.14-1.05 (m, 46H), 0.91-0.86 (m, 3H). ppm. **^{13}C NMR** (126 MHz, $CDCl_3$): δ 156.78, 140.31, 138.13, 129.39, 128.13, 125.82, 117.45, 110.33, 86.94, 72.93, 57.88, 38.44, 30.66, 30.36, 26.04, 25.72, 18.87, 17.73, 12.01, 11.82. ppm. **HRMS** (**APCI-TOF**) calculated for C38H65NOSi2H(+): 608.4683; Found: 608.4678.

(22)2-((1Z,3E)-2-benzyl-6-(triisopropylsilyl)-1-((triisopropylsilyl)oxy)hexa-1,3-dien-5-yn-3-yl)-1,2,3,4-tetrahydroisoquinoline **3ia** (YH00088-22)

The title compound was prepared according to the general procedure for the synthesis of **3** (96%). **^{1}H NMR** (500 MHz, $CDCl_3$): δ 7.23-7.18 (m, 4H), 7.12-7.19 (m, 3H), 7.09 (t, 5.0 Hz, 1H), 6.92 (d, *J* = 5.9 Hz, 1H), 6.43 (s, 1H), 4.47 (s, 1H), 4.07 (s, 2H), 3.77 (bs, 1H), 3.38 (bs, 1H), 3.08 (s, 2H), 2.29 (bs, 2H), 1.11 (d, *J* = 5.4 Hz, 21H), 1.03 (d, *J* = 6.6 Hz, 21H). ppm. **^{13}C NMR**

(126 MHz, $CDCl_3$): δ 156.31, 140.26, 138.37, 135.35, 134.04, 129.13, 128.11, 127.99, 126.19, 125.92, 125.74, 117.13, 109.11, 88.08, 75.65, 48.37, 44.15, 38.31, 29.19, 18.84, 17.68, 11.94, 11.76. ppm. **HRMS** (**APCI-TOF**) calculated for C40H61NOSi2H(+): 628.4370; Found: 628.4356.

(23)1-((1Z,3E)-2-benzyl-6-(triisopropylsilyl)-1-((triisopropylsilyl)oxy)hexa-1,3-dien-5-yn-3-yl)azocane **3ja** (YH00088-23)

The title compound was prepared according to the general procedure for the synthesis of **3** (95%). ^{1}**H NMR** (500 MHz, $CDCl_3$): δ 7.26-7.20 (m, 4H), 7.18-7.15 (m, 1H), 6.25 (s, 1H), 4.25 (s, 1H), 3.87 (d, *J* = 14.5 Hz, 1H), 3.40 (d, *J* = 14.7 Hz, 1H), 3.07 (s, 4H), 1.27 (s, 4H), 1.13-1.04 (m, 48H). ppm. ^{13}**C NMR** (126 MHz, $CDCl_3$): δ 157.25, 140.23, 138.03, 129.58, 128.15, 125.94, 117.66, 110.42, 87.00, 72.53, 50.58, 38.32, 27.47, 26.83, 24.67, 18.88, 17.73, 11.97, 11.83. ppm. **HRMS** (**APCI-TOF**) calculated for C38H65NOSi2H(+): 608.4683; Found: 608.4680.

(24)1-((1Z,3E)-2-benzyl-6-(triisopropylsilyl)-1-((triisopropylsilyl)oxy)hexa-1,3-dien-5-yn-3-yl)-2,5-dihydro-1H-pyrrole **3ka** (YH00088-24)

The title compound was prepared according to the general procedure for the synthesis of **3** (84%). ^{1}**H NMR** (400 MHz, $CDCl_3$): δ 7.24-7.21 (m, 4H), 7.15-7.13 (m, 1H), 6.28 (s, 1H), 5.61 (s, 2H), 3.93 (dd, *J* = 14.2, 1.4 Hz, 1H), 3.86 (d, *J* = 11.3 Hz, 2H), 3.45 (bs, 2H), 3.35 (d, *J* = 14.2 Hz, 1H), 1.12-1.01 (m, 42H). ppm. ^{13}**C NMR** (101 MHz, $CDCl_3$): δ 154.45, 140.35, 137.37, 129.13, 128.06, 125.91, 125.69, 118.27, 109.43, 87.52, 73.03, 54.36, 37.68, 18.86, 17.63, 11.85, 11.74. ppm. **HRMS** (**APCI-TOF**) calculated for C35H57NOSi2H(+): 564.4057; Found: 564.4048.

(25)1-((1Z,3E)-2-benzyl-6-(triisopropylsilyl)-1-((triisopropylsilyl)oxy)hexa-1,3-dien-5-yn-3-yl)piperidin-4-ol **3la** (YH00088-025)

The title compound was prepared according to the general procedure for the synthesis of **3** (90%). ^{1}**H NMR** (400 MHz, $CDCl_3$): δ 7.26-7.21 (m, 4H), 7.18-7.15 (m, 1H), 6.35 (s, 1H), 4.47 (s, 1H), 3.72 (bs, 1H), 3.60-3.55 (m, 1H), 3.48 (bs, 1H), 3.27-3.22 (m, 2H), 2.51 (s, 2H), 1.53 (d, 8.0 Hz, 2H), 1.11-1.04 (m, 44H). ppm. ^{13}**C NMR** (101 MHz, $CDCl_3$): δ 156.59, 140.36, 138.38, 129.33, 128.07, 125.93, 116.86, 108.88, 88.70, 77.16, 68.05, 44.07, 38.23, 33.81, 18.83, 17.74, 11.91, 11.68. ppm. **HRMS** (**APCI-TOF**) calculated for C36H61NO2Si2H(+): 596.4319; Found: 596.4315.

(26)tert-butyl-4-((1Z,3E)-2-benzyl-6-(triisopropylsilyl)-1-((triisopropylsilyl)oxy)hexa-1,3-dien-5-yn-3-yl)piperazine-1-carboxylate **3ma** (YH00088-26)

The title compound was prepared according to the general procedure for the synthesis of **3** (91%). ^{1}**H NMR** (400 MHz, $CDCl_3$): δ 7.21-7.15 (m, 5H), 6.39 (s, 1H), 4.46 (s, 1H), 3.53 (bs, 2H), 3.01 (s, 4H), 2.78 (s, 4H), 1.42 (s, 9H), 1.10-1.04 (m, 42H). ppm. ^{13}**C NMR** (101 MHz, $CDCl_3$): δ 156.57, 154.62, 140.18, 138.48, 129.21, 128.12, 126.08, 116.45, 108.18, 89.18, 79.68, 78.02, 46.02, 46.01, 38.18, 28.38, 18.81, 17.74, 11.89, 11.65. ppm. **HRMS** (**APCI-TOF**) calculated for C40H68N2O3Si2H(+): 681.4847; Found: 681.4850.

(27)1-((1Z,3E)-2-benzyl-6-(triisopropylsilyl)-1-((triisopropylsilyl)oxy)hexa-1,3-dien-5-yn-3-yl)-4-methylpiperazine **3na** (YH00088-27)

The title compound was prepared according to the general procedure for the synthesis of **3** (70%). [1]**H NMR** (400 MHz, $CDCl_3$): δ 7.25-7.14 (m, 5H), 6.35 (s, 1H), 4.46 (s, 1H), 3.72 (bs, 1H), 3.43 (bs, 1H), 2.84 (s, 4H), 2.14 (s, 3H), 2.03 (s, 4H), 1.10-1.04 (m, 42H). ppm. [13]**C NMR** (101 MHz, $CDCl_3$) δ 156.84, 140.28, 138.37, 129.30, 128.07, 125.94, 116.48, 108.58, 88.74, 77.58, 54.51, 46.16, 46.04, 38.24, 18.82, 17.78, 11.90, 11.67. ppm. **HRMS** (**APCI-TOF**) calculated for C36H62N2OSi2H(+): 595.4479; Found: 595.4472.

(28)1-((1Z,3E)-2-benzyl-6-(triisopropylsilyl)-1-((triisopropylsilyl)oxy)hexa-1,3-dien-5-yn-3-yl)piperidin-4-yl 3,5-dinitrobenzoate **3oa** (YH00088-28)

The title compound was prepared according to the general procedure for the synthesis of **3** (70%). [1]**H NMR** (500 MHz, $CDCl_3$): δ 9.23 (t, *J* = 2.1 Hz, 1H), 9.08 (d, *J* = 2.1 Hz, 2H), 7.29-7.24 (m, 4H), 7.21-7.18 (m, 1H), 6.42 (s, 1H), 5.05-5.02 (m, 1H), 4.54 (s, 1H), 3.80 (bs, 1H), 3.48 (bs, 1H), 3.25 (d, *J* = 9.2 Hz, 2H), 2.76 (s, 2H), 1.64 (s, 2H), 1.15-1.04 (m, 44H). ppm. [13]**C NMR** (126 MHz, $CDCl_3$): δ 161.71, 156.06, 148.75, 140.44, 138.63, 134.30, 129.39, 129.27, 128.19, 126.04, 122.30, 116.74, 108.32, 89.38, 78.18, 73.50, 43.82, 38.20, 30.01, 18.84, 17.75, 11.97, 11.71. ppm. **HRMS** (**APCI-TOF**) calculated for C43H63N3O7Si2H(+): 790.4283; Found: 790.4279.

(29)1-((1Z,3E)-2-((1,3-dioxoisoindolin-2-yl)methyl)-6-(triisopropylsilyl)-1-((triisopropylsilyl)oxy)hexa-1,3-dien-5-yn-3-yl)piperidin-4-yl 3,5-dinitrobenzoate **3in** (YH00088-29)

The title compound was prepared according to the general procedure for the synthesis of **3.** [1]**H NMR** (400 MHz, $CDCl_3$): δ 9.22 (t, *J* = 2.1 Hz, 1H), 9.01 (d, *J* = 2.1 Hz, 2H), 7.88-7.86 (m, 2H), 7.77-7.75 (m, 2H), 6.80 (s, 1H), 5.03-4.49 (m, 1H), 4.79 (s, 1H), 4.66 (s, 1H), 4.20 (s, 1H), 3.31 (s, 2H), 2.79-2.72 (m, 2H), 1.75 (d, *J* = 9.1 Hz, 2H), 1.21-1.16 (m, 3H), 1.09-1.05 (m, 38H), 0.87-0.83 (m, 2H). ppm. [13]**C NMR** (101 MHz, $CDCl_3$): δ 167.99, 161.56, 154.58, 148.64, 143.69, 134.07, 133.90, 132.37, 129.24, 123.16, 122.36, 110.78, 106.97, 89.66, 83.21, 73.08, 44.46, 39.85, 30.29, 18.76, 17.70, 11.87, 11.56. ppm. **HRMS** (**ESI-TOF**) calculated for C45H62N4O9Si2H(+): 859.4134; Found: 859.4132.

(30)(E)-1-benzyl-3-bromo-4-(bromo(triisopropylsilyl)methylene)-2-(pyrrolidin-1-yl)cyclobut-2-enecarbaldehyde **4a** (YH00101-1)

The title compound was prepared according to the general procedure for the synthesis of **4** (48%). [1]**H NMR** (300 MHz, $CDCl_3$): δ 9.59 (s, 1H), 7.22-7.14 (m, 5H), 3.74 (d, *J* = 14.8 Hz, 1H), 3.63 (s, 2H), 3.36 (s, 2H), 3.17 (d, *J* = 14.8 Hz, 1H), 1.97-1.87 (m, 4H), 1.60-1.51 (m, 3H), 1.12 (d, *J* = 7.5 Hz, 9H), 0.99 (d, *J* = 7.5 Hz, 9H). ppm. [13]**C NMR** (75 MHz, $CDCl_3$): δ 199.74, 148.45, 147.10, 135.72, 129.69, 127.97, 126.54, 103.43, 76.73, 74.07, 48.33, 32.15, 25.16, 18.85, 13.39. ppm. **HRMS** (**ESI-TOF**) calculated for C26H37Br2NOSiH(+): 566.1089; Found: 566.1085.

(31)(E)-1-benzyl-3-bromo-4-(bromo(triisopropylsilyl)methylene)-2-(piperidin-1-yl)cyclobut-2-enecarbaldehyde **4b** (YH00101-2)

The title compound was prepared according to the general procedure for the synthesis of **4** (52%). [1]**H NMR** (400 MHz, $CDCl_3$): δ 9.57 (s, 1H), 7.25-7.16 (m, 5H), 3.73 (d, *J* = 14.7 Hz, 1H), 3.46 (s, 2H), 3.28 (d, *J* = 12.7 Hz, 2H), 3.09 (d, *J* = 14.7 Hz, 1H), 1.65 (d, *J* = 3.0 Hz, 4H),

1.56-1.50 (m, 3H), 1.10 (t, *J* = 6.8 Hz, 11H), 0.98 (d, *J* = 7.5 Hz, 9H). ppm. **^{13}C NMR** (101 MHz, $CDCl_3$): δ 199.86, 149.22, 146.46, 135.59, 129.88, 127.88, 126.53, 104.10, 76.77, 74.16, 47.79, 32.33, 25.61, 23.78, 18.87, 13.50. ppm. **HRMS** (**ESI-TOF**) calculated for C27H39Br2NOSiH(+): 580.1246; Found: 580.1244.

(32)(E)-1-benzyl-3-bromo-4-(bromo(triisopropylsilyl)methylene)-2-(diethylamino)cyclobut-2-enecarbaldehyde **4c** (YH00101-3)

The title compound was prepared according to the general procedure for the synthesis of **4** (63%). **^{1}H NMR** (400 MHz, $CDCl_3$): δ 9.56 (s, 1H), 7.25-7.13 (m, 5H), 3.79 (d, *J* = 14.9 Hz, 1H), 3.50 (bs, 2H), 3.17 (d, *J* = 14.9 Hz, 1H), 3.13-3.06 (m, 2H), 1.57 (t, *J* = 7.5 Hz, 3H), 1.13 (d, *J* = 7.5 Hz, 9H), 1.09-0.95 (m, 15H). ppm. **^{13}C NMR** (101 MHz, $CDCl_3$): δ 199.58, 149.35, 146.68, 135.56, 129.91, 127.85, 126.58, 103.14, 76.11, 73.81, 32.20, 18.94, 13.75, 13.62. ppm. **HRMS** (**ESI-TOF**) calculated for C26H39Br2NOSiH(+): 568.1246; Found: 568.1247.

(33)(E)-1-benzyl-3-bromo-4-(bromo(triisopropylsilyl)methylene)-2-(ethyl(methyl)amino)cyclobut-2-enecarbaldehyde **4d** (YH00101-4)

The title compound was prepared according to the general procedure for the synthesis of **4** (65%). **^{1}H NMR** (400 MHz, $CDCl_3$): δ 9.57 (s, 1H), 7.25-7.16 (m, 5H), 3.77 (d, *J* = 14.8 Hz, 1H), 3.57-3.44 (m, 1H), 3.18 (dd, *J* = 14.4, 8.5 Hz, 2H), 2.94 (s, 3H), 1.58-1.50 (m, 3H), 1.13 (d, *J* = 7.5 Hz, 9H), 1.08 (d, *J* = 7.4 Hz, 3H), 1.02 (d, *J* = 7.5 Hz, 9H). ppm. **^{13}C NMR** (101 MHz, $CDCl_3$): δ 199.73, 149.81, 146.55, 135.58, 129.81, 127.90, 126.58, 103.56, 77.22, 73.91, 46.01, 36.56, 32.23, 18.91, 13.55, 12.96. ppm. **HRMS** (**ESI-TOF**) calculated for C25H37Br2NOSiH(+): 554.1089; Found: 554.1086.

(34)(E)-1-benzyl-3-bromo-4-(bromo(triisopropylsilyl)methylene)-2-(3,4-dihydroisoquinolin-2(1H)-yl)cyclobut-2-enecarbaldehyde **4e** (YH00101-5)

The title compound was prepared according to the general procedure for the synthesis of **4** (48% yield). **^{1}H NMR** (400 MHz, $CDCl_3$): δ 9.60 (s, 1H), 7.24-7.21 (m, 2H), 7.17-7.09 (m, 7H), 4.76 (d, *J* = 14.6 Hz, 1H), 4.45 (d, *J* = 16.2 Hz, 1H), 3.79 (d, *J* = 14.8 Hz, 1H), 3.71 (s, 1H), 3.65 (d, *J* = 5.0 Hz, 1H), 3.20 (d, *J* = 14.8 Hz, 1H), 2.96-2.89 (m, 1H), 2.86-2.80 (m, 1H), 1.57-1.52 (m, 3H), 1.12 (d, *J* = 7.5 Hz, 9H), 1.01 (d, *J* = 7.5 Hz, 9H). ppm. **^{13}C NMR** (101 MHz, $CDCl_3$): δ 199.77, 149.05, 146.40, 135.36, 133.45, 131.87, 129.81, 128.99, 127.92, 127.09, 126.69, 126.57, 126.19, 104.89, 77.22, 74.02, 48.44, 44.44, 32.48, 28.57, 18.93, 13.51. ppm. **HRMS** (**ESI-TOF**) calculated for C31H39Br2NOSiH(+): 628.1246; Found: 628.1238.

(35)(E)-2-(azocan-1-yl)-1-benzyl-3-bromo-4-(bromo(triisopropylsilyl)methylene)cyclobut-2-enecarbaldehyde **4f** (YH00101-6)

The title compound was prepared according to the general procedure for the synthesis of **4** (46%). **^{1}H NMR** (400 MHz, $CDCl_3$): δ 9.58 (s, 1H), 7.24-7.14 (m, 5H), 3.80 (d, *J* = 14.9 Hz, 1H), 3.50 (bs, 2H), 3.21 (d, *J* = 14.9 Hz, 3H), 1.82 (bs, 2H), 1.62 (s, 2H), 1.58-1.51 (m, 9H), 1.13 (d, *J* = 7.5 Hz, 9H), 1.01 (d, *J* = 7.5 Hz, 9H). ppm. **^{13}C NMR** (101 MHz, $CDCl_3$): δ 199.58, 150.20, 146.64, 135.76, 129.78, 127.91, 126.52, 103.49, 77.23, 74.34, 49.94, 32.38, 27.16, 26.67, 24.85, 18.98, 13.64. ppm. **HRMS** (**ESI-TOF**) calculated for C29H43Br2NOSiH(+): 608.1559; Found: 608.1555.

(36)(E)-2-benzyl-3-(pyrrolidin-1-yl)-5-((triisopropylsilyl)methylene)cyclopent-2-enone **5a** (YH00101-7)

The title compound was prepared according to the general procedure for the synthesis of **5** (56%). **^{1}H NMR** (500 MHz, $CDCl_3$): δ 7.25 (t, *J* = 7.5 Hz, 2H), 7.19 (d, *J* = 7.2 Hz, 2H), 7.14 (t, *J* = 7.2 Hz, 1H), 6.65 (d, *J* = 1.6 Hz, 1H), 3.89 (s, 2H), 3.60 (s, 4H), 3.30 (s, 2H), 1.89 (t, *J* = 2.9 Hz, 4H), 1.24-1.20 (m, 3H), 1.11 (d, *J* = 7.2 Hz, 18H). ppm. **^{13}C NMR** (126 MHz, $CDCl_3$): δ 190.50, 166.86, 149.01, 142.73, 128.38, 127.98, 125.54, 122.11, 113.22, 49.53, 35.76, 29.41, 25.19, 18.89, 11.92. ppm. **HRMS** (**ESI-TOF**) calculated for C26H39NOSiH(+): 410.2879; Found: 410.2856.

(37)(E)-2-benzyl-3-morpholino-5-((triisopropylsilyl)methylene)cyclopent-2-enone**5b** (YH00101-8)

The title compound was prepared according to the general procedure for the synthesis of **5** (60%). **^{1}H NMR** (400 MHz, $CDCl_3$): δ 7.27-7.24 (m, 2H), 7.17 (d, *J* = 7.3 Hz, 1H), 7.11 (d, *J* = 7.1 Hz, 2H), 6.70 (t, *J* = 1.7 Hz, 1H), 3.90 (s, 2H), 3.54-3.50 (m, 8H), 3.26 (s, 2H), 1.25-1.19 (m, 3H), 1.11 (d, *J* = 6.9 Hz, 18H). ppm. **^{13}C NMR** (101 MHz, $CDCl_3$): δ 191.39, 166.91, 147.87, 140.51, 128.51, 127.92, 125.91, 123.35, 113.31, 66.61, 48.74, 34.65, 30.23, 18.88, 11.88. ppm. **HRMS** (**ESI-TOF**) calculated for C26H39NO2SiH(+): 426.2828; Found: 426.2821.

(38)(E)-2-benzyl-3-(diethylamino)-5-((triisopropylsilyl)methylene)cyclopent-2-enone **5c** (YH00101-9)

The title compound was prepared according to the general procedure for the synthesis of **5** (52%). **^{1}H NMR** (400 MHz, $CDCl_3$): δ 7.27-7.22 (m, 2H), 7.14 (dd, *J* = 6.6, 4.6 Hz, 3H), 6.64 (s, 1H), 3.86 (s, 2H), 3.36 (q, *J* = 7.1 Hz, 4H), 3.32 (d, *J* = 1.5 Hz, 2H), 1.25-1.21 (m, 3H), 1.18 (t, *J* = 7.1 Hz, 6H), 1.11 (d, *J* = 6.9 Hz, 18H). ppm. **^{13}C NMR** (101 MHz, $CDCl_3$): δ 190.92, 167.52, 148.64, 141.88, 128.40, 127.82, 125.65, 121.77, 112.35, 45.07, 34.74, 30.14, 18.88, 14.67, 11.88. ppm. **HRMS** (**ESI-TOF**) calculated for C26H41NOSiH(+): 412.3036; Found: 412.3031.

(39)(E)-2-benzyl-3-(piperidin-1-yl)-5-((triisopropylsilyl)methylene)cyclopent-2-enone **5d** (YH00101-10)

The title compound was prepared according to the general procedure for the synthesis of **5** (54%). **^{1}H NMR** (400 MHz, $CDCl_3$): δ 7.23 (d, *J* = 7.4 Hz, 2H), 7.14 (dd, *J* = 7.3, 3.5 Hz, 3H), 6.65 (t, *J* = 1.7 Hz, 1H), 3.90 (s, 2H), 3.49-3.42 (m, 4H), 3.25 (d, *J* = 1.3 Hz, 2H), 1.64-1.59 (m, 2H), 1.50-1.44 (m, 4H), 1.25-1.18 (m, 3H), 1.11 (d, *J* = 6.9 Hz, 18H). ppm. **^{13}C NMR** (101 MHz, $CDCl_3$): δ 191.21, 167.05, 148.57, 141.10, 128.34, 128.02, 125.64, 121.88, 112.47, 50.05, 34.98, 30.57, 26.41, 24.25, 18.89, 11.90. ppm. **HRMS** (**ESI-TOF**) calculated for C27H41NOSiH(+): 424.3036; Found: 424.3030.

(40)(E)-2-hexyl-3-(pyrrolidin-1-yl)-5-((triisopropylsilyl)methylene)cyclopent-2-enone **5e** (YH00101-11)

The title compound was prepared according to the general procedure for the synthesis of **5** (45%). **^{1}H NMR** (400 MHz, $CDCl_3$): δ 6.55 (d, *J* = 1.7 Hz, 1H), 3.65 (s, 4H), 3.21 (d, *J* = 1.4 Hz, 2H), 2.46-2.39 (m, 2H), 2.00 (s, 4H), 1.41-1.27 (m, 8H), 1.22-1.15 (m, 3H), 1.08 (d, *J* = 6.9 Hz, 18H), 0.87 (t, *J* = 6.9 Hz, 3H). ppm. **^{13}C NMR** (101 MHz, $CDCl_3$): δ 190.42, 165.81, 149.33,

121.18, 115.73, 49.45, 35.57, 31.83, 29.55, 24.17, 22.67, 18.86, 14.15, 11.87. ppm. **HRMS** (**ESI-TOF**) calculated for C25H45NOSiH(+): 404.3349; Found: 404.3342.

(41)(E)-2-(4-((tert-butyldimethylsilyl)oxy)butyl)-3-(pyrrolidin-1-yl)-5-((triisopropylsilyl)methylene)cyclopent-2-enone **5f** (YH00101-12)

The title compound was prepared according to the general procedure for the synthesis of **5** (41%). **^{1}H NMR** (400 MHz, $CDCl_3$): δ 6.54 (s, 1H), 3.63 (t, *J* = 6.3 Hz, 6H), 3.21 (d, *J* = 1.4 Hz, 2H), 2.49-2.42 (m, 2H), 1.99 (s, 4H), 1.61-1.55 (m, 2H), 1.51- 1.44 (m, 2H), 1.22-1.15 (m, 3H), 1.08 (d, *J* = 6.9 Hz, 18H), 0.88 (s, 9H), 0.04 (s, 6H). ppm. **^{13}C NMR** (101 MHz, $CDCl_3$): δ 190.39, 165.87, 149.32, 121.19, 115.35, 63.06, 49.45, 35.59, 32.86, 27.79, 25.99, 23.66, 19.31, 18.86, 18.34, 11.88, -5.22. ppm. **HRMS** (**ESI-TOF**) calculated for C29H55NO2Si2H(+): 506.3850; Found: 506.3849.

(42)(E)-2-(4-(benzyloxy)butyl)-3-(pyrrolidin-1-yl)-5-((triisopropylsilyl)methylene)cyclopent-2-enone **5g** (YH00101-13)

The title compound was prepared according to the general procedure for the synthesis of **5** (63%). **^{1}H NMR** (400 MHz, $CDCl_3$): δ 7.33 (d, *J* = 4.4 Hz, 4H), 7.28 (d, *J* = 4.5 Hz, 1H), 6.55 (s, 1H), 4.49 (s, 2H), 3.64 (s, 4H), 3.52 (t, *J* = 6.5 Hz, 2H), 3.21 (d, *J* = 1.4 Hz, 2H), 2.51-2.44 (m, 2H), 1.96 (s, 4H), 1.71-1.66 (m, 2H), 1.54-1.51 (m, 2H), 1.22-1.16 (m, 3H), 1.08 (d, *J* = 6.9 Hz, 18H). ppm. **^{13}C NMR** (101 MHz, $CDCl_3$): δ 190.47, 166.03, 149.21, 138.71, 128.32, 127.62, 127.43, 121.39, 115.23, 72.86, 70.43, 49.51, 35.61, 29.66, 29.33, 28.09, 23.71, 18.86, 11.87. ppm. **HRMS** (**ESI-TOF**) calculated for C30H47NO2SiH(+): 482.3454; Found: 482.3455.

(43)5-benzyl-4-(pyrrolidin-1-yl)-3-((triisopropylsilyl)ethynyl)-2H-pyran-2-one **6a** (YH00101-14)

The title compound was prepared according to the general procedure for the synthesis of **6** (60%). **^{1}H NMR** (400 MHz, $CDCl_3$): δ 7.32 (t, *J* = 7.3 Hz, 2H), 7.26-7.21 (m, 1H), 7.09 (d, *J* = 7.1 Hz, 2H), 7.00 (s, 1H), 3.82 (s, 2H), 3.79 (t, *J* = 6.6 Hz, 4H), 1.71-1.68 (m, 4H), 1.12 (s, 21H). ppm. **^{13}C NMR** (75 MHz, $CDCl_3$): δ 164.39, 158.87, 149.19, 138.60, 128.84, 127.55, 126.83, 112.26, 103.15, 99.63, 88.17, 52.49, 36.43, 25.75, 18.76, 11.58. ppm. **HRMS** (**ESI-TOF**) calculated for C27H37NO2SiH(+): 436.2672; Found: 436.2669.

(44)5-hexyl-4-(pyrrolidin-1-yl)-3-((triisopropylsilyl)ethynyl)-2H-pyran-2-one **6b** (YH00101-15)

The title compound was prepared according to the general procedure for the synthesis of **6** (38%). **^{1}H NMR** (400 MHz, $CDCl_3$): δ 7.00 (s, 1H), 3.91 (t, *J* = 6.5 Hz, 4H), 2.43-2.36 (m, 2H), 1.92 (t, *J* = 6.5 Hz, 4H), 1.28 (s, 8H), 1.12 (s, 18H), 1.06 (d, *J* = 3.8 Hz, 3H), 0.88 (d, *J* = 6.6 Hz, 3H). ppm. **^{13}C NMR** (101 MHz, $CDCl_3$): δ 164.50, 158.93, 146.91, 114.64, 103.39, 99.17, 88.08, 52.59, 31.52, 30.89, 29.68, 28.75, 25.91, 22.55, 18.75, 14.00, 11.60. ppm. **HRMS** (**ESI-TOF**) calculated for C26H43NO2SiH(+): 430.3141; Found: 430.3140.

(45)5-(4-(benzyloxy)butyl)-4-(pyrrolidin-1-yl)-3-((triisopropylsilyl)ethynyl)-2H-pyran-2-one **6c** (YH00101-16)

The title compound was prepared according to the general procedure for the synthesis of **6** (53% yield). **^{1}H NMR** (400 MHz, $CDCl_3$): δ 7.38-7.29 (m, 5H), 6.99 (s, 1H), 4.49 (s, 2H), 3.89 (t,

J = 6.6 Hz, 4H), 3.49 (t, *J* = 6.0 Hz, 2H), 2.46-2.39 (m, 2H), 1.89-1.83 (m, 4H), 1.62 (d, *J* = 7.1 Hz, 2H), 1.53 (dd, *J* = 9.5, 5.9 Hz, 2H), 1.12 (s, 21H). ppm. **^{13}C NMR** (101 MHz, $CDCl_3$): δ 164.46, 158.80, 147.00, 138.37, 128.40, 127.64, 127.61, 114.44, 103.36, 99.17, 92.40, 88.01, 72.98, 69.65, 52.60, 30.68, 29.12, 26.52, 25.87, 18.76, 11.60. ppm. **HRMS** (**ESI-TOF**) calculated for C31H45NO3SiH(+): 508.3247; Found: 508.3239.

(46)5-(4-((tert-butyldimethylsilyl)oxy)butyl)-4-(pyrrolidin-1-yl)-3-((triisopropylsilyl)ethynyl)-2H-pyran-2-one **6d** (YH00101-17)

The title compound was prepared according to the general procedure for the synthesis of **6** (32%). **^{1}H NMR** (400 MHz, $CDCl_3$): δ 7.01 (s, 1H), 3.91 (t, *J* = 6.6 Hz, 4H), 3.62 (t, *J* = 5.1 Hz, 2H), 2.43 (t, *J* = 6.9 Hz, 2H), 1.93-1.88 (m, 4H), 1.53-1.49 (m, 4H), 1.12 (s, 18H), 1.05 (d, *J* = 6.5 Hz, 3H), 0.89 (s, 9H), 0.04 (s, 6H). ppm. **^{13}C NMR** (101 MHz, $CDCl_3$): δ 164.47, 158.82, 146.96, 114.54, 103.38, 99.15, 88.00, 62.53, 52.59, 32.17, 30.75, 26.26, 25.93, 25.90, 18.76, 17.65, 11.60, -5.30. ppm. **HRMS** (**ESI-TOF**) calculated for C30H53NO3Si2H(+): 532.3642; Found: 532.3636.

(47)methyl 4-(2-oxo-4-(pyrrolidin-1-yl)-3-((triisopropylsilyl)ethynyl)-2H-pyran-5-yl)butanoate **6e** (YH00101-18)

The title compound was prepared according to the general procedure for the synthesis of **6** (40%). **^{1}H NMR** (400 MHz, $CDCl_3$): δ 7.02 (s, 1H), 3.92 (t, *J* = 6.6 Hz, 4H), 3.68 (s, 3H), 2.49-2.44 (m, 2H), 2.33 (t, *J* = 7.1 Hz, 2H), 1.95-1.90 (m, 4H), 1.79-1.74 (m, 2H), 1.12 (s, 21H). ppm. **^{13}C NMR** (101 MHz, $CDCl_3$): δ 173.36, 164.31, 158.55, 147.31, 113.71, 103.16, 99.39, 88.16, 52.61, 51.74, 32.87, 30.23, 25.91, 24.70, 18.75, 11.59. ppm. **HRMS** (**ESI-TOF**) calculated for C25H39NO4SiH(+): 446.2727; Found: 446.2719.

(48)4-methyl-N-(3-methylbut-2-en-1-yl)-N-(2-(2-oxo-4-(pyrrolidin-1-yl)-3-((triisopropylsilyl)ethynyl)-2H-pyran-5-yl)ethyl)benzenesulfonamide **6f** (YH00101-19)

The title compound was prepared according to the general procedure for the synthesis of **6** (37%). **^{1}H NMR** (400 MHz, $CDCl_3$): δ 7.64 (d, *J* = 8.3 Hz, 2H), 7.32 (d, *J* = 8.0 Hz, 2H), 6.94 (s, 1H), 4.97 (t, *J* = 7.1 Hz, 1H), 3.94 (t, *J* = 6.5 Hz, 4H), 3.77 (d, *J* = 7.0 Hz, 2H), 3.05 (dd, *J* = 9.2, 6.4 Hz, 2H), 2.73 (dd, *J* = 9.2, 6.3 Hz, 2H), 2.44 (s, 3H), 1.94 (t, *J* = 6.5 Hz, 4H), 1.69 (s, 3H), 1.61 (s, 3H), 1.12 (s, 21H). ppm. **^{13}C NMR** (101 MHz, $CDCl_3$): δ 164.01, 158.28, 147.95, 143.60, 137.54, 136.30, 129.78, 127.13, 118.86, 111.64, 102.96, 99.64, 88.29, 52.65, 47.67, 46.47, 31.38, 29.71, 25.97, 21.55, 18.74, 17.87, 11.58. ppm. **HRMS** (**ESI-TOF**) calculated for C34H50N2O4SSiH(+): 611.3339; Found: 611.3335.

(49)5-(2-((3-methylbut-2-en-1-yl)oxy)ethyl)-4-(pyrrolidin-1-yl)-3-((triisopropylsilyl)ethynyl)-2H-pyran-2-one **6g** (YH00101-20)

The title compound was prepared according to the general procedure for the synthesis of **6** (44%). **^{1}H NMR** (400 MHz, $CDCl_3$): δ 7.09 (s, 1H), 5.34-5.27 (m, 1H), 3.94 (d, *J* = 7.0 Hz, 2H), 3.91 (t, *J* = 6.6 Hz, 4H), 3.46 (t, *J* = 7.0 Hz, 2H), 2.71 (t, *J* = 6.8 Hz, 2H), 1.93-1.87 (m, 4H), 1.75 (s, 3H), 1.67 (s, 3H), 1.11 (s, 21H). ppm. **^{13}C NMR** (101 MHz, $CDCl_3$): δ 164.29, 158.91, 147.84, 137.77, 120.53, 111.54, 103.17, 99.34, 88.16, 68.95, 67.49, 52.82, 31.05, 25.92, 25.82, 18.75,

18.06, 11.58. ppm. **HRMS** (**ESI-TOF**) calculated for C27H43NO3SiH(+): 458.3090; Found: 458.3085.

(50)6-benzyl-3-phenyl-5-(pyrrolidin-1-yl)-4-((triisopropylsilyl)ethynyl)-2H-azepine **7a** (YH00101-21)

The title compound was prepared according to the procedure for the synthesis of **7a** (54%). **^{1}H NMR** (400 MHz, $CDCl_3$): δ 8.13 (s, 1H), 7.36 (d, *J* = 7.0 Hz, 2H), 7.28-7.22 (m, 4H), 7.19-7.15 (m, 2H), 7.06 (d, *J* = 7.0 Hz, 2H), 4.39 (s, 2H), 3.95 (s, 2H), 3.39 (t, *J* = 6.5 Hz, 4H), 1.77-1.74 (m, 4H), 1.10 (d, *J* = 4.3 Hz, 21H). ppm. **^{13}C NMR** (101 MHz, $CDCl_3$): δ 163.49, 157.04, 150.96, 140.52, 139.73, 129.01, 128.48, 128.28, 128.23, 128.20, 126.04, 125.95, 103.76, 102.92, 77.21, 50.48, 42.38, 36.56, 25.92, 18.64, 11.49. ppm. **HRMS** (**ESI-TOF**) calculated for C34H44N2SiH(+): 509.3352; Found: 509.3346.

(51)(4E,6Z)-6-benzyl-5-(pyrrolidin-1-yl)-4-((triisopropylsilyl)ethynyl)-7-((triisopropylsilyl)oxy)hepta-4,6-dienal **8a** (YH00101-22)

The title compound was prepared according to the procedure for the synthesis of **8a** (75%). **^{1}H NMR** (300 MHz, $CDCl_3$): δ 9.63 (d, *J* = 1.9 Hz, 1H), 7.29-7.13 (m, 5H), 6.11 (s, 1H), 3.41-3.36 (m, 4H), 3.27-3.16 (m, 2H), 2.55-2.46 (m, 1H), 2.33-2.21 (m, 3H), 1.70-1.66 (m, 4H), 1.09-1.01 (m, 42H). ppm. **^{13}C NMR** (75 MHz, $CDCl_3$): δ 204.19, 148.92, 139.19, 138.26, 129.24, 128.25, 126.17, 117.73, 111.25, 87.84, 77.22, 49.76, 44.11, 38.82, 26.56, 25.37, 18.77, 17.64, 11.84, 11.69. ppm. **HRMS** (**ESI-TOF**) calculated for C38H63NO2Si2H(+): 622.4476; Found: 622.4466.
